# Supplementary material for: Clinically observed RASA1 missense mutants exhibit diverse RasGAP protein behaviors in vitro
Source: J Biol Chem. 2026 May 27;302(7):113201. doi: 10.1016/j.jbc.2026.113201 (PMC13311836; doi:10.1016/j.jbc.2026.113201)
Supplement: Supporting Figures and Tables [file mmc1.pdf]

Supplementary information for:

Clinically observed *RASA1* missense mutants exhibit diverse RasGAP protein behaviors *in vitro*

Maxum E. Paul[1], Rediet B. Delelegne[2], Jocelyn E. Chau[1], and Titus J. Boggon[1,2,3+]

From:

[1]Department of Molecular Biophysics and Biochemistry, Yale University, New Haven, CT, USA 06520

[2]Department of Pharmacology, Yale University, New Haven, CT, USA 06510

[3]Yale Cancer Center, Yale University, New Haven, CT, USA 06510

+ To whom correspondence should be addressed: titus.boggon@yale.edu

Supplementary Table 1. *RASA1* missense mutants associated with vascular malformations. Missense mutations in *RASA1* associated with vascular malformations compiled from the literature. Shown are the nucleotide and amino acid mutations reported, frequency of mutation, phenotype in patients, and source study.

**Codon Change**	**Protein Mutation**	**Single Letter Code**	**# Mutations at residue**	**Phenotype**	**Source**
c.64G > T	p.Gly22Cys	G22C	2	Cutaneous CM, pial AVM, family history of termination of pregnancy at 7 months for VOGM	(1)
c.208G>C	p.Glu70Gln	E70Q	1	suspected HHT, lung AVM (VUS)	(2)
c.296C>T	p.Ala99Val	A99V	4	unilateral CMs, one patient had hypertrophic CM limb, another patient had a limb with CM that had a smaller circumference	(3)
c.346C>G	p.Leu116Val	L116V	1	CM on thigh, leg, foot, monocystic lymphatic malformation of the buttock, affected limb was hypertrophic and slightly longer (12mm) than other limb, dilated superficial veins	(3)
c.467G>T	p.Gly156Val	G156V	1	leg and thigh AVMs (VUS)	(2)
c.1277A > G	p.Tyr426Cys	Y426C	8	Cutaneous CM	(4)
c.1310T > G	p.Leu437Arg	L437R	2	Multifocal CMs on right palm, chest, and back, history of nosebleeds, son also had variant and multifocal CMs (VUS)	(5)
c.1352A>G	p.Asn451Ser	N451S	1	CMs on both les, legs had discrepant lengths, lymphedema on both feet	(3)
c.1583A > G	p.Tyr528Cys	Y528C	4	1) Multiple blanching telangiectasias on palms, fingers, tongue, and toes, erythematous nasal mucosa with daily epistaxis, family history of epistaxis (suspected HHT), 2) Cutaneous CM	(1,6)
c.1589T > A	p.Val530Asp	V530D	6	Cutaneous CM, brainstem AVM (brain hemorrhage with facial palsy at 3 weeks), left leg AVM, basal cell carcinoma in 2 individuals	(1)
c.1619G > A	p.Cys540Tyr	C540Y	9	CM-AVM1 (CM, Nuchal CM, AVF)	(7)
c.1649A>T	p.Glu550Val	E550V	1	Multiple CMs (VUS)	(2)
p.1834A>G	p.Thr612Ala	T612A	1	Suspected HHT, brain AVM (VUS)	(2)
c.1877C > A	p.Ala626Glu	A626E	1	Cutaneous CM	(1)
c.2084A > T	p.His695Leu	H695L	2	Brain AVF, brain AVM, multifocal CMs on head/ face, mother (non-symptomatic) carries mutation	(5,8)
c.2119C > T	p.Arg707Cys	R707C	4	1) VOGM, 2) Choroidal VOGM in two consecutive pregnancies of unaffected carrier mother	(9,10)
c.2209G>A	p.Val737Ile	V737I	1	Multiple CMs, telangiectasia (VUS)	(2)
c.2288A > T	p.Glu763Val	E763V	3	1) Cutaneous CM, VOGM, heart failure, epilepsy, died rapidly after birth, 2) Cutaneous CM	(1,4)
c.2912T > C	p.Leu971Ser	L971S	1	VOGM	(9)

Supplementary Table 2. Cancer associated *RASA1* missense mutants. Missense mutations in *RASA1* (associated with cancer and documented in cBioPortal on July 18, 2025). Redundancy is avoided by use of only a curated set of non-redundant studies in the analysis.

Codon Change	Protein Mutation	Single Letter Code	# Mutations at residue	Cancer Type	Source
c.20G>T	p.Gly7Val	G7V	1	Lung Adenocarcinoma	(11)
c.30G>T	p.Glu10Asp	E10D	1	Lung Adenocarcinoma	(12)
c.32G>A	p.Gly11Asp	G11D	1	Colon Adenocarcinoma	(12)
c.34G>T	p.Gly12Cys	G12C	2	Lung Adenocarcinoma	(12)
c.34G>C	p.Gly12Arg	G12R	2	Non-Small Cell Lung Cancer	(12)
c.37C>T	p.Pro13Ser	P13S	1	Breast Invasive Cancer, NOS	(13)
c.40G>T	p.Val14Leu	V14L	2	Breast Invasive Ductal Carcinoma	(14)
c.40G>T	p.Val14Leu	V14L	2	Breast Invasive Ductal Carcinoma	(12)
c.59G>A	p.Gly20Glu	G20E	3	Renal Clear Cell Carcinoma	(14)
c.59G>A	p.Gly20Glu	G20E	3	Renal Clear Cell Carcinoma	(14)
c.59G>T	p.Gly20Val	G20V	3	Lung Adenocarcinoma	(12)
c.70G>A	p.Ala24Thr	A24T	1	Colorectal Adenocarcinoma	(15)
c.74C>T	p.Ala25Val	A25V	1	Pancreatic Adenocarcinoma	(16)
c.77G>A	p.Gly26Asp	G26D	1	Prostate Adenocarcinoma	(12)
c.92C>A	p.Pro31His	P31H	1	Colon Adenocarcinoma	(12)
c.98T>C	p.Val33Ala	V33A	3	Colon Adenocarcinoma	(12)
c.98T>C	p.Val33Ala	V33A	3	Rectal Adenocarcinoma	(12)
c.97G>C	p.Val33Leu	V33L	3	Invasive Breast Carcinoma	(12)
c.101G>C	p.Cys34Ser	C34S	1	Lung Adenocarcinoma	(12)
c.103C>G	p.Arg35Gly	R35G	3	Lung Squamous Cell Carcinoma	(17,18)
c.104G>T	p.Arg35Leu	R35L	3	Lung Adenocarcinoma	(12)
c.104G>T	p.Arg35Leu	R35L	3	Head and Neck Squamous Cell Carcinoma	(19-24)
c.107T>G	p.Val36Gly	V36G	2	B-Lymphoblastic Leukemia/Lymphoma	(25)
c.107T>G	p.Val36Gly	V36G	2	Optic nerve glioma	(25)
c.112A>G	p.Ile38Val	I38V	1	Head and Neck Squamous Cell Carcinoma of Unknown Primary	(14)
c.119C>G	p.Ala40Gly	A40G	1	Lung Squamous Cell Carcinoma	(19-24,26)
c.133G>A	p.Ala45Thr	A45T	1	Cholangiocarcinoma	(27)
c.142_143delinsTT	p.Pro48Phe	P48F	2	Skin Cancer, Non-Melanoma	(28)
c.143C>A	p.Pro48His	P48H	2	Fibrolamellar Carcinoma	(14)
c.149C>A	p.Pro50His	P50H	1	Colon Adenocarcinoma	(12)
c.170T>C	p.Val57Ala	V57A	1	Colon Adenocarcinoma	(29)
c.173C>T	p.Ala58Val	A58V	1	Colorectal Adenocarcinoma	(12)
c.184G>T	p.Gly62Cys	G62C	2	Lung Adenocarcinoma	(14)
c.184G>T	p.Gly62Cys	G62C	2	Lung Adenocarcinoma	(12)
c.193G>A	p.Ala65Thr	A65T	1	Uterine Carcinosarcoma/Uterine Malignant Mixed Mullerian Tumor	(30)
c.210G>T	p.Glu70Asp	E70D	3	Endometrial Carcinoma	(31)
c.209A>G	p.Glu70Gly	E70G	3	Activated B-cell Type	(32)
c.208G>C	p.Glu70Gln	E70Q	3	Breast Invasive Ductal Carcinoma	(33)
c.214C>A	p.Leu72Ile	L72I	2	Cutaneous Melanoma	(19-24)
c.214C>A	p.Leu72Ile	L72I	2	Colon Adenocarcinoma	(12)
c.224G>A	p.Gly75Glu	G75E	1	Pancreatic Neuroendocrine Tumor	(12)
c.229G>A	p.Val77Met	V77M	1	Colon Adenocarcinoma	(12)
c.238G>A	p.Ala80Thr	A80T	1	Colon Adenocarcinoma	(12)
c.244G>C	p.Gly82Arg	G82R	1	Prostate Adenocarcinoma	(12)

c.248G>A	p.Gly83Glu	G83E	1	Cholangiocarcinoma	(27)
c.266G>C	p.Gly89Ala	G89A	2	Prostate Adenocarcinoma	(12)
c.265G>A	p.Gly89Arg	G89R	2	Adrenocortical Carcinoma	(33)
c.290G>C	p.Gly97Ala	G97A	1	Colorectal Adenocarcinoma	(12)
c.296C>T	p.Ala99Val	A99V	2	Germinal Center B-Cell Type	(32)
c.296C>T	p.Ala99Val	A99V	2	Germinal Center B-Cell Type	(32)
c.319G>C	p.Val107Leu	V107L	1	Poorly Differentiated Non-Small Cell Lung Cancer	(12)
c.322G>T	p.Ala108Ser	A108S	2	Colon Adenocarcinoma	(14)
c.322G>T	p.Ala108Ser	A108S	2	Colon Adenocarcinoma	(12)
c.341T>C	p.Met114Thr	M114T	1	Uterine Mixed Endometrial Carcinoma	(19-24,26)
c.365C>T	p.Ser122Leu	S122L	2	Bladder Urothelial Carcinoma	(19-24)
c.365C>T	p.Ser122Leu	S122L	2	Breast Invasive Ductal Carcinoma	(19-24)
c.370C>A	p.Leu124Ile	L124I	1	Uterine Endometrioid Carcinoma	(19-24,26)
c.376G>A	p.Glu126Lys	E126K	1	Lung Squamous Cell Carcinoma	(34)
c.379A>T	p.Thr127Ser	T127S	1	Uterine Endometrioid Carcinoma	(19-24,26)
c.383T>C	p.Leu128Pro	L128P	1	Endometrial Carcinoma	(35)
c.385G>T	p.Gly129Trp	G129W	1	Cutaneous Melanoma	(19-24)
c.389C>T	p.Pro130Leu	P130L	5	Cutaneous Melanoma	(19-24)
c.388_389delinsTT	p.Pro130Leu	P130L	5	Melanoma	(34)
c.388C>T	p.Pro130Ser	P130S	5	Cutaneous Melanoma	(19-24)
c.388C>T	p.Pro130Ser	P130S	5	Rectal Adenocarcinoma	(12)
c.388C>T	p.Pro130Ser	P130S	5	Colorectal Adenocarcinoma	(15)
c.398G>A	p.Gly133Asp	G133D	1	Invasive Breast Carcinoma	(36)
c.404C>A	p.Pro135His	P135H	3	Large Cell Neuroendocrine Carcinoma	(12)
c.403C>T	p.Pro135Ser	P135S	3	Cutaneous Melanoma	(37)
c.403C>T	p.Pro135Ser	P135S	3	Cutaneous Squamous Cell Carcinoma	(14)
c.407C>G	p.Pro136Arg	P136R	1	Lung Adenocarcinoma	(12)
c.409C>G	p.Leu137Val	L137V	3	Bladder Urothelial Carcinoma	(33)
c.409C>G	p.Leu137Val	L137V	3	Breast Invasive Lobular Carcinoma	(14)
c.409C>G	p.Leu137Val	L137V	3	Breast Invasive Lobular Carcinoma	(12)
c.422C>T	p.Pro141Leu	P141L	2	Small Cell Lung Cancer	(38)
c.422C>T	p.Pro141Leu	P141L	2	Small Cell Lung Cancer	(38)
c.431C>A	p.Pro144His	P144H	1	Cutaneous Melanoma	(19-24)
c.437T>C	p.Leu146Ser	L146S	2	Colon Adenocarcinoma	(19-24,26)
c.437T>G	p.Leu146Trp	L146W	2	Melanoma	(39)
c.439G>A	p.Gly147Arg	G147R	1	Renal Clear Cell Carcinoma	(19-24,26)
c.458T>C	p.Val153Ala	V153A	1	Non-Small Cell Lung Cancer	(12)
c.462C>G	p.Asp154Glu	D154E	3	Bladder Urothelial Carcinoma	(40)
c.462C>G	p.Asp154Glu	D154E	3	Bladder Urothelial Carcinoma	(41)
c.461A>G	p.Asp154Gly	D154G	3	Esophagogastric Adenocarcinoma	(42)
c.466G>T	p.Gly156Cys	G156C	1	Non-Small Cell Lung Cancer	(12)
c.471C>G	p.Asp157Glu	D157E	1	Lung Adenocarcinoma	(12)
c.473C>G	p.Ser158Cys	S158C	1	Lung Adenocarcinoma	(12)
c.475C>G	p.Leu159Val	L159V	2	Lung Adenocarcinoma	(12)
c.475C>G	p.Leu159Val	L159V	2	Bladder Urothelial Carcinoma	(41)
c.489A>T	p.Glu163Asp	E163D	1	Cutaneous Melanoma	(33)
c.502G>A	p.Glu168Lys	E168K	1	Bladder Urothelial Carcinoma	(41)
c.508G>T	p.Ala170Ser	A170S	3	Lung Adenocarcinoma	(14)
c.508G>T	p.Ala170Ser	A170S	3	Lung Adenocarcinoma	(12)
c.509C>T	p.Ala170Val	A170V	3	Colon Adenocarcinoma	(12)
c.519G>T	p.Leu173Phe	L173F	1	Lung Adenocarcinoma	(12)
c.524C>G	p.Ala175Gly	A175G	1	Prostate Adenocarcinoma	(43)
c.527C>A	p.Pro176His	P176H	3	Colon Adenocarcinoma	(12)
c.526C>T	p.Pro176Ser	P176S	3	Invasive Breast Carcinoma	(14)
c.526C>T	p.Pro176Ser	P176S	3	Invasive Breast Carcinoma	(12)

c.550G>A	p.Gly184Arg	G184R	1	Breast Invasive Cancer, NOS	(13)
c.562A>G	p.Arg188Gly	R188G	1	Colon Adenocarcinoma	(12)
c.566C>T	p.Thr189Met	T189M	1	Uterine Endometrioid Carcinoma	(19-24,26)
c.580C>T	p.Arg194Cys	R194C	11	Astrocytoma	(19-24)
c.580C>T	p.Arg194Cys	R194C	11	Colon Adenocarcinoma	(19-24,26)
c.580C>T	p.Arg194Cys	R194C	11	Endometrial Carcinoma	(31)
c.580C>T	p.Arg194Cys	R194C	11	Glioblastoma Multiforme	(19-24,26)
c.580C>T	p.Arg194Cys	R194C	11	Uterine Endometrioid Carcinoma	(19-24,26)
c.581G>A	p.Arg194His	R194H	11	Colon Adenocarcinoma	(19-24,26)
c.581G>A	p.Arg194His	R194H	11	Rectal Adenocarcinoma	(12)
c.581G>A	p.Arg194His	R194H	11	Colon Adenocarcinoma	(12)
c.581G>A	p.Arg194His	R194H	11	Uterine Serous Carcinoma/Uterine Papillary Serous Carcinoma	(19-24,26)
c.581G>A	p.Arg194His	R194H	11	Uterine Endometrioid Carcinoma	(19-24,26)
c.581G>A	p.Arg194His	R194H	11	Cervical Squamous Cell Carcinoma	(19-24,26)
c.584T>A	p.Leu195His	L195H	2	Non-Small Cell Lung Cancer	(14)
c.584T>A	p.Leu195His	L195H	2	Non-Small Cell Lung Cancer	(12)
c.613C>T	p.Leu205Phe	L205F	1	Breast Invasive Lobular Carcinoma	(12)
c.616A>G	p.Ile206Val	I206V	3	Hepatocellular Carcinoma plus Intrahepatic Cholangiocarcinoma	(44)
c.616A>G	p.Ile206Val	I206V	3	Hepatocellular Carcinoma plus Intrahepatic Cholangiocarcinoma	(44)
c.616A>G	p.Ile206Val	I206V	3	Lung Squamous Cell Carcinoma	(12)
c.624G>C	p.Glu208Asp	E208D	1	Lung Adenocarcinoma	(12)
c.632G>T	p.Arg211Leu	R211L	4	Endometrial Carcinoma	(31)
c.632G>A	p.Arg211Gln	R211Q	4	Colon Adenocarcinoma	(14)
c.632G>A	p.Arg211Gln	R211Q	4	Colon Adenocarcinoma	(12)
c.631C>T	p.Arg211Trp	R211W	4	Lung Squamous Cell Carcinoma	(12)
c.635G>T	p.Arg212Met	R212M	3	Uterine Endometrioid Carcinoma	(19-24,26)
c.634A>T	p.Arg212Trp	R212W	3	Lung Adenocarcinoma	(14)
c.634A>T	p.Arg212Trp	R212W	3	Lung Adenocarcinoma	(12)
c.637C>T	p.Pro213Ser	P213S	2	Basaloid Penile Squamous Cell Carcinoma	(14)
c.637C>T	p.Pro213Ser	P213S	2	Colorectal Adenocarcinoma	(15)
c.652C>T	p.Leu218Phe	L218F	1	Colorectal Adenocarcinoma	(15)
c.656C>T	p.Ser219Leu	S219L	1	Bladder Urothelial Carcinoma	(19-24)
c.661C>G	p.Leu221Val	L221V	1	Lung Adenocarcinoma	(12)
c.665G>A	p.Ser222Asn	S222N	1	Colorectal Adenocarcinoma	(45)
c.672G>T	p.Met224Ile	M224I	2	Colon Adenocarcinoma	(12)
c.672G>T	p.Met224Ile	M224I	2	Uterine Endometrioid Carcinoma	(19-24,26)
c.677T>C	p.Val226Ala	V226A	2	Intrahepatic Cholangiocarcinoma	(14)
c.676G>C	p.Val226Leu	V226L	2	Lung Squamous Cell Carcinoma	(12)
c.685C>T	p.His229Tyr	H229Y	1	Uterine Endometrioid Carcinoma	(19-24,26)
c.704T>C	p.Met235Thr	M235T	1	Colon Adenocarcinoma	(12)
c.710G>C	p.Gly237Ala	G237A	1	Ampullary Carcinoma	(14)
c.725G>A	p.Gly242Asp	G242D	1	Colorectal Adenocarcinoma	(12)
c.727G>A	p.Gly243Arg	G243R	1	Prostate	(46)
c.733C>T	p.Arg245Cys	R245C	6	Lung Squamous Cell Carcinoma	(12)
c.733C>T	p.Arg245Cys	R245C	6	Tubular Stomach Adenocarcinoma	(19-24,26)
c.733C>G	p.Arg245Gly	R245G	6	Breast Invasive Ductal Carcinoma	(19-24)
c.734G>A	p.Arg245His	R245H	6	Cutaneous Melanoma	(47)
c.734G>A	p.Arg245His	R245H	6	Uterine Endometrioid Carcinoma	(14)
c.733C>A	p.Arg245Ser	R245S	6	Yolk Sac Tumor	(33)
c.740C>A	p.Ser247Tyr	S247Y	2	Breast Mixed Ductal and Lobular Carcinoma	(14)
c.740C>A	p.Ser247Tyr	S247Y	2	Breast Mixed Ductal and Lobular Carcinoma	(12)
c.751G>C	p.Asp251His	D251H	1	Breast Invasive Ductal Carcinoma	(19-24)
c.764A>T	p.Tyr255Phe	Y255F	2	Breast Invasive Ductal Carcinoma	(19-24)

c.763T>C	p.Tyr255His	Y255H	2	Endometrial Carcinoma	(31)
c.770G>A	p.Ser257Asn	S257N	2	Endometrial Carcinoma	(48)
c.769A>C	p.Ser257Arg	S257R	2	Lung Adenocarcinoma	(12)
c.779C>G	p.Ser260Cys	S260C	1	Lung Squamous Cell Carcinoma	(19-24,26)
c.787C>T	p.Leu263Phe	L263F	1	Hepatocellular Adenoma	(49)
c.823C>G	p.Pro275Ala	P275A	1	Glioblastoma	(50)
c.842G>T	p.Arg281Ile	R281I	2	Colon Adenocarcinoma	(19-24,26)
c.842G>T	p.Arg281Ile	R281I	2	Colorectal Adenocarcinoma	(34)
c.847C>T	p.Arg283Cys	R283C	14	Cholangiocarcinoma	(27)
c.847C>T	p.Arg283Cys	R283C	14	Colon Adenocarcinoma	(12)
c.847C>T	p.Arg283Cys	R283C	14	Colon Adenocarcinoma	(12)
c.847C>T	p.Arg283Cys	R283C	14	Lung Squamous Cell Carcinoma	(19-24,26)
c.848G>A	p.Arg283His	R283H	14	Colorectal Adenocarcinoma	(51)
c.848G>A	p.Arg283His	R283H	14	Colon Adenocarcinoma	(14)
c.848G>A	p.Arg283His	R283H	14	Rectal Adenocarcinoma	(14)
c.848G>A	p.Arg283His	R283H	14	Endometrial Carcinoma	(31)
c.848G>A	p.Arg283His	R283H	14	Colon Adenocarcinoma	(12)
c.848G>A	p.Arg283His	R283H	14	Rectal Adenocarcinoma	(12)
c.848G>A	p.Arg283His	R283H	14	Colon Adenocarcinoma	(12)
c.848G>A	p.Arg283His	R283H	14	Lung Adenocarcinoma	(52)
c.848G>A	p.Arg283His	R283H	14	Intestinal Type Stomach Adenocarcinoma	(19-24,26)
c.848G>A	p.Arg283His	R283H	14	Ampullary Carcinoma	(53)
c.853C>G	p.Arg285Gly	R285G	5	Lung Squamous Cell Carcinoma	(12)
c.854G>C	p.Arg285Pro	R285P	5	Lung Squamous Cell Carcinoma	(12)
c.854G>A	p.Arg285Gln	R285Q	5	Rectal Adenocarcinoma	(19-24,26)
c.854G>A	p.Arg285Gln	R285Q	5	Uterine Endometrioid Carcinoma	(19-24,26)
c.854G>A	p.Arg285Gln	R285Q	5	Uterine Endometrioid Carcinoma	(19-24,26)
c.866C>T	p.Pro289Leu	P289L	1	Skin Cancer, Non-Melanoma	(28)
c.869A>G	p.Tyr290Cys	Y290C	2	Prostate Adenocarcinoma	(12)
c.869A>C	p.Tyr290Ser	Y290S	2	Pancreatic Neuroendocrine Tumor	(12)
c.883G>A	p.Asp295Asn	D295N	1	Lung Adenocarcinoma	(14)
c.899G>T	p.Ser300Ile	S300I	4	Lung Adenosquamous Carcinoma	(17,18)
c.899G>T	p.Ser300Ile	S300I	4	Lung Adenosquamous Carcinoma	(17,18)
c.899G>T	p.Ser300Ile	S300I	4	Lung Adenosquamous Carcinoma	(17,18)
c.899G>T	p.Ser300Ile	S300I	4	Lung Adenosquamous Carcinoma	(17,18)
c.903C>A	p.Phe301Leu	F301L	1	Bladder Urothelial Carcinoma	(19-24)
c.906A>T	p.Leu302Phe	L302F	1	Lung Adenocarcinoma	(14)
c.915T>G	p.Asp305Glu	D305E	1	Mucinous Adenocarcinoma of the Colon and Rectum	(12)
c.921C>G	p.Phe307Leu	F307L	2	Breast Mixed Ductal and Lobular Carcinoma	(12)
c.919T>C	p.Phe307Leu	F307L	2	Rectal Adenocarcinoma	(12)
c.923T>A	p.Ile308Asn	I308N	1	Mucinous Adenocarcinoma of the Colon and Rectum	(12)
c.939A>T	p.Leu313Phe	L313F	1	Melanoma	(34)
c.940G>C	p.Glu314Gln	E314Q	1	Glioblastoma Multiforme	(14)
c.945T>A	p.Asp315Glu	D315E	3	Invasive Breast Carcinoma	(12)
c.943G>A	p.Asp315Asn	D315N	3	Cutaneous Melanoma	(14)
c.943G>A	p.Asp315Asn	D315N	3	Cutaneous Melanoma	(14)
c.957G>T	p.Trp319Cys	W319C	1	Colorectal Adenocarcinoma	(54)
c.964A>C	p.Asn322His	N322H	4	Mucinous Adenocarcinoma of the Appendix	(14)
c.964A>C	p.Asn322His	N322H	4	Uterine Endometrioid Carcinoma	(19-24,26)
c.966T>G	p.Asn322Lys	N322K	4	Colon Adenocarcinoma	(12)
c.965A>G	p.Asn322Ser	N322S	4	Lung Adenocarcinoma	(52)
c.967T>G	p.Leu323Val	L323V	1	Colon Adenocarcinoma	(12)
c.971G>A	p.Arg324Lys	R324K	3	Colon Adenocarcinoma	(12)
c.972A>T	p.Arg324Ser	R324S	3	Lung Adenocarcinoma	(14)

c.972A>T	p.Arg324Ser	R324S	3	Lung Adenocarcinoma	(12)
c.974C>G	p.Thr325Arg	T325R	1	Breast Invasive Ductal Carcinoma	(19-24)
c.976G>A	p.Asp326Asn	D326N	2	Hepatocellular Carcinoma	(19-24,26)
c.976G>T	p.Asp326Tyr	D326Y	2	Uterine Endometrioid Carcinoma	(19-24,26)
c.992T>C	p.Ile331Thr	I331T	1	Plasma Cell Myeloma	(55)
c.997G>C	p.Glu333Gln	E333Q	1	Glioblastoma Multiforme	(14)
c.1022G>A	p.Arg341Gln	R341Q	2	High-Grade Serous Ovarian Cancer	(14)
c.1021C>T	p.Arg341Trp	R341W	2	Lung Adenocarcinoma	(12)
c.1024G>A	p.Glu342Lys	E342K	2	Uterine Endometrioid Carcinoma	(14)
c.1024G>A	p.Glu342Lys	E342K	2	Non-Small Cell Lung Cancer	(12)
c.1043G>C	p.Gly348Ala	G348A	2	Cutaneous Squamous Cell Carcinoma	(14)
c.1042G>A	p.Gly348Arg	G348R	2	Uterine Mixed Endometrial Carcinoma	(19-24,26)
c.1072A>G	p.Lys358Glu	K358E	2	Lung Adenocarcinoma	(14)
c.1072A>G	p.Lys358Glu	K358E	2	Lung Adenocarcinoma	(12)
c.1087A>C	p.Asn363His	N363H	1	Hepatocellular Carcinoma plus Intrahepatic Cholangiocarcinoma	(44)
c.1098G>C	p.Met366Ile	M366I	1	Esophageal Adenocarcinoma	(34)
c.1100C>A	p.Thr367Lys	T367K	1	Non-Small Cell Lung Cancer	(12)
c.1102G>T	p.Val368Phe	V368F	2	Cutaneous Melanoma	(19-24)
c.1102G>A	p.Val368Ile	V368I	2	Lung Squamous Cell Carcinoma	(19-24,26)
c.1105G>A	p.Gly369Ser	G369S	1	Leiomyosarcoma	(19-24,26)
c.1115G>T	p.Cys372Phe	C372F	1	Cholangiocarcinoma	(27)
c.1123C>G	p.Leu375Val	L375V	1	Bladder Urothelial Carcinoma	(19-24)
c.1133C>T	p.Pro378Leu	P378L	1	Endometrial Carcinoma	(31)
c.1136C>T	p.Ser379Leu	S379L	3	Non-Small Cell Lung Cancer	(12)
c.1136C>T	p.Ser379Leu	S379L	3	Lung Adenocarcinoma	(12)
c.1136C>T	p.Ser379Leu	S379L	3	Lung Adenocarcinoma	(12)
c.1139A>G	p.Asp380Gly	D380G	2	Paraganglioma	(14)
c.1138G>A	p.Asp380Asn	D380N	2	Skin	(56-59)
c.1145C>G	p.Thr382Ser	T382S	1	Uterine Clear Cell Carcinoma	(60)
c.1151G>A	p.Gly384Asp	G384D	1	Colon Adenocarcinoma	(12)
c.1156T>C	p.Tyr386His	Y386H	1	Colon Adenocarcinoma	(12)
c.1160C>T	p.Ser387Leu	S387L	1	Endocervical Adenocarcinoma	(14)
c.1163T>G	p.Leu388Arg	L388R	1	Lung Squamous Cell Carcinoma	(12)
c.1171C>T	p.Arg391Trp	R391W	7	Esophageal Adenocarcinoma	(19-24)
c.1171C>T	p.Arg391Trp	R391W	7	Colon Adenocarcinoma	(14)
c.1171C>T	p.Arg391Trp	R391W	7	Endometrial Carcinoma	(31)
c.1171C>T	p.Arg391Trp	R391W	7	Colon Adenocarcinoma	(12)
c.1171C>T	p.Arg391Trp	R391W	7	Colon Adenocarcinoma	(12)
c.1171C>T	p.Arg391Trp	R391W	7	Endometrial Carcinoma	(35)
c.1171C>T	p.Arg391Trp	R391W	7	Colorectal Adenocarcinoma	(15)
c.1190A>C	p.Gln397Pro	Q397P	1	Invasive Breast Carcinoma	(12)
c.1193G>T	p.Arg398Leu	R398L	8	Colorectal Adenocarcinoma	(12)
c.1193G>A	p.Arg398Gln	R398Q	8	Colon Adenocarcinoma	(14)
c.1193G>A	p.Arg398Gln	R398Q	8	Upper Tract Urothelial Carcinoma	(14)
c.1193G>A	p.Arg398Gln	R398Q	8	Uterine Endometrioid Carcinoma	(14)
c.1193G>A	p.Arg398Gln	R398Q	8	Colon Adenocarcinoma	(12)
c.1193G>A	p.Arg398Gln	R398Q	8	Non-Small Cell Lung Cancer	(12)
c.1193G>A	p.Arg398Gln	R398Q	8	Uterine Endometrioid Carcinoma	(19-24,26)
c.1193G>A	p.Arg398Gln	R398Q	8	Uterine Carcinosarcoma/Uterine Malignant Mixed Mullerian Tumor	(30)
c.1207C>G	p.Pro403Ala	P403A	3	Lung Squamous Cell Carcinoma	(19-24,26)
c.1208C>T	p.Pro403Leu	P403L	3	Intestinal Type Stomach Adenocarcinoma	(19-24,26)
c.1208C>A	p.Pro403Gln	P403Q	3	Lung Adenocarcinoma	(12)
c.1210A>T	p.Thr404Ser	T404S	2	Papillary Renal Cell Carcinoma	(19-24)
c.1210A>T	p.Thr404Ser	T404S	2	Papillary Renal Cell Carcinoma	(34)

c.1214C>G	p.Pro405Arg	P405R	1	Prostate Adenocarcinoma	(12)
c.1224G>T	p.Gln408His	Q408H	1	Uterine Endometrioid Carcinoma	(14)
c.1230G>A	p.Met410Ile	M410I	1	Leiomyosarcoma	(19-24,26)
c.1234G>A	p.Gly412Arg	G412R	2	Spindle Cell Carcinoma of the Lung	(14)
c.1234G>A	p.Gly412Arg	G412R	2	Spindle Cell Carcinoma of the Lung	(12)
c.1241G>T	p.Arg414Leu	R414L	7	Lung Adenocarcinoma	(12)
c.1241G>A	p.Arg414Gln	R414Q	7	Cutaneous Squamous Cell Carcinoma	(14)
c.1241G>A	p.Arg414Gln	R414Q	7	Lung Adenocarcinoma	(14)
c.1241G>A	p.Arg414Gln	R414Q	7	Lung Adenocarcinoma	(12)
c.1241G>A	p.Arg414Gln	R414Q	7	Uterine Endometrioid Carcinoma	(19-24,26)
c.1240C>T	p.Arg414Trp	R414W	7	Medullary Carcinoma of the Colon	(12)
c.1240C>T	p.Arg414Trp	R414W	7	Colon Adenocarcinoma	(12)
c.1244A>T	p.Tyr415Phe	Y415F	1	Poorly Differentiated Thyroid Cancer	(14)
c.1251C>A	p.Asn417Lys	N417K	1	Lung Adenocarcinoma	(12)
c.1253G>C	p.Ser418Thr	S418T	1	Lung Adenocarcinoma	(19-24)
c.1262A>G	p.Asp421Gly	D421G	2	Upper Tract Urothelial Carcinoma	(61)
c.1262A>G	p.Asp421Gly	D421G	2	Upper Tract Urothelial Carcinoma	(14)
c.1268T>C	p.Ile423Thr	I423T	1	Hepatocellular Carcinoma	(62)
c.1272T>G	p.Asp424Glu	D424E	2	Hepatocellular Carcinoma	(62)
c.1270G>T	p.Asp424Tyr	D424Y	2	Colon Adenocarcinoma	(12)
c.1273C>A	p.His425Asn	H425N	2	Lung Adenosquamous Carcinoma	(12)
c.1273C>T	p.His425Tyr	H425Y	2	Lung Adenocarcinoma	(63)
c.1277A>G	p.Tyr426Cys	Y426C	2	Endometrial Carcinoma	(48)
c.1276T>C	p.Tyr426His	Y426H	2	Uterine Clear Cell Carcinoma	(14)
c.1280G>A	p.Arg427Gln	R427Q	13	Endometrial Carcinoma	(48)
c.1280G>A	p.Arg427Gln	R427Q	13	Mucinous Adenocarcinoma of the Colon and Rectum	(19-24,26)
c.1280G>A	p.Arg427Gln	R427Q	13	Colon Adenocarcinoma	(64)
c.1280G>A	p.Arg427Gln	R427Q	13	Colon Adenocarcinoma	(14)
c.1280G>A	p.Arg427Gln	R427Q	13	Basaloid Penile Squamous Cell Carcinoma	(14)
c.1280G>A	p.Arg427Gln	R427Q	13	Uterine Endometrioid Carcinoma	(14)
c.1280G>A	p.Arg427Gln	R427Q	13	Mucinous Adenocarcinoma of the Colon and Rectum	(34)
c.1280G>A	p.Arg427Gln	R427Q	13	Colon Adenocarcinoma	(12)
c.1280G>A	p.Arg427Gln	R427Q	13	Non-Small Cell Lung Cancer	(12)
c.1280G>A	p.Arg427Gln	R427Q	13	Uterine Endometrioid Carcinoma	(19-24,26)
c.1280G>A	p.Arg427Gln	R427Q	13	Uterine Endometrioid Carcinoma	(19-24,26)
c.1280G>A	p.Arg427Gln	R427Q	13	Uterine Endometrioid Carcinoma	(19-24,26)
c.1280G>A	p.Arg427Gln	R427Q	13	Colorectal Adenocarcinoma	(15)
c.1292T>C	p.Ile431Thr	I431T	2	Uterine Endometrioid Carcinoma	(14)
c.1292T>C	p.Ile431Thr	I431T	2	Endometrial Carcinoma	(31)
c.1301G>A	p.Gly434Glu	G434E	3	Renal Clear Cell Carcinoma	(14)
c.1301G>A	p.Gly434Glu	G434E	3	Non-Small Cell Lung Cancer	(12)
c.1301G>T	p.Gly434Val	G434V	3	Rectal Adenocarcinoma	(19-24,26)
c.1325C>A	p.Pro442Gln	P442Q	4	Cutaneous Melanoma	(19-24)
c.1325C>A	p.Pro442Gln	P442Q	4	Cutaneous Melanoma	(19-24)
c.1325C>A	p.Pro442Gln	P442Q	4	Cutaneous Melanoma	(19-24)
c.1324C>T	p.Pro442Ser	P442S	4	Uterine Carcinosarcoma/Uterine Malignant Mixed Mullerian Tumor	(19-24)
c.1332G>T	p.Gln444His	Q444H	1	Cutaneous Melanoma	(19-24)
c.1336C>G	p.Gln446Glu	Q446E	1	Head and Neck Squamous Cell Carcinoma	(19-24)
c.1343A>G	p.Gln448Arg	Q448R	1	Cutaneous Melanoma	(14)
c.1345G>T	p.Val449Leu	V449L	1	Cervical Squamous Cell Carcinoma	(19-24,26)
c.1352A>G	p.Asn451Ser	N451S	1	Osteosarcoma	(65)
c.1360G>A	p.Val454Met	V454M	1	Lung Squamous Cell Carcinoma	(12)
c.1363G>T	p.Asp455Tyr	D455Y	1	Esophageal Squamous Cell Carcinoma	(19-24)

c.1370A>G	p.Lys457Arg	K457R	1	Renal Clear Cell Carcinoma	(66)
c.1376T>C	p.Ile459Thr	I459T	1	Uterine Clear Cell Carcinoma	(14)
c.1390C>T	p.Arg464Cys	R464C	6	Endometrial Carcinoma	(31)
c.1390C>T	p.Arg464Cys	R464C	6	Breast Invasive Carcinoma, NOS	(12)
c.1390C>T	p.Arg464Cys	R464C	6	Intestinal Type Stomach Adenocarcinoma	(19-24,26)
c.1391G>A	p.Arg464His	R464H	6	Colon Adenocarcinoma	(12)
c.1391G>A	p.Arg464His	R464H	6	Colorectal Adenocarcinoma	(15)
c.1391G>A	p.Arg464His	R464H	6	Colorectal Adenocarcinoma	(15)
c.1394G>A	p.Arg465His	R465H	2	Colon Adenocarcinoma	(19-24,26)
c.1394G>A	p.Arg465His	R465H	2	Prostate Adenocarcinoma	(43)
c.1400C>G	p.Thr467Arg	T467R	2	Breast Invasive Ductal Carcinoma	(14)
c.1400C>G	p.Thr467Arg	T467R	2	Breast Invasive Ductal Carcinoma	(12)
c.1402A>G	p.Lys468Glu	K468E	4	Lung Adenocarcinoma	(12)
c.1404G>C	p.Lys468Asn	K468N	4	Cutaneous Melanoma	(19-24)
c.1404G>T	p.Lys468Asn	K468N	4	Colon Adenocarcinoma	(19-24,26)
c.1402A>C	p.Lys468Gln	K468Q	4	Uterine Endometrioid Carcinoma	(19-24,26)
c.1405G>A	p.Asp469Asn	D469N	1	Skin Cancer, Non-Melanoma	(28)
c.1409C>T	p.Ala470Val	A470V	1	Glioblastoma	(50)
c.1441C>T	p.Leu481Phe	L481F	3	Cervical Squamous Cell Carcinoma	(19-24,26)
c.1442T>C	p.Leu481Pro	L481P	3	Adenoid Cystic Breast Cancer	(67)
c.1442T>C	p.Leu481Pro	L481P	3	Adenoid Cystic Breast Cancer	(68)
c.1453G>T	p.Gly485Cys	G485C	2	Colon Adenocarcinoma	(14)
c.1453G>T	p.Gly485Cys	G485C	2	Colon Adenocarcinoma	(12)
c.1460G>A	p.Gly487Glu	G487E	1	Prostate Adenocarcinoma	(12)
c.1465C>T	p.Arg489Cys	R489C	3	Oligoastrocytoma	(19-24)
c.1465C>T	p.Arg489Cys	R489C	3	Colon Adenocarcinoma	(12)
c.1465C>T	p.Arg489Cys	R489C	3	Lung Adenocarcinoma	(19-24)
c.1471A>G	p.Lys491Glu	K491E	3	Rectal Adenocarcinoma	(19-24,26)
c.1472A>C	p.Lys491Thr	K491T	3	Prostate Adenocarcinoma	(14)
c.1472A>C	p.Lys491Thr	K491T	3	Prostate Adenocarcinoma	(12)
c.1474A>G	p.Asn492Asp	N492D	1	Rectal Adenocarcinoma	(12)
c.1480T>C	p.Tyr494His	Y494H	2	Colon Adenocarcinoma	(14)
c.1480T>C	p.Tyr494His	Y494H	2	Colon Adenocarcinoma	(12)
c.1494G>T	p.Glu498Asp	E498D	2	Lung Squamous Cell Carcinoma	(19-24,26)
c.1494G>T	p.Glu498Asp	E498D	2	Uterine Endometrioid Carcinoma	(19-24,26)
c.1502A>G	p.Asp501Gly	D501G	2	Papillary Renal Cell Carcinoma	(19-24)
c.1501G>T	p.Asp501Tyr	D501Y	2	Colon Adenocarcinoma	(12)
c.1511T>C	p.Leu504Pro	L504P	1	Intrahepatic Cholangiocarcinoma	(69)
c.1523A>T	p.Glu508Val	E508V	1	Thymoma	(19-24)
c.1526G>A	p.Ser509Asn	S509N	2	Cutaneous Melanoma	(19-24)
c.1526G>A	p.Ser509Asn	S509N	2	Melanoma	(34)
c.1528G>A	p.Glu510Lys	E510K	1	Cutaneous Melanoma	(37)
c.1535G>A	p.Arg512Gln	R512Q	3	Colon Adenocarcinoma	(14)
c.1535G>A	p.Arg512Gln	R512Q	3	Colon Adenocarcinoma	(12)
c.1535G>A	p.Arg512Gln	R512Q	3	Colon Adenocarcinoma	(12)
c.1561G>T	p.Asp521Tyr	D521Y	1	Uterine Endometrioid Carcinoma	(19-24,26)
c.1564C>T	p.Leu522Phe	L522F	2	Colorectal Adenocarcinoma	(15)
c.1565T>A	p.Leu522His	L522H	2	Lung Adenocarcinoma	(12)
c.1567A>G	p.Ser523Gly	S523G	2	Melanoma	(70)
c.1568G>A	p.Ser523Asn	S523N	2	Colon Adenocarcinoma	(12)
c.1571T>C	p.Val524Ala	V524A	1	Colon Adenocarcinoma	(12)
c.1573T>C	p.Cys525Arg	C525R	1	Lung Adenocarcinoma	(12)
c.1577C>A	p.Ser526Tyr	S526Y	2	Colorectal Adenocarcinoma	(14)
c.1577C>A	p.Ser526Tyr	S526Y	2	Colon Adenocarcinoma	(12)
c.1582T>C	p.Tyr528His	Y528H	1	Intestinal Type Stomach Adenocarcinoma	(19-24,26)

c.1588G>A	p.Val530Ile	V530I	1	Undifferentiated Pleomorphic Sarcoma/Malignant Fibrous Histiocytoma/High-Grade Spindle Cell Sarcoma	(14)
c.1592A>G	p.His531Arg	H531R	1	Endometrial Carcinoma	(48)
c.1600C>A	p.Leu534Ile	L534I	1	Colon Adenocarcinoma	(19-24,26)
c.1604T>C	p.Phe535Ser	F535S	1	Small Cell Lung Cancer	(71)
c.1607G>A	p.Gly536Asp	G536D	1	Prostate Adenocarcinoma	(12)
c.1609A>G	p.Arg537Gly	R537G	3	Lung Adenocarcinoma	(12)
c.1610G>T	p.Arg537Met	R537M	3	Lung Squamous Cell Carcinoma	(14)
c.1610G>T	p.Arg537Met	R537M	3	Lung Squamous Cell Carcinoma	(12)
c.1612C>T	p.Pro538Ser	P538S	1	Pancreatic Neuroendocrine Tumor	(12)
c.1622T>G	p.Phe541Cys	F541C	1	Intrahepatic Cholangiocarcinoma	(14)
c.1624C>G	p.Gln542Glu	Q542E	1	Lung Adenosquamous Carcinoma	(14)
c.1629A>G	p.Ile543Met	I543M	1	Prostate	(46)
c.1653A>C	p.Glu551Asp	E551D	3	Colorectal Adenocarcinoma	(14)
c.1653A>C	p.Glu551Asp	E551D	3	Colon Adenocarcinoma	(12)
c.1652A>T	p.Glu551Val	E551V	3	Breast Invasive Ductal Carcinoma	(12)
c.1662C>G	p.Ile554Met	I554M	2	Lung Adenocarcinoma	(14)
c.1662C>G	p.Ile554Met	I554M	2	Lung Adenocarcinoma	(12)
c.1663T>C	p.Phe555Leu	F555L	1	Glioblastoma Multiforme	(14)
c.1667A>G	p.Tyr556Cys	Y556C	1	Colon Adenocarcinoma	(12)
c.1684C>G	p.Pro562Ala	P562A	1	Lung Adenocarcinoma	(12)
c.1690C>A	p.Gln564Lys	Q564K	2	Colon Adenocarcinoma	(12)
c.1691A>G	p.Gln564Arg	Q564R	2	Hepatocellular Carcinoma	(62)
c.1714C>A	p.Leu572Met	L572M	1	Esophagogastric Adenocarcinoma	(42)
c.1720G>A	p.Ala574Thr	A574T	2	Lung Adenocarcinoma	(14)
c.1720G>A	p.Ala574Thr	A574T	2	Lung Adenocarcinoma	(12)
c.1736G>A	p.Arg579Gln	R579Q	10	Upper Tract Urothelial Carcinoma	(72)
c.1736G>A	p.Arg579Gln	R579Q	10	Upper Tract Urothelial Carcinoma	(72)
c.1736G>A	p.Arg579Gln	R579Q	10	Upper Tract Urothelial Carcinoma	(72)
c.1736G>A	p.Arg579Gln	R579Q	10	Upper Tract Urothelial Carcinoma	(72)
c.1736G>A	p.Arg579Gln	R579Q	10	Upper Tract Urothelial Carcinoma	(72)
c.1736G>A	p.Arg579Gln	R579Q	10	Upper Tract Urothelial Carcinoma	(72)
c.1735C>T	p.Arg579Trp	R579W	10	Colon Adenocarcinoma	(14)
c.1735C>T	p.Arg579Trp	R579W	10	Adrenocortical Carcinoma	(14)
c.1735C>T	p.Arg579Trp	R579W	10	Desmoplastic Small-Round-Cell Tumor	(14)
c.1735C>T	p.Arg579Trp	R579W	10	Endometrial Carcinoma	(31)
c.1740A>C	p.Lys580Asn	K580N	2	Colon Adenocarcinoma	(14)
c.1740A>C	p.Lys580Asn	K580N	2	Colon Adenocarcinoma	(12)
c.1756T>C	p.Ser586Pro	S586P	1	Colon Adenocarcinoma	(12)
c.1760A>G	p.Asn587Ser	N587S	2	Colorectal Adenocarcinoma	(14)
c.1760A>G	p.Asn587Ser	N587S	2	Rectal Adenocarcinoma	(12)
c.1765C>T	p.Arg589Cys	R589C	7	Endometrial Carcinoma	(48)
c.1765C>T	p.Arg589Cys	R589C	7	Esophageal Adenocarcinoma	(34)
c.1766G>A	p.Arg589His	R589H	7	Colon Adenocarcinoma	(29)
c.1766G>A	p.Arg589His	R589H	7	Colon Adenocarcinoma	(12)
c.1766G>A	p.Arg589His	R589H	7	Prostate Adenocarcinoma	(12)
c.1766G>A	p.Arg589His	R589H	7	Colon Adenocarcinoma	(12)
c.1766G>A	p.Arg589His	R589H	7	Uterine Endometrioid Carcinoma	(19-24,26)
c.1771C>T	p.Arg591Cys	R591C	13	Ganglioglioma	(73)
c.1771C>T	p.Arg591Cys	R591C	13	Angiosarcoma	(74)
c.1771C>T	p.Arg591Cys	R591C	13	Colon Adenocarcinoma	(64)
c.1771C>T	p.Arg591Cys	R591C	13	Angiosarcoma	(74)
c.1771C>T	p.Arg591Cys	R591C	13	Uterine Endometrioid Carcinoma	(14)
c.1771C>T	p.Arg591Cys	R591C	13	Colon Adenocarcinoma	(12)

c.1771C>T	p.Arg591Cys	R591C	13	Glioblastoma Multiforme	(19-24,26)
c.1771C>T	p.Arg591Cys	R591C	13	Uterine Endometrioid Carcinoma	(19-24,26)
c.1771C>T	p.Arg591Cys	R591C	13	Stomach Adenocarcinoma	(19-24,26)
c.1771C>G	p.Arg591Gly	R591G	13	Hepatocellular Carcinoma	(19-24,26)
c.1772G>A	p.Arg591His	R591H	13	Angiosarcoma	(14)
c.1772G>A	p.Arg591His	R591H	13	Colon Adenocarcinoma	(12)
c.1772G>A	p.Arg591His	R591H	13	Diffuse Type Stomach Adenocarcinoma	(19-24,26)
c.1774C>G	p.Gln592Glu	Q592E	1	Lung Adenocarcinoma	(75)
c.1778T>G	p.Val593Gly	V593G	2	Pancreatic Adenocarcinoma	(14)
c.1778T>G	p.Val593Gly	V593G	2	Pancreatic Adenocarcinoma	(12)
c.1786C>A	p.Leu596Ile	L596I	1	Colon Adenocarcinoma	(12)
c.1793T>C	p.Leu598Ser	L598S	1	Esophageal Adenocarcinoma	(19-24)
c.1799T>A	p.Ile600Asn	I600N	1	Lung Adenocarcinoma	(19-24)
c.1806A>C	p.Glu602Asp	E602D	3	Cancer of Unknown Primary	(14)
c.1806A>C	p.Glu602Asp	E602D	3	Colon Adenocarcinoma	(12)
c.1804G>A	p.Glu602Lys	E602K	3	Colon Adenocarcinoma	(12)
c.1810C>A	p.His604Asn	H604N	1	Uterine Endometrioid Carcinoma	(76)
c.1817T>A	p.Leu606His	L606H	1	Head and Neck Squamous Cell Carcinoma	(19-24)
c.1819C>T	p.Pro607Ser	P607S	1	Pancreatic Adenocarcinoma	(12)
c.1825A>C	p.Lys609Gln	K609Q	1	Rectal Adenocarcinoma	(19-24,26)
c.1837A>G	p.Asn613Asp	N613D	1	Colon Adenocarcinoma	(12)
c.1840C>T	p.Pro614Ser	P614S	1	Bladder Urothelial Carcinoma	(19-24)
c.1846T>C	p.Cys616Arg	C616R	3	Endometrial Carcinoma	(31)
c.1846T>C	p.Cys616Arg	C616R	3	Lung Squamous Cell Carcinoma	(12)
c.1847G>A	p.Cys616Tyr	C616Y	3	Colorectal Adenocarcinoma	(12)
c.1858C>A	p.Leu620Met	L620M	1	Colon Adenocarcinoma	(12)
c.1864A>G	p.Ser622Gly	S622G	1	Chondroblastic Osteosarcoma	(25)
c.1873G>T	p.Val625Leu	V625L	1	Uterine Endometrioid Carcinoma	(19-24,26)
c.1881A>C	p.Lys627Asn	K627N	4	Hepatocellular Carcinoma	(34)
c.1881A>C	p.Lys627Asn	K627N	4	Colon Adenocarcinoma	(12)
c.1880A>G	p.Lys627Arg	K627R	4	Hepatocellular Carcinoma	(62)
c.1880A>C	p.Lys627Thr	K627T	4	Uterine Endometrioid Carcinoma	(19-24,26)
c.1907C>A	p.Pro636Gln	P636Q	1	Lung Squamous Cell Carcinoma	(19-24,26)
c.1918G>A	p.Glu640Lys	E640K	3	Cutaneous Melanoma	(77)
c.1918G>A	p.Glu640Lys	E640K	3	Cutaneous Melanoma	(63)
c.1918G>A	p.Glu640Lys	E640K	3	Breast Invasive Ductal Carcinoma	(12)
c.1921G>C	p.Glu641Gln	E641Q	1	Esophageal Squamous Cell Carcinoma	(78)
c.1935T>G	p.Asp645Glu	D645E	1	Lung Adenocarcinoma	(12)
c.1936G>A	p.Asp646Asn	D646N	2	Lung Adenocarcinoma	(14)
c.1936G>A	p.Asp646Asn	D646N	2	Lung Adenocarcinoma	(12)
c.1939C>T	p.Leu647Phe	L647F	2	Colon Adenocarcinoma	(64)
c.1939C>G	p.Leu647Val	L647V	2	Lung Adenocarcinoma	(12)
c.1952T>C	p.Ile651Thr	I651T	2	Rectal Adenocarcinoma	(12)
c.1952T>C	p.Ile651Thr	I651T	2	Colon Adenocarcinoma	(12)
c.1958G>T	p.Arg653Ile	R653I	1	Colon Adenocarcinoma	(12)
c.1968A>G	p.Ile656Met	I656M	1	Uterine Endometrioid Carcinoma	(14)
c.1980T>G	p.Asn660Lys	N660K	2	Rectal Adenocarcinoma	(12)
c.1978A>T	p.Asn660Tyr	N660Y	2	Intrahepatic Cholangiocarcinoma	(69)
c.1983A>C	p.Lys661Asn	K661N	1	Breast Invasive Ductal Carcinoma	(14)
c.1985C>A	p.Thr662Lys	T662K	2	Neuroblastoma	(14)
c.1984A>C	p.Thr662Pro	T662P	2	Colon Adenocarcinoma	(12)
c.1989G>T	p.Lys663Asn	K663N	2	Colon Adenocarcinoma	(64)
c.1987A>C	p.Lys663Gln	K663Q	2	Colon Adenocarcinoma	(12)
c.2005G>A	p.Asp669Asn	D669N	1	Stomach Adenocarcinoma	(19-24,26)
c.2013A>C	p.Leu671Phe	L671F	1	Prostate Adenocarcinoma	(43)
c.2017A>C	p.Met673Leu	M673L	2	Pancreatic Adenocarcinoma	(79)

c.2017A>C	p.Met673Leu	M673L	2	Pancreatic Adenocarcinoma	(34)
c.2020C>T	p.Arg674Cys	R674C	7	Prostate Adenocarcinoma	(19-24,26)
c.2020C>T	p.Arg674Cys	R674C	7	Colon Adenocarcinoma	(12)
c.2021G>A	p.Arg674His	R674H	7	Colon Adenocarcinoma	(14)
c.2021G>A	p.Arg674His	R674H	7	Colon Adenocarcinoma	(12)
c.2021G>A	p.Arg674His	R674H	7	Non-Small Cell Lung Cancer	(12)
c.2021G>A	p.Arg674His	R674H	7	Intestinal Type Stomach Adenocarcinoma	(19-24,26)
c.2021G>C	p.Arg674Pro	R674P	7	Uterine Serous Carcinoma/Uterine Papillary Serous Carcinoma	(19-24,26)
c.2026C>A	p.Gln676Lys	Q676K	1	Lung Adenocarcinoma	(63)
c.2036G>A	p.Arg679Gln	R679Q	2	Colon Adenocarcinoma	(12)
c.2036G>A	p.Arg679Gln	R679Q	2	Uterine Endometrioid Carcinoma	(19-24,26)
c.2039T>C	p.Leu680Ser	L680S	1	Uterine Serous Carcinoma/Uterine Papillary Serous Carcinoma	(19-24,26)
c.2054C>G	p.Ala685Gly	A685G	2	Uterine Endometrioid Carcinoma	(34)
c.2054C>G	p.Ala685Gly	A685G	2	Uterine Endometrioid Carcinoma	(19-24,26)
c.2056A>G	p.Thr686Ala	T686A	1	Rectal Adenocarcinoma	(12)
c.2065T>A	p.Trp689Arg	W689R	1	Lung Carcinoma	(80)
c.2069T>G	p.Phe690Cys	F690C	2	Breast Invasive Lobular Carcinoma	(14)
c.2069T>G	p.Phe690Cys	F690C	2	Breast Invasive Lobular Carcinoma	(12)
c.2071C>G	p.Leu691Val	L691V	1	Diffuse Type Stomach Adenocarcinoma	(19-24,26)
c.2074C>G	p.Leu692Val	L692V	1	Bladder Urothelial Carcinoma	(19-24)
c.2088A>G	p.Ile696Met	I696M	3	Endometrial Carcinoma	(31)
c.2087T>C	p.Ile696Thr	I696T	3	Colon Adenocarcinoma	(12)
c.2086A>G	p.Ile696Val	I696V	3	Head and Neck Squamous Cell Carcinoma	(19-24)
c.2098G>T	p.Gly700Cys	G700C	4	Cutaneous Melanoma	(19-24)
c.2098G>T	p.Gly700Cys	G700C	4	Serous Ovarian Cancer	(19-24)
c.2098G>T	p.Gly700Cys	G700C	4	Serous Ovarian Cancer	(34)
c.2098G>T	p.Gly700Cys	G700C	4	Uterine Serous Carcinoma/Uterine Papillary Serous Carcinoma	(19-24,26)
c.2101A>G	p.Ile701Val	I701V	1	Endometrial Carcinoma	(31)
c.2114C>G	p.Ser705Cys	S705C	2	Lung Adenocarcinoma	(81)
c.2113T>C	p.Ser705Pro	S705P	2	Pancreatic Adenocarcinoma	(19-24)
c.2119C>T	p.Arg707Cys	R707C	9	Colon Adenocarcinoma	(12)
c.2119C>T	p.Arg707Cys	R707C	9	Colon Adenocarcinoma	(12)
c.2119C>T	p.Arg707Cys	R707C	9	Colorectal Adenocarcinoma	(12)
c.2120G>A	p.Arg707His	R707H	9	Colon Adenocarcinoma	(82)
c.2120G>A	p.Arg707His	R707H	9	Upper Tract Urothelial Carcinoma	(14)
c.2120G>A	p.Arg707His	R707H	9	Colorectal Adenocarcinoma	(15)
c.2120G>A	p.Arg707His	R707H	9	Colorectal Adenocarcinoma	(15)
c.2120G>T	p.Arg707Leu	R707L	9	Non-Small Cell Lung Cancer	(12)
c.2120G>C	p.Arg707Pro	R707P	9	Uterine Serous Carcinoma/Uterine Papillary Serous Carcinoma	(19-24,26)
c.2126G>A	p.Arg709Gln	R709Q	2	Cholangiocarcinoma	(27)
c.2126G>A	p.Arg709Gln	R709Q	2	Colorectal Adenocarcinoma	(15)
c.2131C>G	p.Arg711Gly	R711G	8	Endometrial Carcinoma	(31)
c.2131C>G	p.Arg711Gly	R711G	8	Endometrial Carcinoma	(35)
c.2132G>T	p.Arg711Leu	R711L	8	Non-Small Cell Lung Cancer	(12)
c.2132G>A	p.Arg711Gln	R711Q	8	Endometrial Carcinoma	(48)
c.2132G>A	p.Arg711Gln	R711Q	8	Colon Adenocarcinoma	(64)
c.2132G>A	p.Arg711Gln	R711Q	8	Colon Adenocarcinoma	(64)
c.2132G>A	p.Arg711Gln	R711Q	8	Colon Adenocarcinoma	(12)
c.2132G>A	p.Arg711Gln	R711Q	8	Colorectal Adenocarcinoma	(15)
c.2135A>G	p.Tyr712Cys	Y712C	3	Uterine Carcinosarcoma/Uterine Malignant Mixed Mullerian Tumor	(30)
c.2134T>G	p.Tyr712Asp	Y712D	3	Stomach Adenocarcinoma	(83)

c.2134T>G	p.Tyr712Asp	Y712D	3	Stomach Adenocarcinoma	(84)
c.2138C>A	p.Ser713Tyr	S713Y	1	Cutaneous Melanoma	(19-24)
c.2141T>C	p.Met714Thr	M714T	1	Hepatocellular Carcinoma	(62)
c.2144A>G	p.Glu715Gly	E715G	1	Non-Small Cell Lung Cancer	(12)
c.2149A>T	p.Ile717Phe	I717F	1	Rectal Adenocarcinoma	(12)
c.2154G>C	p.Met718Ile	M718I	1	Lung Squamous Cell Carcinoma	(19-24,26)
c.2155C>A	p.Pro719Thr	P719T	1	Colon Adenocarcinoma	(12)
c.2165A>C	p.Glu722Ala	E722A	1	Hepatocellular Carcinoma	(19-24,26)
c.2168A>G	p.Tyr723Cys	Y723C	1	Rectal Adenocarcinoma	(12)
c.2199G>T	p.Lys733Asn	K733N	1	Colon Adenocarcinoma	(12)
c.2225C>T	p.Ser742Leu	S742L	2	Glioblastoma Multiforme	(14)
c.2225C>T	p.Ser742Leu	S742L	2	Cutaneous Squamous Cell Carcinoma	(14)
c.2234G>A	p.Cys745Tyr	C745Y	2	Endometrial Carcinoma	(31)
c.2234G>A	p.Cys745Tyr	C745Y	2	Colon Adenocarcinoma	(12)
c.2246G>A	p.Arg749Gln	R749Q	10	Colon Adenocarcinoma	(19-24,26)
c.2246G>A	p.Arg749Gln	R749Q	10	Cancer of Unknown Primary	(14)
c.2246G>A	p.Arg749Gln	R749Q	10	Colon Adenocarcinoma	(12)
c.2246G>A	p.Arg749Gln	R749Q	10	Colon Adenocarcinoma	(12)
c.2246G>A	p.Arg749Gln	R749Q	10	Colon Adenocarcinoma	(12)
c.2246G>A	p.Arg749Gln	R749Q	10	Colon Adenocarcinoma	(12)
c.2246G>A	p.Arg749Gln	R749Q	10	Uterine Endometrioid Carcinoma	(19-24,26)
c.2246G>A	p.Arg749Gln	R749Q	10	Uterine Endometrioid Carcinoma	(19-24,26)
c.2246G>A	p.Arg749Gln	R749Q	10	Uterine Endometrioid Carcinoma	(19-24,26)
c.2246G>A	p.Arg749Gln	R749Q	10	Cervical Squamous Cell Carcinoma	(19-24,26)
c.2255T>A	p.Leu752Gln	L752Q	1	Breast Invasive Ductal Carcinoma	(13)
c.2264T>C	p.Ile755Thr	I755T	1	Colon Adenocarcinoma	(19-24,26)
c.2266C>A	p.Leu756Ile	L756I	1	Colorectal Adenocarcinoma	(54)
c.2274G>T	p.Arg758Ser	R758S	2	Lung Adenocarcinoma	(12)
c.2274G>T	p.Arg758Ser	R758S	2	Rectal Adenocarcinoma	(12)
c.2281C>T	p.Leu761Phe	L761F	2	Uterine Endometrioid Carcinoma	(19-24,26)
c.2282T>C	p.Leu761Pro	L761P	2	Colon Adenocarcinoma	(12)
c.2288A>G	p.Glu763Gly	E763G	8	Colon Adenocarcinoma	(14)
c.2288A>G	p.Glu763Gly	E763G	8	Colon Adenocarcinoma	(12)
c.2287G>A	p.Glu763Lys	E763K	8	Colon Adenocarcinoma	(29)
c.2287G>A	p.Glu763Lys	E763K	8	Mucinous Adenocarcinoma of the Colon and Rectum	(19-24,26)
c.2287G>A	p.Glu763Lys	E763K	8	Mucinous Adenocarcinoma of the Colon and Rectum	(34)
c.2287G>A	p.Glu763Lys	E763K	8	Lung Adenocarcinoma	(12)
c.2287G>A	p.Glu763Lys	E763K	8	Uterine Endometrioid Carcinoma	(19-24,26)
c.2287G>A	p.Glu763Lys	E763K	8	Ampullary Carcinoma	(53)
c.2293C>T	p.Leu765Phe	L765F	2	Cutaneous Melanoma	(77)
c.2293C>T	p.Leu765Phe	L765F	2	Cutaneous Melanoma	(63)
c.2300C>T	p.Ser767Leu	S767L	1	Colorectal Adenocarcinoma	(15)
c.2309T>C	p.Leu770Ser	L770S	1	Uterine Clear Cell Carcinoma	(14)
c.2323G>C	p.Asp775His	D775H	1	Breast Invasive Ductal Carcinoma	(12)
c.2331A>C	p.Glu777Asp	E777D	2	Colorectal Adenocarcinoma	(14)
c.2331A>C	p.Glu777Asp	E777D	2	Colon Adenocarcinoma	(12)
c.2344G>T	p.Asp782Tyr	D782Y	2	Colorectal Adenocarcinoma	(12)
c.2344G>T	p.Asp782Tyr	D782Y	2	Lung Squamous Cell Carcinoma	(19-24,26)
c.2348A>C	p.Glu783Ala	E783A	2	Lung Squamous Cell Carcinoma	(19-24,26)
c.2347G>A	p.Glu783Lys	E783K	2	Esophageal Adenocarcinoma	(85)
c.2350G>A	p.Ala784Thr	A784T	2	Colon Adenocarcinoma	(12)
c.2351C>T	p.Ala784Val	A784V	2	Colon Adenocarcinoma	(12)
c.2366G>T	p.Arg789Leu	R789L	9	Clear Cell Ovarian Cancer	(82)
c.2366G>T	p.Arg789Leu	R789L	9	Head and Neck Squamous Cell Carcinoma	(19-24)

c.2366G>C	p.Arg789Pro	R789P	9	Cutaneous Melanoma	(14)
c.2366G>C	p.Arg789Pro	R789P	9	Breast Invasive Ductal Carcinoma	(12)
c.2366G>C	p.Arg789Pro	R789P	9	Endometrial Carcinoma	(35)
c.2366G>A	p.Arg789Gln	R789Q	9	Lung Adenosquamous Carcinoma	(14)
c.2366G>A	p.Arg789Gln	R789Q	9	Uterine Endometrioid Carcinoma	(14)
c.2366G>A	p.Arg789Gln	R789Q	9	Colorectal Adenocarcinoma	(12)
c.2366G>A	p.Arg789Gln	R789Q	9	Head and Neck Squamous Cell Carcinoma	(19-24)
c.2372C>T	p.Thr791Ile	T791I	1	Endometrial Carcinoma	(31)
c.2374A>G	p.Thr792Ala	T792A	2	Uterine Endometrioid Carcinoma	(19-24,26)
c.2375C>T	p.Thr792Ile	T792I	2	Colon Adenocarcinoma	(12)
c.2393T>A	p.Met798Lys	M798K	2	Lung Adenocarcinoma	(12)
c.2392A>T	p.Met798Leu	M798L	2	Invasive Breast Carcinoma	(12)
c.2406G>A	p.Met802Ile	M802I	4	Lung Squamous Cell Carcinoma	(34)
c.2406G>A	p.Met802Ile	M802I	4	Lung Squamous Cell Carcinoma	(19-24,26)
c.2406G>T	p.Met802Ile	M802I	4	Uterine Endometrioid Carcinoma	(19-24,26)
c.2406G>A	p.Met802Ile	M802I	4	Cervical Squamous Cell Carcinoma	(19-24,26)
c.2411C>A	p.Ala804Asp	A804D	1	Serous Ovarian Cancer	(34)
c.2434C>T	p.His812Tyr	H812Y	2	Renal Clear Cell Carcinoma	(19-24,26)
c.2434C>T	p.His812Tyr	H812Y	2	Renal Clear Cell Carcinoma	(34)
c.2438C>A	p.Ala813Asp	A813D	1	Lung Squamous Cell Carcinoma	(12)
c.2443A>G	p.Lys815Glu	K815E	1	Bladder Urothelial Carcinoma	(72)
c.2449T>C	p.Ser817Pro	S817P	1	Prostate Adenocarcinoma	(12)
c.2455T>G	p.Leu819Val	L819V	1	Basaloid Penile Squamous Cell Carcinoma	(14)
c.2460G>T	p.Lys820Asn	K820N	1	Colorectal Adenocarcinoma	(86)
c.2466G>T	p.Met822Ile	M822I	1	Cutaneous Melanoma	(19-24)
c.2478G>T	p.Gln826His	Q826H	1	Uterine Endometrioid Carcinoma	(19-24,26)
c.2480C>T	p.Ser827Phe	S827F	2	Basaloid Penile Squamous Cell Carcinoma	(14)
c.2479T>C	p.Ser827Pro	S827P	2	Colon Adenocarcinoma	(12)
c.2492G>A	p.Ser831Asn	S831N	2	Pancreatic Adenocarcinoma	(12)
c.2492G>C	p.Ser831Thr	S831T	2	Renal Clear Cell Carcinoma	(66)
c.2512A>C	p.Asn838His	N838H	2	Breast Invasive Ductal Carcinoma	(12)
c.2514T>A	p.Asn838Lys	N838K	2	Lung Adenocarcinoma	(12)
c.2515G>A	p.Glu839Lys	E839K	2	Chromophobe Renal Cell Carcinoma	(19-24,26)
c.2515G>A	p.Glu839Lys	E839K	2	Chromophobe Renal Cell Carcinoma	(34)
c.2521G>A	p.Val841Met	V841M	1	Colon Adenocarcinoma	(12)
c.2526C>A	p.Asn842Lys	N842K	1	Hepatocellular Adenoma	(49)
c.2528C>A	p.Thr843Asn	T843N	1	Oligodendroglioma	(19-24)
c.2530A>G	p.Asn844Asp	N844D	1	Endometrial Carcinoma	(31)
c.2536A>G	p.Thr846Ala	T846A	3	Bladder Urothelial Carcinoma	(72)
c.2536A>G	p.Thr846Ala	T846A	3	Upper Tract Urothelial Carcinoma	(72)
c.2536A>G	p.Thr846Ala	T846A	3	Upper Tract Urothelial Carcinoma	(61)
c.2541C>A	p.His847Gln	H847Q	1	Colon Adenocarcinoma	(12)
c.2543T>C	p.Leu848Pro	L848P	3	Esophageal Adenocarcinoma	(19-24)
c.2543T>A	p.Leu848Gln	L848Q	3	Hepatocellular Carcinoma	(87)
c.2543T>A	p.Leu848Gln	L848Q	3	Hepatocellular Carcinoma	(62)
c.2546T>C	p.Leu849Ser	L849S	1	Papillary Thyroid Cancer	(19-24,26)
c.2549A>G	p.Asn850Ser	N850S	4	Bladder Urothelial Carcinoma	(72)
c.2549A>G	p.Asn850Ser	N850S	4	Upper Tract Urothelial Carcinoma	(72)
c.2549A>G	p.Asn850Ser	N850S	4	Upper Tract Urothelial Carcinoma	(61)
c.2549A>G	p.Asn850Ser	N850S	4	Small Cell Lung Cancer	(88)
c.2552T>C	p.Ile851Thr	I851T	1	Colon Adenocarcinoma	(12)
c.2558C>T	p.Ser853Leu	S853L	4	Lung Adenocarcinoma	(14)
c.2558C>T	p.Ser853Leu	S853L	4	Lung Adenocarcinoma	(12)
c.2558C>T	p.Ser853Leu	S853L	4	Lung Adenocarcinoma	(12)
c.2558C>T	p.Ser853Leu	S853L	4	Lung Adenocarcinoma	(12)
c.2564T>C	p.Leu855Pro	L855P	5	Lung Squamous Cell Carcinoma	(89)

c.2564T>C	p.Leu855Pro	L855P	5	Lung Squamous Cell Carcinoma	(89)
c.2564T>C	p.Leu855Pro	L855P	5	Lung Squamous Cell Carcinoma	(89)
c.2564T>C	p.Leu855Pro	L855P	5	Lung Squamous Cell Carcinoma	(89)
c.2564T>G	p.Leu855Arg	L855R	5	Colon Adenocarcinoma	(64)
c.2571G>T	p.Glu857Asp	E857D	2	Uterine Endometrioid Carcinoma	(14)
c.2571G>C	p.Glu857Asp	E857D	2	Bladder Urothelial Carcinoma	(19-24)
c.2580C>A	p.Phe860Leu	F860L	1	Colon Adenocarcinoma	(12)
c.2592A>C	p.Glu864Asp	E864D	1	Uterine Endometrioid Carcinoma	(19-24,26)
c.2603C>T	p.Pro868Leu	P868L	2	Head and Neck Squamous Cell Carcinoma	(19-24)
c.2603C>T	p.Pro868Leu	P868L	2	Head and Neck Squamous Cell Carcinoma	(19-24)
c.2610G>T	p.Leu870Phe	L870F	2	Rectal Adenocarcinoma	(12)
c.2609T>G	p.Leu870Trp	L870W	2	Breast Invasive Ductal Carcinoma	(90)
c.2612G>T	p.Arg871Ile	R871I	2	Lung Squamous Cell Carcinoma	(12)
c.2612G>A	p.Arg871Lys	R871K	2	Uterine Endometrioid Carcinoma	(19-24,26)
c.2624G>C	p.Gly875Ala	G875A	3	Papillary Thyroid Cancer	(19-24,26)
c.2624G>A	p.Gly875Glu	G875E	3	Mucinous Adenocarcinoma of the Colon and Rectum	(14)
c.2624G>A	p.Gly875Glu	G875E	3	Lung Squamous Cell Carcinoma	(19-24,26)
c.2634G>T	p.Gln878His	Q878H	1	Uterine Endometrioid Carcinoma	(19-24,26)
c.2637A>C	p.Lys879Asn	K879N	1	Colorectal Adenocarcinoma	(12)
c.2639C>A	p.Ser880Tyr	S880Y	1	Basaloid Penile Squamous Cell Carcinoma	(14)
c.2641G>C	p.Val881Leu	V881L	3	Colon Adenocarcinoma	(29)
c.2641G>C	p.Val881Leu	V881L	3	Lung Squamous Cell Carcinoma	(12)
c.2641G>C	p.Val881Leu	V881L	3	Lung Adenocarcinoma	(19-24)
c.2651A>G	p.Lys884Arg	K884R	1	Breast Invasive Ductal Carcinoma	(13)
c.2655G>T	p.Trp885Cys	W885C	1	Cancer of Unknown Primary	(14)
c.2663A>C	p.Asn888Thr	N888T	1	Uterine Endometrioid Carcinoma	(19-24,26)
c.2669_2670delinsTT	p.Thr890Ile	T890I	2	Cancer of Unknown Primary	(14)
c.2669_2670delinsTT	p.Thr890Ile	T890I	2	Cancer of Unknown Primary	(14)
c.2671A>G	p.Met891Val	M891V	1	Lung Adenocarcinoma	(91)
c.2681G>T	p.Arg894Ile	R894I	2	Colon Adenocarcinoma	(12)
c.2681G>A	p.Arg894Lys	R894K	2	Colorectal Adenocarcinoma	(86)
c.2696T>C	p.Phe899Ser	F899S	1	Rectal Adenocarcinoma	(12)
c.2703T>A	p.Phe901Leu	F901L	1	Colon Adenocarcinoma	(12)
c.2705T>C	p.Leu902Pro	L902P	3	Lung Adenocarcinoma	(14)
c.2705T>C	p.Leu902Pro	L902P	3	Lung Adenocarcinoma	(12)
c.2704C>G	p.Leu902Val	L902V	3	Serous Ovarian Cancer	(19-24)
c.2708G>A	p.Arg903Gln	R903Q	2	Cholangiocarcinoma	(27)
c.2708G>A	p.Arg903Gln	R903Q	2	Uterine Endometrioid Carcinoma	(19-24,26)
c.2711T>G	p.Leu904Arg	L904R	1	Colorectal Adenocarcinoma	(51)
c.2714T>A	p.Ile905Asn	I905N	1	Lung Squamous Cell Carcinoma	(19-24,26)
c.2718T>G	p.Cys906Trp	C906W	1	Endometrial Carcinoma	(31)
c.2719C>T	p.Pro907Ser	P907S	2	Lung Adenocarcinoma	(91)
c.2719C>T	p.Pro907Ser	P907S	2	Non-Small Cell Lung Cancer	(12)
c.2722G>A	p.Ala908Thr	A908T	2	Colon Adenocarcinoma	(12)
c.2723C>T	p.Ala908Val	A908V	2	Cutaneous Squamous Cell Carcinoma	(92)
c.2729T>G	p.Leu910Arg	L910R	2	Cutaneous Melanoma	(19-24)
c.2729T>G	p.Leu910Arg	L910R	2	Lung Adenocarcinoma	(82)
c.2738G>A	p.Arg913Gln	R913Q	4	Cutaneous Melanoma	(19-24)
c.2738G>A	p.Arg913Gln	R913Q	4	Colon Adenocarcinoma	(12)
c.2738G>A	p.Arg913Gln	R913Q	4	Mucinous Adenocarcinoma of the Colon and Rectum	(12)
c.2737C>T	p.Arg913Trp	R913W	4	Gastrointestinal Stromal Tumor	(14)
c.2742G>A	p.Met914Ile	M914I	1	Plasma Cell Myeloma	(55)

c.2744T>G	p.Phe915Cys	F915C	3	Rectal Adenocarcinoma	(19-24,26)
c.2745C>A	p.Phe915Leu	F915L	3	Serous Ovarian Cancer	(19-24)
c.2743T>C	p.Phe915Leu	F915L	3	Uterine Endometrioid Carcinoma	(19-24,26)
c.2756C>T	p.Ser919Leu	S919L	3	Lung Adenocarcinoma	(14)
c.2756C>T	p.Ser919Leu	S919L	3	Lung Adenocarcinoma	(14)
c.2756C>T	p.Ser919Leu	S919L	3	Lung Adenocarcinoma	(12)
c.2762C>T	p.Ser921Phe	S921F	4	Renal Non-Clear Cell Carcinoma	(93)
c.2762C>T	p.Ser921Phe	S921F	4	Lung Adenocarcinoma	(12)
c.2762C>A	p.Ser921Tyr	S921Y	4	Breast Invasive Carcinoma, NOS	(14)
c.2762C>A	p.Ser921Tyr	S921Y	4	Uterine Endometrioid Carcinoma	(19-24,26)
c.2764C>T	p.Pro922Ser	P922S	4	Bladder Urothelial Carcinoma	(72)
c.2764C>T	p.Pro922Ser	P922S	4	Bladder Urothelial Carcinoma	(72)
c.2764C>T	p.Pro922Ser	P922S	4	Bladder Urothelial Carcinoma	(72)
c.2764C>T	p.Pro922Ser	P922S	4	Bladder Urothelial Carcinoma	(72)
c.2773A>G	p.Ile925Val	I925V	1	Bladder Urothelial Carcinoma	(41)
c.2782A>G	p.Arg928Gly	R928G	3	Lung Squamous Cell Carcinoma	(17,18)
c.2782A>G	p.Arg928Gly	R928G	3	Lung Squamous Cell Carcinoma	(17,18)
c.2783G>T	p.Arg928Ile	R928I	3	Rectal Adenocarcinoma	(19-24,26)
c.2785A>C	p.Thr929Pro	T929P	1	Cholangiocarcinoma	(27)
c.2788C>A	p.Leu930Met	L930M	1	Colon Adenocarcinoma	(12)
c.2792T>A	p.Ile931Lys	I931K	2	Colon Adenocarcinoma	(12)
c.2791A>G	p.Ile931Val	I931V	2	Hepatocellular Adenoma	(49)
c.2797G>C	p.Val933Leu	V933L	1	Uterine Endometrioid Carcinoma	(14)
c.2801C>T	p.Ala934Val	A934V	1	Skin Adnexal Carcinoma	(14)
c.2805A>C	p.Lys935Asn	K935N	4	Colon Adenocarcinoma	(14)
c.2805A>C	p.Lys935Asn	K935N	4	Colon Adenocarcinoma	(12)
c.2804A>C	p.Lys935Thr	K935T	4	Mucinous Adenocarcinoma of the Colon and Rectum	(19-24,26)
c.2804A>C	p.Lys935Thr	K935T	4	Mucinous Adenocarcinoma of the Colon and Rectum	(34)
c.2807C>T	p.Ser936Phe	S936F	1	Cutaneous Squamous Cell Carcinoma	(92)
c.2812C>A	p.Gln938Lys	Q938K	1	Hepatocellular Adenoma	(49)
c.2821G>T	p.Ala941Ser	A941S	1	Uterine Endometrioid Carcinoma	(19-24,26)
c.2824A>C	p.Asn942His	N942H	3	Colon Adenocarcinoma	(19-24,26)
c.2824A>C	p.Asn942His	N942H	3	Colon Adenocarcinoma	(64)
c.2824A>C	p.Asn942His	N942H	3	Uterine Endometrioid Carcinoma	(19-24,26)
c.2827C>T	p.Leu943Phe	L943F	1	Lung Adenocarcinoma	(12)
c.2833G>A	p.Glu945Lys	E945K	1	Colon Adenocarcinoma	(29)
c.2839G>A	p.Gly947Arg	G947R	1	Hepatocellular Carcinoma	(87)
c.2845A>G	p.Lys949Glu	K949E	2	Small Cell Lung Cancer	(71)
c.2846A>C	p.Lys949Thr	K949T	2	Lung Squamous Cell Carcinoma	(12)
c.2848G>A	p.Glu950Lys	E950K	1	Lung Squamous Cell Carcinoma	(12)
c.2857A>G	p.Met953Val	M953V	1	Lung Adenocarcinoma	(12)
c.2860G>A	p.Glu954Lys	E954K	1	Lung Squamous Cell Carcinoma	(17,18)
c.2864G>A	p.Gly955Asp	G955D	1	Uterine Endometrioid Carcinoma	(19-24,26)
c.2872C>G	p.Pro958Ala	P958A	1	Colorectal Adenocarcinoma	(15)
c.2877C>A	p.Phe959Leu	F959L	2	Head and Neck Squamous Cell Carcinoma	(94)
c.2877C>A	p.Phe959Leu	F959L	2	B-Lymphoblastic Leukemia/Lymphoma, BCR-ABL1 Like	(95)
c.2885G>T	p.Ser962Ile	S962I	1	Prostate Adenocarcinoma	(43)
c.2896C>T	p.Arg966Cys	R966C	8	Stomach Adenocarcinoma	(96)
c.2896C>T	p.Arg966Cys	R966C	8	Breast Invasive Ductal Carcinoma	(12)
c.2896C>T	p.Arg966Cys	R966C	8	Colon Adenocarcinoma	(12)
c.2896C>T	p.Arg966Cys	R966C	8	Colorectal Adenocarcinoma	(15)
c.2897G>A	p.Arg966His	R966H	8	Invasive Breast Carcinoma	(36)
c.2897G>A	p.Arg966His	R966H	8	Colon Adenocarcinoma	(12)

c.2897G>A	p.Arg966His	R966H	8	Uterine Endometrioid Carcinoma	(19-24,26)
c.2897G>A	p.Arg966His	R966H	8	Uterine Endometrioid Carcinoma	(19-24,26)
c.2901G>T	p.Met967Ile	M967I	2	Colorectal Adenocarcinoma	(45)
c.2900T>C	p.Met967Thr	M967T	2	Colon Adenocarcinoma	(12)
c.2904C>G	p.Ile968Met	I968M	1	Prostate Adenocarcinoma	(14)
c.2921T>C	p.Leu974Pro	L974P	2	Invasive Breast Carcinoma	(12)
c.2921T>G	p.Leu974Arg	L974R	2	Uterine Endometrioid Carcinoma	(19-24,26)
c.2924G>T	p.Gly975Val	G975V	1	Cutaneous Melanoma	(19-24)
c.2930T>C	p.Val977Ala	V977A	1	Colon Adenocarcinoma	(12)
c.2948C>A	p.Thr983Asn	T983N	1	Uterine Endometrioid Carcinoma	(19-24,26)
c.2960C>A	p.Ser987Tyr	S987Y	1	Colorectal Adenocarcinoma	(96)
c.2963G>T	p.Arg988Ile	R988I	1	Colon Adenocarcinoma	(12)
c.2966C>T	p.Thr989Met	T989M	2	Breast Invasive Lobular Carcinoma	(14)
c.2966C>T	p.Thr989Met	T989M	2	Melanoma	(34)
c.2971C>A	p.Leu991Met	L991M	1	Colon Adenocarcinoma	(12)
c.2975C>T	p.Ser992Phe	S992F	2	Bladder Urothelial Carcinoma	(14)
c.2974T>C	p.Ser992Pro	S992P	2	Mucinous Adenocarcinoma of the Colon and Rectum	(14)
c.2977C>T	p.Arg993Cys	R993C	4	Colorectal Adenocarcinoma	(12)
c.2977C>T	p.Arg993Cys	R993C	4	Intrahepatic Cholangiocarcinoma	(69)
c.2977C>G	p.Arg993Gly	R993G	4	Upper Tract Urothelial Carcinoma	(14)
c.2978G>A	p.Arg993His	R993H	4	Stomach Adenocarcinoma	(14)
c.2980G>A	p.Asp994Asn	D994N	1	Hepatocellular Carcinoma	(25)
c.2998G>A	p.Glu1000Lys	E1000K	1	Lung Squamous Cell Carcinoma	(12)
c.3001A>T	p.Ile1001Phe	I1001F	1	Colon Adenocarcinoma	(12)
c.3007G>A	p.Val1003Met	V1003M	2	Uterine Serous Carcinoma/Uterine Papillary Serous Carcinoma	(19-24,26)
c.3007G>A	p.Val1003Met	V1003M	2	Melanoma	(70)
c.3010G>T	p.Ala1004Ser	A1004S	3	Lung Adenocarcinoma	(12)
c.3010G>T	p.Ala1004Ser	A1004S	3	Myxofibrosarcoma	(19-24,26)
c.3011C>T	p.Ala1004Val	A1004V	3	Colon Adenocarcinoma	(19-24,26)
c.3014A>G	p.His1005Arg	H1005R	4	Glioblastoma Multiforme	(97)
c.3014A>G	p.His1005Arg	H1005R	4	Glioblastoma Multiforme	(14)
c.3013C>T	p.His1005Tyr	H1005Y	4	Invasive Breast Carcinoma	(12)
c.3013C>T	p.His1005Tyr	H1005Y	4	Lung Neuroendocrine Tumor	(12)
c.3017C>T	p.Ser1006Leu	S1006L	1	Uterine Endometrioid Carcinoma	(19-24,26)
c.3023A>G	p.Glu1008Gly	E1008G	1	Lung Adenocarcinoma	(12)
c.3026T>G	p.Leu1009Arg	L1009R	2	Adenoid Cystic Carcinoma	(68)
c.3026T>G	p.Leu1009Arg	L1009R	2	Adenoid Cystic Carcinoma	(14)
c.3029G>A	p.Arg1010Gln	R1010Q	7	Colon Adenocarcinoma	(64)
c.3029G>A	p.Arg1010Gln	R1010Q	7	Upper Tract Urothelial Carcinoma	(14)
c.3029G>A	p.Arg1010Gln	R1010Q	7	Uterine Endometrioid Carcinoma	(14)
c.3029G>A	p.Arg1010Gln	R1010Q	7	Lung Adenocarcinoma	(12)
c.3029G>A	p.Arg1010Gln	R1010Q	7	Colon Adenocarcinoma	(12)
c.3029G>A	p.Arg1010Gln	R1010Q	7	Colon Adenocarcinoma	(12)
c.3029G>A	p.Arg1010Gln	R1010Q	7	Colorectal Adenocarcinoma	(15)
c.3032C>T	p.Thr1011Met	T1011M	1	Rectal Adenocarcinoma	(12)
c.3037A>G	p.Ser1013Gly	S1013G	5	Intrahepatic Cholangiocarcinoma	(14)
c.3037A>G	p.Ser1013Gly	S1013G	5	Hepatocellular Carcinoma	(34)
c.3037A>G	p.Ser1013Gly	S1013G	5	Hepatocellular Carcinoma	(19-24,26)
c.3038G>A	p.Ser1013Asn	S1013N	5	Skin	(59)
c.3038G>C	p.Ser1013Thr	S1013T	5	Endometrial Carcinoma	(31)
c.3046C>T	p.Arg1016Cys	R1016C	9	Prostate Adenocarcinoma	(98)
c.3046C>T	p.Arg1016Cys	R1016C	9	Cutaneous Squamous Cell Carcinoma	(14)
c.3046C>T	p.Arg1016Cys	R1016C	9	Colon Adenocarcinoma	(12)
c.3046C>T	p.Arg1016Cys	R1016C	9	Medullary Carcinoma of the Colon	(12)

c.3046C>T	p.Arg1016Cys	R1016C	9	Mucinous Adenocarcinoma of the Colon and Rectum	(12)
c.3047G>A	p.Arg1016His	R1016H	9	Uterine Endometrioid Carcinoma	(14)
c.3047G>A	p.Arg1016His	R1016H	9	Rectal Adenocarcinoma	(12)
c.3047G>A	p.Arg1016His	R1016H	9	Head and Neck Squamous Cell Carcinoma	(99)
c.3047G>A	p.Arg1016His	R1016H	9	Uterine Endometrioid Carcinoma	(19-24,26)
c.3050G>T	p.Gly1017Val	G1017V	2	Breast Invasive Lobular Carcinoma	(14)
c.3050G>T	p.Gly1017Val	G1017V	2	Breast Invasive Lobular Carcinoma	(12)
c.3053C>T	p.Ala1018Val	A1018V	1	Rectal Adenocarcinoma	(12)
c.3056A>T	p.Gln1019Leu	Q1019L	1	Lung Adenocarcinoma	(12)
c.3060G>C	p.Gln1020His	Q1020H	4	Colon Adenocarcinoma	(14)
c.3060G>C	p.Gln1020His	Q1020H	4	Colon Adenocarcinoma	(12)
c.3060G>C	p.Gln1020His	Q1020H	4	Lung Squamous Cell Carcinoma	(19-24,26)
c.3058C>A	p.Gln1020Lys	Q1020K	4	Colorectal Adenocarcinoma	(15)
c.3063C>G	p.His1021Gln	H1021Q	2	Lung Adenocarcinoma	(12)
c.3061C>T	p.His1021Tyr	H1021Y	2	Cancer of Unknown Primary	(14)
c.3064G>A	p.Val1022Ile	V1022I	2	Colon Adenocarcinoma	(64)
c.3064G>A	p.Val1022Ile	V1022I	2	Uterine Endometrioid Carcinoma	(19-24,26)
c.3082G>A	p.Ala1028Thr	A1028T	1	Endometrial Carcinoma	(48)
c.3085A>G	p.Ile1029Val	I1029V	2	Anaplastic Oligoastrocytoma	(14)
c.3085A>G	p.Ile1029Val	I1029V	2	Head and Neck Squamous Cell Carcinoma	(19-24)
c.3091G>A	p.Glu1031Lys	E1031K	1	Colon Adenocarcinoma	(12)
c.3095T>C	p.Leu1032Pro	L1032P	1	Melanoma	(100)
c.3100C>G	p.Gln1034Glu	Q1034E	3	Renal Clear Cell Carcinoma	(14)
c.3101A>C	p.Gln1034Pro	Q1034P	3	Colon Adenocarcinoma	(14)
c.3101A>C	p.Gln1034Pro	Q1034P	3	Colon Adenocarcinoma	(12)
c.3106A>C	p.Lys1036Gln	K1036Q	1	Colon Adenocarcinoma	(12)
c.3109C>G	p.Gln1037Glu	Q1037E	3	Breast Invasive Ductal Carcinoma	(14)
c.3109C>G	p.Gln1037Glu	Q1037E	3	Breast Invasive Ductal Carcinoma	(12)
c.3111A>C	p.Gln1037His	Q1037H	3	Tubular Stomach Adenocarcinoma	(19-24,26)
c.3113A>G	p.Asn1038Ser	N1038S	1	Lung Adenocarcinoma	(19-24)
c.3117G>C	p.Gln1039His	Q1039H	1	Mucinous Adenocarcinoma of the Colon and Rectum	(12)
c.3137T>C	p.Val1046Ala	V1046A	1	Colon Adenocarcinoma	(64)
c.3140G>A	p.Arg1047Lys	R1047K	1	Uterine Endometrioid Carcinoma	(19-24,26)

Supplementary Table 3. Mutations observed in both vascular malformations and cancer.

Residue #	Number of Vascular Mutations	Vascular Mutation	Number of Cancer Mutations	Cancer Mutation(s)
70	1	E70Q	3	E70D/G/Q
99	4	A99V	2	A99V
156	1	G156V	1	G156C
426	8	Y426C	2	Y426C/H
451	1	N451S	1	N451S
528	4	Y528C	1	Y528H
530	6	V530D	1	V530I
707	4	R707C	9	R707C/H/L/P
763	3	E763V	8	E763G/K

Supplementary Table 4. Thermal shift assay values. ΔT_m indicates difference compared to wild type (WT) for that construct. SD indicates standard deviation. N=3.

RasGAP Construct	T_m Mean (°C)	T_m SD (°C)	ΔT_m Mean (°C)	ΔT_m SD (°C)
WT	44.35	0.68	-	0.68
R194C	42.21	0.65	-2.14	0.65
R283H	39.62	0.56	-4.73	0.56
R427Q	44.01	0.79	-0.34	0.79
Y528C	40.92	0.45	-3.43	0.45
R591C	43.55	0.93	-0.80	0.93
H604N	44.02	0.43	-0.33	0.43
T612A	44.26	0.69	-0.09	0.69
W689R	42.99	0.28	-1.36	0.28
H695L	44.81	0.37	0.46	0.37
S705F	44.61	0.35	0.26	0.35
R707H	41.15	0.31	-3.20	0.31

Supplementary Table 5. Isothermal titration calorimetry analyses for RasGAP interactions with phosphorylated p190RhoGAP. Synthesized p190RhoGAP peptide (residues 1083-1111, phosphorylated on pY1087 and pY1105) titrated against RasGAP SH2-SH3-SH2 construct (residues 174-444). Parameters from individual replicates are shown with their corresponding panel in **Supplementary Figure 7**. Averages are shown as mean ± standard deviation (SD).

Construct	Figure S7 Panel	Protein Conc-entration in Cell (μM)	Peptide Conc-entration in Syringe (μM)	K_D (nM)	n	ΔH (kJ/mol)	ΔS (J/mol•K)	Blank Constant (μJ)	K_A (nM^{-1})	TΔS (kJ/mol)	ΔG (kJ/mol)
WT	A	4.94	36.3	6.49	1.356	-63.72	-59.96	-2.2	0.1542	-16.98	-46.74
WT	B	4.94	36.3	3.59	1.453	-61.36	-44.13	-2.9	0.2789	-13.16	-48.21
WT Average	–	–	–	5.04 ± 2.05	1.405 ± 0.069	-62.54 ± 1.67	-52.05 ± 11.19	-2.55 ± 0.49	0.2166 ± 0.0882	-15.07 ± 2.70	-47.48 ± 1.04
R194C	C	4.39	24.8	3.65	1.038	-118.3	-235.2	-3	0.2470	-70.13	-48.16
R194C	D	4.41	24.8	7.23	0.981	-86.06	-132.8	-5	0.1383	-39.60	-46.47
R194C	E	4.41	24.8	1.20	0.897	-96.4	-152.6	-5	0.8329	-45.48	-50.92
R194C Average	–	–	–	4.03 ± 3.03	0.972 ± 0.071	-100.3 ± 16.5	-173.5 ± 54.3	-4.33 ± 1.15	0.4061 ± 0.3736	-51.74 ± 16.20	-48.26 ± 2.25
R427Q	F	4.24	24.8	8.14	0.967	-104.8	-196.6	-3	0.1229	-58.62	-46.17
R427Q	G	4.24	24.8	2.08	0.932	-102.9	-178.8	-3.5	0.4808	-53.30	-49.56
R427Q Average	–	–	–	5.11 ± 4.29	0.950 ± 0.025	-103.9 ± 1.3	-187.7 ± 12.6	-3.25 ± 0.35	0.3019 ± 0.2531	-55.96 ± 3.76	-47.87 ± 2.40

Supplementary Table 6. Michaelis-Menten kinetics assessment of RasGAP and disease associated RasGAP mutants. Mean and standard deviation (SD) indicated for each sample. N=5.

**RasGAP Construct**	**k_{cat} Mean (s^{-1})**	**k_{cat} SD (s^{-1})**	**K_M Mean (μM)**	**K_M SD (μM)**	**k_{cat}/K_M Mean ($M^{-1}s^{-1}$)**	**k_{cat}/K_M SD ($M^{-1}s^{-1}$)**
WT	19.0	5.0	46.2	10.3	425,520	121,760
R194C	16.9	6.5	186.3	128.7	136,734	108,155
R283H	5.0	0.9	99.1	40.1	57,470	23,165
R427Q	13.9	3.1	49.6	17.1	291,720	73,738
Y528C	12.2	1.8	51.7	6.8	235,880	28,978
R591C	9.4	2.6	116.2	26.7	82,816	19,636
H604N	11.8	3.1	143.4	28.0	81,800	9,447
T612A	19.0	4.7	58.5	9.7	334,440	108,161
W689R	15.5	5.4	102.1	52.3	169,860	49,463
H695L	27.9	11.4	65.7	29.7	429,200	45,196
S705F	15.1	3.6	125.9	60.3	130,698	32,769
R707H	6.7	4.7	731.1	441.5	8,952	746

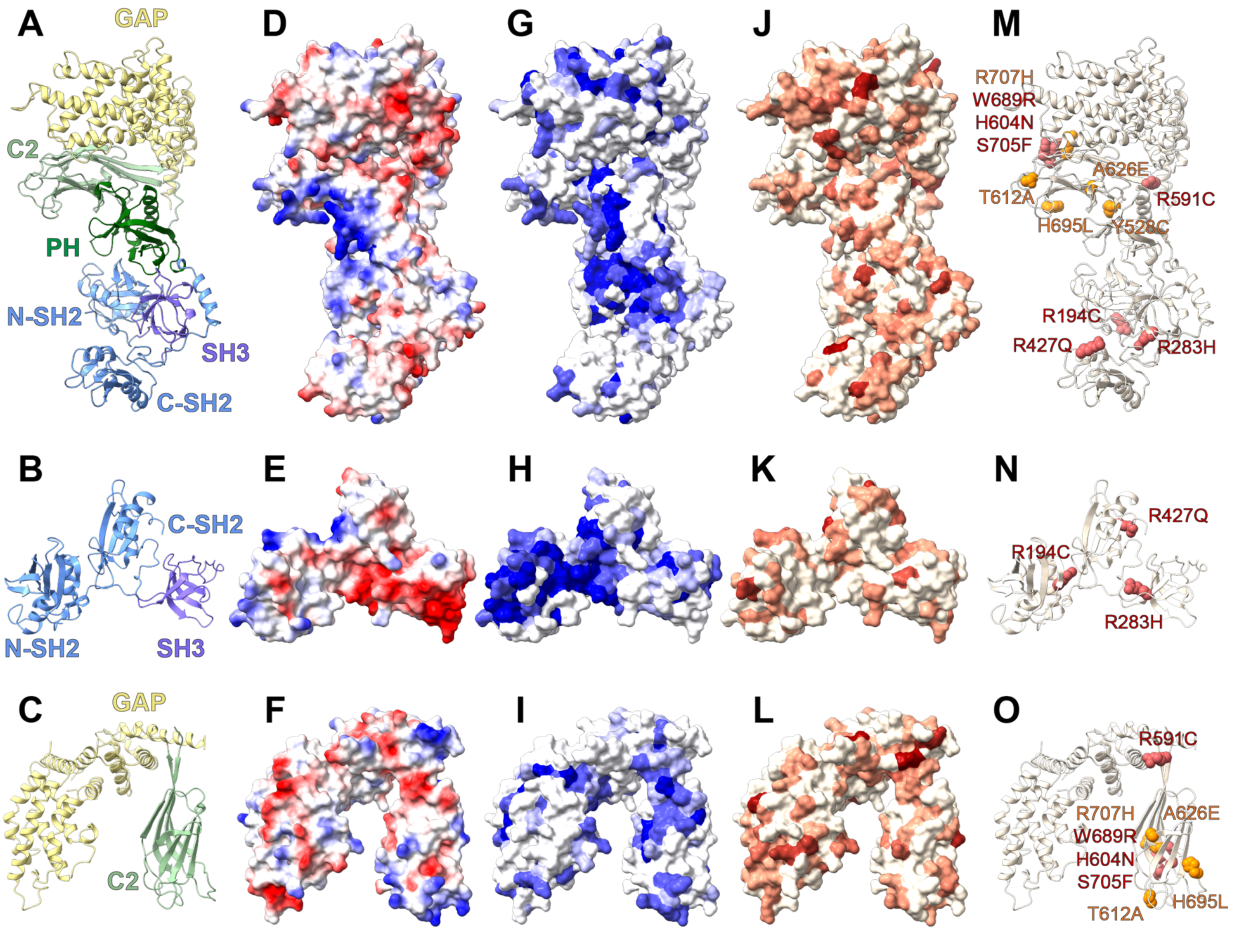

Supplementary Figure 1. Reverse views mapping *RASA1* mutations onto RasGAP. The figure depicts the AlphaFold prediction of ordered domains of RasGAP (**A**, **D**, **G**, **J**, **M**, top row) (AlphaFold ID: AF-P20936-F1-v4), crystal structure of the SH2-SH3-SH2 region (**B**, **E**, **H**, **K**, **N**, middle row) (PDB accession number: 8DGQ (101)), and crystal structure of the C2-GAP region (**C**, **F**, **I**, **L**, **O**, bottom row) (PDB accession number: 9BZ4 (102)). **A-C)** Ribbon diagrams colored by domain. **D-F)** Electrostatics of the protein surface. Positive (blue) and negative (red) charge indicated. **G-I)** Sequence conservation from low (white) to high (blue) mapped onto the protein surface. **J-L)** *RASA1* mutations mapped onto the protein surface. No reported mutations colored white, most frequent colored dark red. **M-O)** Mutations chosen for further study shown as spheres. Red indicates cancer-associated, orange indicates vascular malformations associated.

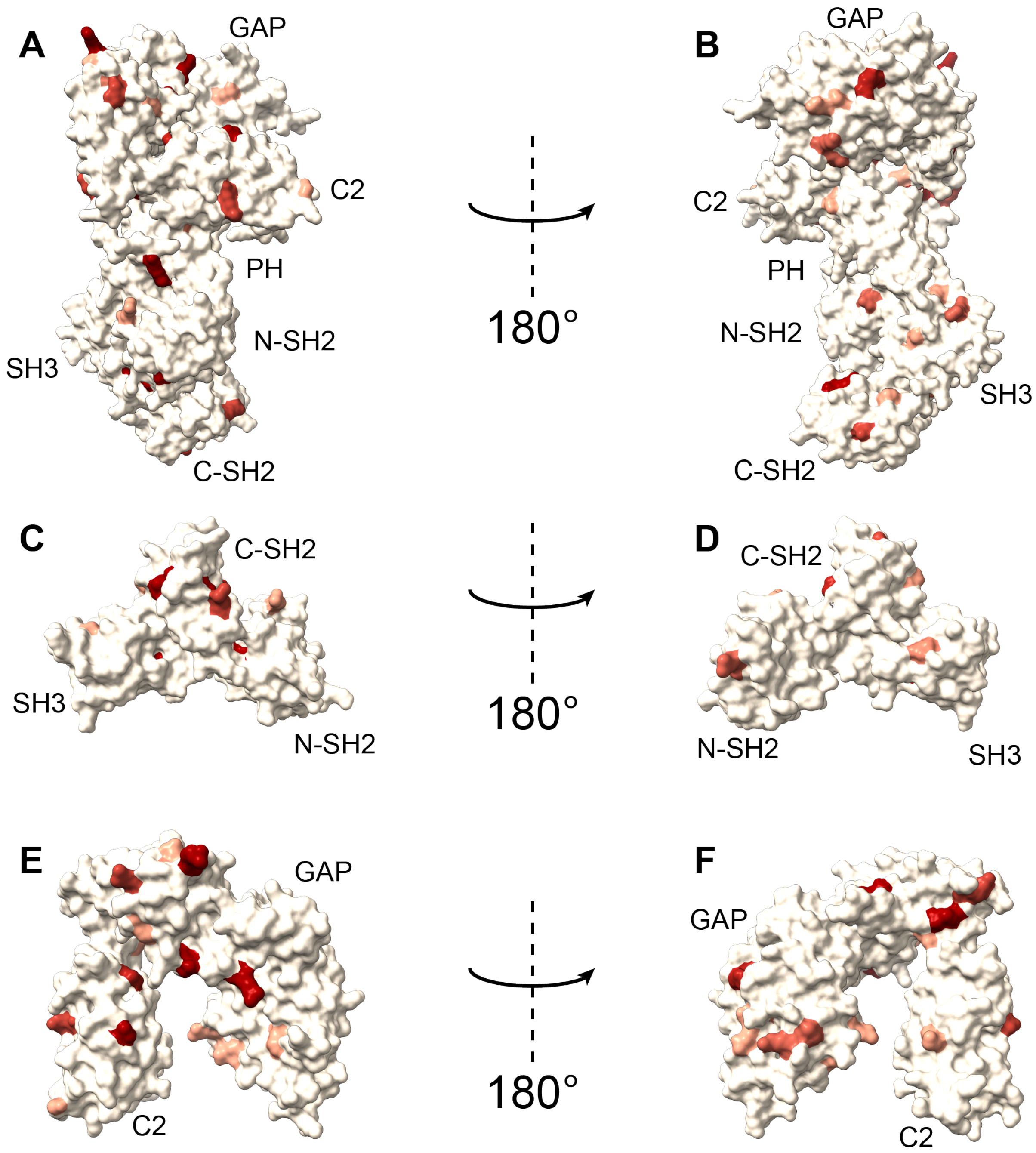

Supplementary Figure 2. Mapping hotspots of *RASA1* mutations onto RasGAP. Mutations observed more than four times depicted in red on the AlphaFold prediction of ordered domains of RasGAP (**A**, **B**, top row) (AlphaFold ID: AF-P20936-F1-v4), the crystal structure of the SH2-SH3-SH2 region (**C**, **D**, middle row) (PDB accession number: 8DGQ (101)), and the crystal structure of the C2-GAP region (**E**, **F**, bottom row) (PDB accession number: 9BZ4 (102)).

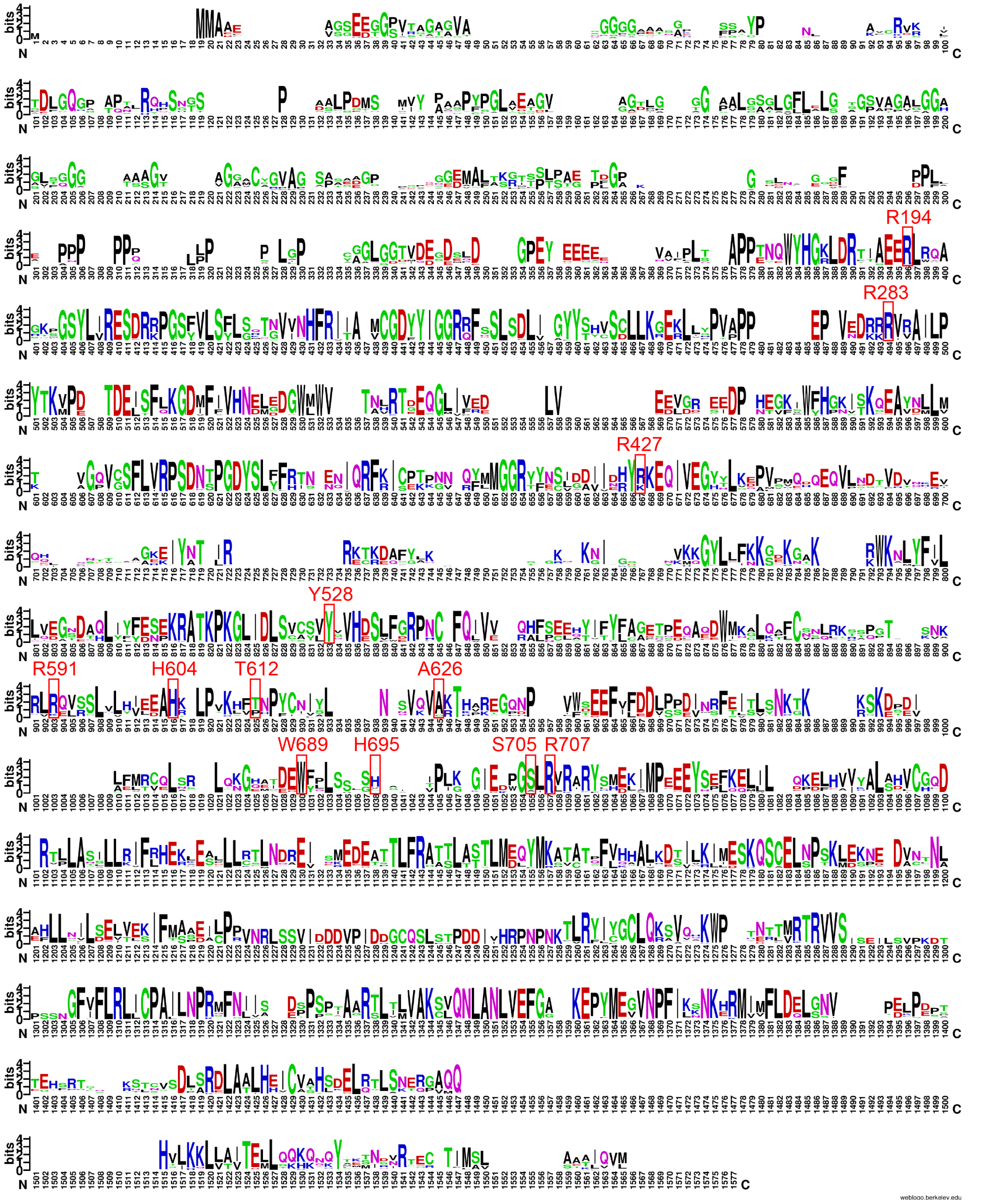

Supplementary Figure 3. Complete sequence alignment/conservation of p120RasGAP. Sequence logo based on an alignment of 209 RasGAP homologue sequences from sponges to humans. Numbering of residues is based on the overall alignment and does not correspond directly to the human p120RasGAP sequence. Height on y-axis indicates the relative frequency of each amino acid at that position. Basic residues are blue, acidic residues are red, hydrophobic residues are black, and polar residues are green or purple. Locations of mutants studied are indicated. Figure created using WebLogo (103).

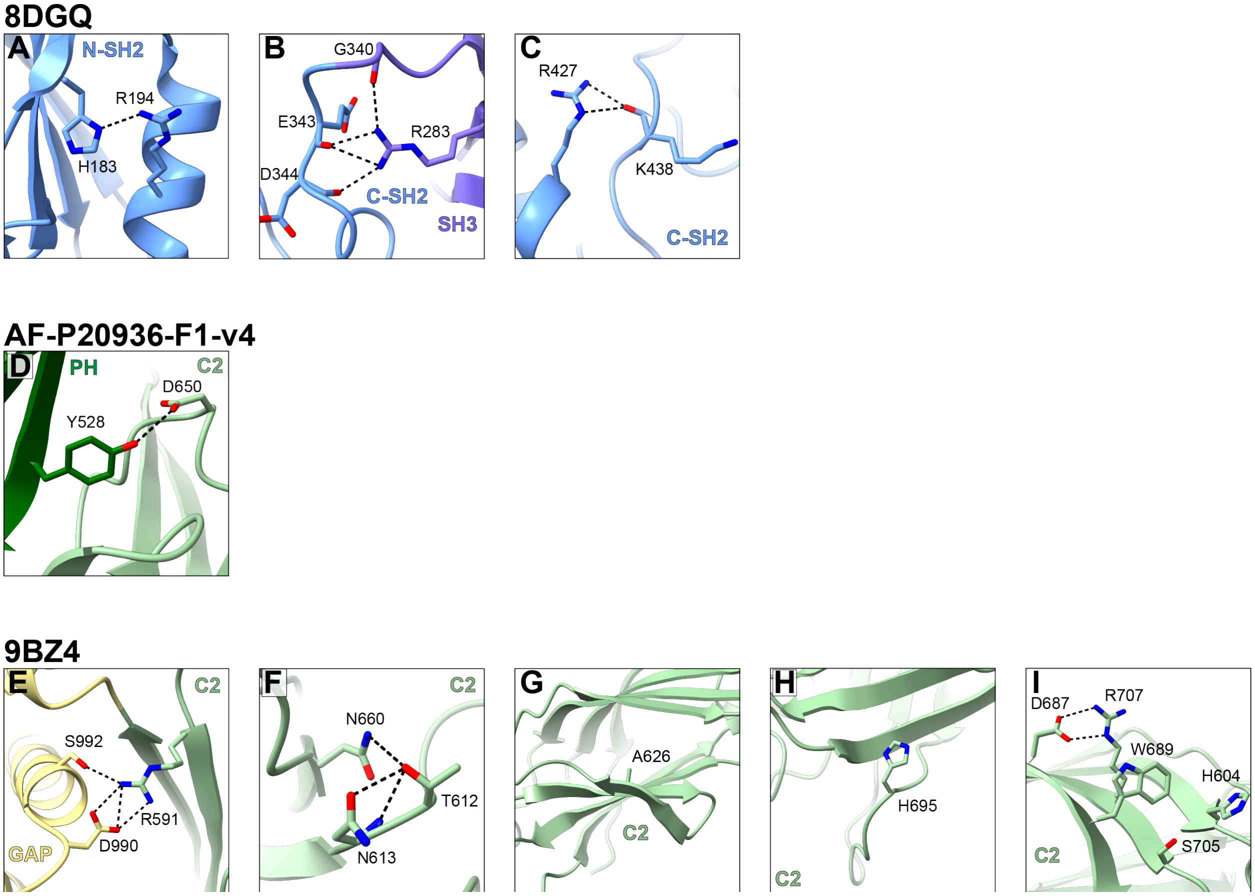

Supplementary Figure 4. Hydrogen bonding of RasGAP mutants. Hydrogen bonding of the twelve disease-associated missense mutants studied are shown as black dashed lines and engaged neighboring residues indicated. **A-C)** Crystal structure (8DGQ (101)) shown for residues R194, R283 and R427. **D)** AlphaFold structure prediction shown for residue Y528. **E-I)** Crystal structure (9BZ4 (102)) shown for residues R591, T612, A626, H695, R707, H604, W689, S705.

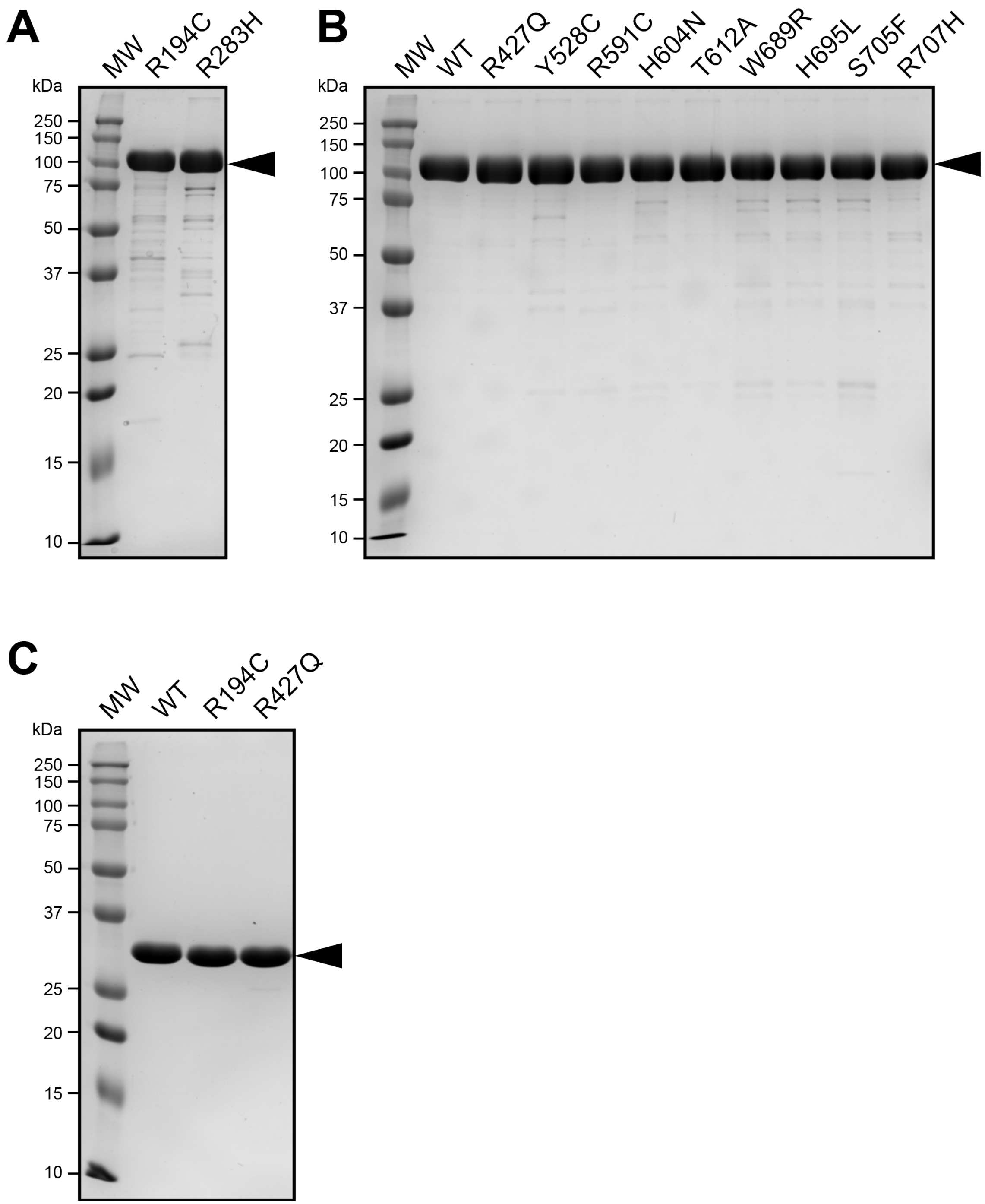

Supplementary Figure 5. Purified RasGAP constructs. Expression of near full-length RasGAP (**A-B**) and SH2-SH3-SH2 RasGAP (**C**) constructs shown by SDS-PAGE gel (Coomassie). Lane labels: MW, molecular weight; WT, wild type; point mutants indicated. Arrow indicates expected molecular weight (103.5 kDa for near full-length RasGAP, 31.5 kDa for SH2-SH3-SH2 RasGAP.

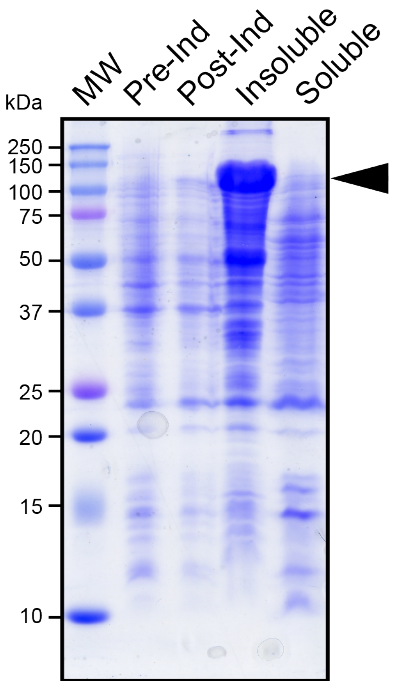

Supplementary Figure 6. Expression and solubility of RasGAP A626E mutant. Expression of p120RasGAP ΔN A626E shown by SDS-PAGE gel (Coomassie). Lane labels: MW, molecular weight; Pre-Ind, pre-induction; Post-Ind, post-induction; Insoluble, pelleted cell extract; Soluble, soluble cell extract. Arrow indicates expected molecular weight (103.5 kDa).

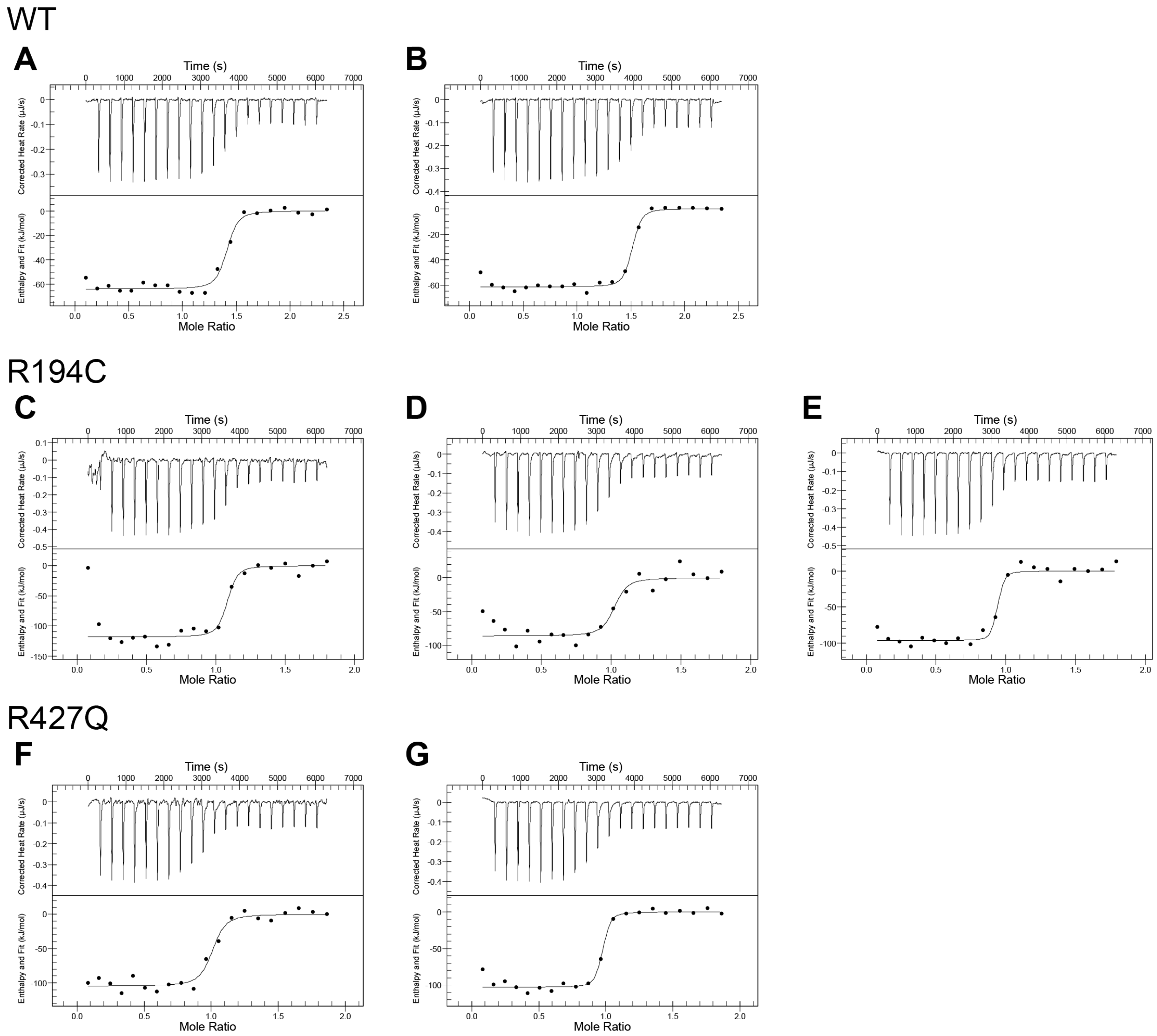

Supplementary Figure 7. Isothermal titration calorimetry traces and isotherms for RasGAP interactions with phosphorylated p190RhoGAP. Isothermal titration calorimetry traces (top) and isotherms (bottom) for individual replicates. Parameters obtained from the curve fit for each replicate and averages for each construct are presented in **Supplementary Table 5**.

References

1. Revencu, N., Boon, L. M., Mendola, A., Cordisco, M. R., Dubois, J., Clapuyt, P., Hammer, F., Amor, D. J., Irvine, A. D., Baselga, E., Dompmartin, A., Syed, S., Martin-Santiago, A., Ades, L., Collins, F., Smith, J., Sandaradura, S., Barrio, V. R., Burrows, P. E., Blei, F., Cozzolino, M., Brunetti-Pierri, N., Vicente, A., Abramowicz, M., Desir, J., Vilain, C., Chung, W. K., Wilson, A., Gardiner, C. A., Dwight, Y., Lord, D. J., Fishman, L., Cytrynbaum, C., Chamlin, S., Ghali, F., Gilaberte, Y., Joss, S., Boente Mdel, C., Leaute-Labreze, C., Delrue, M. A., Bayliss, S., Martorell, L., Gonzalez-Ensenat, M. A., Mazereeuw-Hautier, J., O'Donnell, B., Bessis, D., Pyeritz, R. E., Salhi, A., Tan, O. T., Wargon, O., Mulliken, J. B., and Vikkula, M. (2013) RASA1 mutations and associated phenotypes in 68 families with capillary malformation-arteriovenous malformation. *Hum Mutat* **34**, 1632-1641
2. Wooderchak-Donahue, W. L., Johnson, P., McDonald, J., Blei, F., Berenstein, A., Sorscher, M., Mayer, J., Scheuerle, A. E., Lewis, T., Grimmer, J. F., Richter, G. T., Steeves, M. A., Lin, A. E., Stevenson, D. A., and Bayrak-Toydemir, P. (2018) Expanding the clinical and molecular findings in RASA1 capillary malformation-arteriovenous malformation. *Eur J Hum Genet* **26**, 1521-1536
3. Maruani, A., Durieux-Verde, M., Mazereeuw-Hautier, J., Boccara, O., Martin, L., Chiaverini, C., Eschard, C., Beneton, N., Vabres, P., Balguerie, X., Plantin, P., Bessis, D., Barbarot, S., Dadban, A., Droitcourt, C., Berthelot, A., Lorette, G., Leducq, S., Samimi, M., Andres, C., Caille, A., Vourc'h, P., and Groupe de Recherche de la Societe Francaise de, D. (2018) Search for RASA1 Variants in Capillary Malformations of the Legs in 113 Children: Results from the French National Paediatric Cohort CONAPE. *Acta Derm Venereol* **98**, 251-255
4. Revencu, N., Boon, L. M., Mulliken, J. B., Enjolras, O., Cordisco, M. R., Burrows, P. E., Clapuyt, P., Hammer, F., Dubois, J., Baselga, E., Brancati, F., Carder, R., Quintal, J. M., Dallapiccola, B., Fischer, G., Frieden, I. J., Garzon, M., Harper, J., Johnson-Patel, J., Labreze, C., Martorell, L., Paltiel, H. J., Pohl, A., Prendiville, J., Quere, I., Siegel, D. H., Valente, E. M., Van Hagen, A., Van Hest, L., Vaux, K. K., Vicente, A., Weibel, L., Chitayat, D., and Vikkula, M. (2008) Parkes Weber syndrome, vein of Galen aneurysmal malformation, and other fast-flow vascular anomalies are caused by RASA1 mutations. *Hum Mutat* **29**, 959-965
5. Wooderchak-Donahue, W., Stevenson, D. A., McDonald, J., Grimmer, J. F., Gedge, F., and Bayrak-Toydemir, P. (2012) RASA1 analysis: clinical and molecular findings in a series of consecutive cases. *Eur J Med Genet* **55**, 91-95
6. Hernandez, F., Huether, R., Carter, L., Johnston, T., Thompson, J., Gossage, J. R., Chao, E., and Elliott, A. M. (2015) Mutations in RASA1 and GDF2 identified in patients with clinical features of hereditary hemorrhagic telangiectasia. *Hum Genome Var* **2**, 15040
7. Eerola, I., Boon, L. M., Mulliken, J. B., Burrows, P. E., Dompmartin, A., Watanabe, S., Vanwijck, R., and Vikkula, M. (2003) Capillary malformation-arteriovenous malformation, a new clinical and genetic disorder caused by RASA1 mutations. *Am J Hum Genet* **73**, 1240-1249
8. Smith, A. R., Carpenter, J., and Pergami, P. (2013) Nocturnal headaches and pulsatile cranial mass: the tip of an iceberg. *Pediatr Neurol* **49**, 358-360
9. Heuchan, A., Joss, S., Berg, J., Suri, M., and Bhattacharya, J. (2013) G25 RASA1 Mutations and Vein of Galen Arterial Malformations. *Archives of Disease in Childhood* **98**, A16-A17

10. Kwong, Y., Cartmill, M., Jaspan, T., and Suri, M. (2015) Fetal MRI demonstrating vein of Galen malformations in two successive pregnancies--a previously unreported occurrence. *Childs Nerv Syst* **31**, 1033-1035
11. Rizvi, H., Sanchez-Vega, F., La, K., Chatila, W., Jonsson, P., Halpenny, D., Plodkowski, A., Long, N., Sauter, J. L., Rekhtman, N., Hollmann, T., Schalper, K. A., Gainor, J. F., Shen, R., Ni, A., Arbour, K. C., Merghoub, T., Wolchok, J., Snyder, A., Chaft, J. E., Kris, M. G., Rudin, C. M., Socci, N. D., Berger, M. F., Taylor, B. S., Zehir, A., Solit, D. B., Arcila, M. E., Ladanyi, M., Riely, G. J., Schultz, N., and Hellmann, M. D. (2018) Molecular Determinants of Response to Anti-Programmed Cell Death (PD)-1 and Anti-Programmed Death-Ligand 1 (PD-L1) Blockade in Patients With Non-Small-Cell Lung Cancer Profiled With Targeted Next-Generation Sequencing. *J Clin Oncol* **36**, 633-641
12. Jee, J., Fong, C., Pichotta, K., Tran, T. N., Luthra, A., Waters, M., Fu, C., Altoe, M., Liu, S. Y., Maron, S. B., Ahmed, M., Kim, S., Pirun, M., Chatila, W. K., de Bruijn, I., Pasha, A., Kundra, R., Gross, B., Mastrogiacomo, B., Aprati, T. J., Liu, D., Gao, J., Capelletti, M., Pekala, K., Loudon, L., Perry, M., Bandlamudi, C., Donoghue, M., Satravada, B. A., Martin, A., Shen, R., Chen, Y., Brannon, A. R., Chang, J., Braunstein, L., Li, A., Safonov, A., Stonestrom, A., Sanchez-Vela, P., Wilhelm, C., Robson, M., Scher, H., Ladanyi, M., Reis-Filho, J. S., Solit, D. B., Jones, D. R., Gomez, D., Yu, H., Chakravarty, D., Yaeger, R., Abida, W., Park, W., O'Reilly, E. M., Garcia-Aguilar, J., Socci, N., Sanchez-Vega, F., Carrot-Zhang, J., Stetson, P. D., Levine, R., Rudin, C. M., Berger, M. F., Shah, S. P., Schrag, D., Razavi, P., Kehl, K. L., Li, B. T., Riely, G. J., Schultz, N., and Group, M. S. K. C. D. S. I. (2024) Automated real-world data integration improves cancer outcome prediction. *Nature* **636**, 728-736
13. Jain, E., Zañudo, J. G. T., McGillicuddy, M., Abravanel, D. L., Thomas, B. S., Kim, D., Balch, S., Navarro, J., Weiss, J. H., Hernandez, T. G., Dunphy, M., Tomson, B. N., Buendia-Buendia, J., Alao, O., Damon, A. L., Di Lascio, S., Shah, S., Small, I. K., Sosa, D., Sterlin, L., Boykin, I., Stoddard, R. E., Tsegai, N., Ulysse, U. F., Phelps, K., Frank, E., Kumari, P., Maiwald, S., Larkin, K., Pollock, S., Zimmer, A., Chastain, P. S., Cusher, T., Nguyen, C., Winnicki, S., Anastasio, E., Van Allen, E. M., Lander, E. S., Golub, T. R., Painter, C. A., and Wagle, N. (2023) The Metastatic Breast Cancer Project: leveraging patient-partnered research to expand the clinical and genomic landscape of metastatic breast cancer and accelerate discoveries. *medRxiv*, 2023.2006.2007.23291117
14. Zehir, A., Benayed, R., Shah, R. H., Syed, A., Middha, S., Kim, H. R., Srinivasan, P., Gao, J., Chakravarty, D., Devlin, S. M., Hellmann, M. D., Barron, D. A., Schram, A. M., Hameed, M., Dogan, S., Ross, D. S., Hechtman, J. F., DeLair, D. F., Yao, J., Mandelker, D. L., Cheng, D. T., Chandramohan, R., Mohanty, A. S., Ptashkin, R. N., Jayakumaran, G., Prasad, M., Syed, M. H., Rema, A. B., Liu, Z. Y., Nafa, K., Borsu, L., Sadowska, J., Casanova, J., Bacares, R., Kiecka, I. J., Razumova, A., Son, J. B., Stewart, L., Baldi, T., Mullaney, K. A., Al-Ahmadie, H., Vakiani, E., Abeshouse, A. A., Penson, A. V., Jonsson, P., Camacho, N., Chang, M. T., Won, H. H., Gross, B. E., Kundra, R., Heins, Z. J., Chen, H. W., Phillips, S., Zhang, H., Wang, J., Ochoa, A., Wills, J., Eubank, M., Thomas, S. B., Gardos, S. M., Reales, D. N., Galle, J., Durany, R., Cambria, R., Abida, W., Cercek, A., Feldman, D. R., Gounder, M. M., Hakimi, A. A., Harding, J. J., Iyer, G., Janjigian, Y. Y., Jordan, E. J., Kelly, C. M., Lowery, M. A., Morris, L. G. T., Omuro, A. M., Raj, N., Razavi, P., Shoushtari, A. N., Shukla, N., Soumerai, T. E., Varghese, A. M., Yaeger, R., Coleman, J., Bochner, B., Riely, G. J., Saltz, L. B., Scher, H. I., Sabbatini, P. J., Robson, M. E., Klimstra, D. S., Taylor, B. S., Baselga, J., Schultz, N., Hyman, D. M., Arcila, M. E., Solit, D. B., Ladanyi, M., and Berger, M. F. (2017) Mutational landscape of metastatic cancer revealed from prospective clinical sequencing of 10,000 patients. *Nat Med* **23**, 703-713

15. Giannakis, M., Mu, X. J., Shukla, S. A., Qian, Z. R., Cohen, O., Nishihara, R., Bahl, S., Cao, Y., Amin-Mansour, A., Yamauchi, M., Sukawa, Y., Stewart, C., Rosenberg, M., Mima, K., Inamura, K., Nosho, K., Nowak, J. A., Lawrence, M. S., Giovannucci, E. L., Chan, A. T., Ng, K., Meyerhardt, J. A., Van Allen, E. M., Getz, G., Gabriel, S. B., Lander, E. S., Wu, C. J., Fuchs, C. S., Ogino, S., and Garraway, L. A. (2016) Genomic Correlates of Immune-Cell Infiltrates in Colorectal Carcinoma. *Cell Rep* **17**, 1206
16. Witkiewicz, A. K., McMillan, E. A., Balaji, U., Baek, G., Lin, W. C., Mansour, J., Mollaee, M., Wagner, K. U., Koduru, P., Yopp, A., Choti, M. A., Yeo, C. J., McCue, P., White, M. A., and Knudsen, E. S. (2015) Whole-exome sequencing of pancreatic cancer defines genetic diversity and therapeutic targets. *Nat Commun* **6**, 6744
17. Abbosh, C., Birkbak, N. J., Wilson, G. A., Jamal-Hanjani, M., Constantin, T., Salari, R., Le Quesne, J., Moore, D. A., Veeriah, S., Rosenthal, R., Marafioti, T., Kirkizlar, E., Watkins, T. B. K., McGranahan, N., Ward, S., Martinson, L., Riley, J., Fraioli, F., Al Bakir, M., Gronroos, E., Zambrana, F., Endozo, R., Bi, W. L., Fennessy, F. M., Sponer, N., Johnson, D., Laycock, J., Shafi, S., Czyzewska-Khan, J., Rowan, A., Chambers, T., Matthews, N., Turajlic, S., Hiley, C., Lee, S. M., Forster, M. D., Ahmad, T., Falzon, M., Borg, E., Lawrence, D., Hayward, M., Kolvekar, S., Panagiotopoulos, N., Janes, S. M., Thakrar, R., Ahmed, A., Blackhall, F., Summers, Y., Hafez, D., Naik, A., Ganguly, A., Kareht, S., Shah, R., Joseph, L., Marie Quinn, A., Crosbie, P. A., Naidu, B., Middleton, G., Langman, G., Trotter, S., Nicolson, M., Remmen, H., Kerr, K., Chetty, M., Gomersall, L., Fennell, D. A., Nakas, A., Rathinam, S., Anand, G., Khan, S., Russell, P., Ezhil, V., Ismail, B., Irvin-Sellers, M., Prakash, V., Lester, J. F., Kornaszewska, M., Attanoos, R., Adams, H., Davies, H., Oukrif, D., Akarca, A. U., Hartley, J. A., Lowe, H. L., Lock, S., Iles, N., Bell, H., Ngai, Y., Elgar, G., Szallasi, Z., Schwarz, R. F., Herrero, J., Stewart, A., Quezada, S. A., Peggs, K. S., Van Loo, P., Dive, C., Lin, C. J., Rabinowitz, M., Aerts, H., Hackshaw, A., Shaw, J. A., Zimmermann, B. G., consortium, T. R., consortium, P., and Swanton, C. (2017) Phylogenetic ctDNA analysis depicts early-stage lung cancer evolution. *Nature* **545**, 446-451
18. Jamal-Hanjani, M., Wilson, G. A., McGranahan, N., Birkbak, N. J., Watkins, T. B. K., Veeriah, S., Shafi, S., Johnson, D. H., Mitter, R., Rosenthal, R., Salm, M., Horswell, S., Escudero, M., Matthews, N., Rowan, A., Chambers, T., Moore, D. A., Turajlic, S., Xu, H., Lee, S. M., Forster, M. D., Ahmad, T., Hiley, C. T., Abbosh, C., Falzon, M., Borg, E., Marafioti, T., Lawrence, D., Hayward, M., Kolvekar, S., Panagiotopoulos, N., Janes, S. M., Thakrar, R., Ahmed, A., Blackhall, F., Summers, Y., Shah, R., Joseph, L., Quinn, A. M., Crosbie, P. A., Naidu, B., Middleton, G., Langman, G., Trotter, S., Nicolson, M., Remmen, H., Kerr, K., Chetty, M., Gomersall, L., Fennell, D. A., Nakas, A., Rathinam, S., Anand, G., Khan, S., Russell, P., Ezhil, V., Ismail, B., Irvin-Sellers, M., Prakash, V., Lester, J. F., Kornaszewska, M., Attanoos, R., Adams, H., Davies, H., Dentro, S., Taniere, P., O'Sullivan, B., Lowe, H. L., Hartley, J. A., Iles, N., Bell, H., Ngai, Y., Shaw, J. A., Herrero, J., Szallasi, Z., Schwarz, R. F., Stewart, A., Quezada, S. A., Le Quesne, J., Van Loo, P., Dive, C., Hackshaw, A., Swanton, C., and Consortium, T. R. (2017) Tracking the Evolution of Non-Small-Cell Lung Cancer. *N Engl J Med* **376**, 2109-2121
19. Hoadley, K. A., Yau, C., Hinoue, T., Wolf, D. M., Lazar, A. J., Drill, E., Shen, R., Taylor, A. M., Cherniack, A. D., Thorsson, V., Akbani, R., Bowlby, R., Wong, C. K., Wiznerowicz, M., Sanchez-Vega, F., Robertson, A. G., Schneider, B. G., Lawrence, M. S., Noushmehr, H., Malta, T. M., Cancer Genome Atlas, N., Stuart, J. M., Benz, C. C., and Laird, P. W. (2018) Cell-of-Origin Patterns Dominate the Molecular Classification of 10,000 Tumors from 33 Types of Cancer. *Cell* **173**, 291-304 e296
20. Liu, J., Lichtenberg, T., Hoadley, K. A., Poisson, L. M., Lazar, A. J., Cherniack, A. D., Kovatich, A. J., Benz, C. C., Levine, D. A., Lee, A. V., Omberg, L., Wolf, D. M., Shriver, C. D., Thorsson, V., Cancer Genome Atlas Research, N., and Hu, H. (2018) An

Integrated TCGA Pan-Cancer Clinical Data Resource to Drive High-Quality Survival Outcome Analytics. *Cell* **173**, 400-416 e411
21. Gao, Q., Liang, W. W., Foltz, S. M., Mutharasu, G., Jayasinghe, R. G., Cao, S., Liao, W. W., Reynolds, S. M., Wyczalkowski, M. A., Yao, L., Yu, L., Sun, S. Q., Fusion Analysis Working, G., Cancer Genome Atlas Research, N., Chen, K., Lazar, A. J., Fields, R. C., Wendl, M. C., Van Tine, B. A., Vij, R., Chen, F., Nykter, M., Shmulevich, I., and Ding, L. (2018) Driver Fusions and Their Implications in the Development and Treatment of Human Cancers. *Cell Rep* **23**, 227-238 e223
22. Sanchez-Vega, F., Mina, M., Armenia, J., Chatila, W. K., Luna, A., La, K. C., Dimitriadoy, S., Liu, D. L., Kantheti, H. S., Saghafinia, S., Chakravarty, D., Daian, F., Gao, Q., Bailey, M. H., Liang, W. W., Foltz, S. M., Shmulevich, I., Ding, L., Heins, Z., Ochoa, A., Gross, B., Gao, J., Zhang, H., Kundra, R., Kandoth, C., Bahceci, I., Dervishi, L., Dogrusoz, U., Zhou, W., Shen, H., Laird, P. W., Way, G. P., Greene, C. S., Liang, H., Xiao, Y., Wang, C., Iavarone, A., Berger, A. H., Bivona, T. G., Lazar, A. J., Hammer, G. D., Giordano, T., Kwong, L. N., McArthur, G., Huang, C., Tward, A. D., Frederick, M. J., McCormick, F., Meyerson, M., Cancer Genome Atlas Research, N., Van Allen, E. M., Cherniack, A. D., Ciriello, G., Sander, C., and Schultz, N. (2018) Oncogenic Signaling Pathways in The Cancer Genome Atlas. *Cell* **173**, 321-337 e310
23. Taylor, A. M., Shih, J., Ha, G., Gao, G. F., Zhang, X., Berger, A. C., Schumacher, S. E., Wang, C., Hu, H., Liu, J., Lazar, A. J., Cancer Genome Atlas Research, N., Cherniack, A. D., Beroukhim, R., and Meyerson, M. (2018) Genomic and Functional Approaches to Understanding Cancer Aneuploidy. *Cancer Cell* **33**, 676-689 e673
24. Ellrott, K., Bailey, M. H., Saksena, G., Covington, K. R., Kandoth, C., Stewart, C., Hess, J., Ma, S., Chiotti, K. E., McLellan, M., Sofia, H. J., Hutter, C., Getz, G., Wheeler, D., Ding, L., Group, M. C. W., and Cancer Genome Atlas Research, N. (2018) Scalable Open Science Approach for Mutation Calling of Tumor Exomes Using Multiple Genomic Pipelines. *Cell Syst* **6**, 271-281 e277
25. Oberg, J. A., Glade Bender, J. L., Sulis, M. L., Pendrick, D., Sireci, A. N., Hsiao, S. J., Turk, A. T., Dela Cruz, F. S., Hibshoosh, H., Remotti, H., Zylber, R. J., Pang, J., Diolaiti, D., Koval, C., Andrews, S. J., Garvin, J. H., Yamashiro, D. J., Chung, W. K., Emerson, S. G., Nagy, P. L., Mansukhani, M. M., and Kung, A. L. (2016) Implementation of next generation sequencing into pediatric hematology-oncology practice: moving beyond actionable alterations. *Genome Med* **8**, 133
26. Bhandari, V., Hoey, C., Liu, L. Y., Lalonde, E., Ray, J., Livingstone, J., Lesurf, R., Shiah, Y. J., Vujcic, T., Huang, X., Espiritu, S. M. G., Heisler, L. E., Yousif, F., Huang, V., Yamaguchi, T. N., Yao, C. Q., Sabelnykova, V. Y., Fraser, M., Chua, M. L. K., van der Kwast, T., Liu, S. K., Boutros, P. C., and Bristow, R. G. (2019) Molecular landmarks of tumor hypoxia across cancer types. *Nat Genet* **51**, 308-318
27. Jusakul, A., Cutcutache, I., Yong, C. H., Lim, J. Q., Huang, M. N., Padmanabhan, N., Nellore, V., Kongpetch, S., Ng, A. W. T., Ng, L. M., Choo, S. P., Myint, S. S., Thanan, R., Nagarajan, S., Lim, W. K., Ng, C. C. Y., Boot, A., Liu, M., Ong, C. K., Rajasegaran, V., Lie, S., Lim, A. S. T., Lim, T. H., Tan, J., Loh, J. L., McPherson, J. R., Khuntikeo, N., Bhudhisawasdi, V., Yongvanit, P., Wongkham, S., Totoki, Y., Nakamura, H., Arai, Y., Yamasaki, S., Chow, P. K., Chung, A. Y. F., Ooi, L., Lim, K. H., Dima, S., Duda, D. G., Popescu, I., Broet, P., Hsieh, S. Y., Yu, M. C., Scarpa, A., Lai, J., Luo, D. X., Carvalho, A. L., Vettore, A. L., Rhee, H., Park, Y. N., Alexandrov, L. B., Gordan, R., Rozen, S. G., Shibata, T., Pairojkul, C., Teh, B. T., and Tan, P. (2017) Whole-Genome and Epigenomic Landscapes of Etiologically Distinct Subtypes of Cholangiocarcinoma. *Cancer Discov* **7**, 1116-1135
28. Bonilla, X., Parmentier, L., King, B., Bezrukov, F., Kaya, G., Zoete, V., Seplyarskiy, V. B., Sharpe, H. J., McKee, T., Letourneau, A., Ribaux, P. G., Popadin, K., Basset-Seguin,

N., Ben Chaabene, R., Santoni, F. A., Andrianova, M. A., Guipponi, M., Garieri, M., Verdan, C., Grosdemange, K., Sumara, O., Eilers, M., Aifantis, I., Michielin, O., de Sauvage, F. J., Antonarakis, S. E., and Nikolaev, S. I. (2016) Genomic analysis identifies new drivers and progression pathways in skin basal cell carcinoma. *Nat Genet* **48**, 398-406

29. Vasaikar, S., Huang, C., Wang, X., Petyuk, V. A., Savage, S. R., Wen, B., Dou, Y., Zhang, Y., Shi, Z., Arshad, O. A., Gritsenko, M. A., Zimmerman, L. J., McDermott, J. E., Clauss, T. R., Moore, R. J., Zhao, R., Monroe, M. E., Wang, Y. T., Chambers, M. C., Slebos, R. J. C., Lau, K. S., Mo, Q., Ding, L., Ellis, M., Thiagarajan, M., Kinsinger, C. R., Rodriguez, H., Smith, R. D., Rodland, K. D., Liebler, D. C., Liu, T., Zhang, B., and Clinical Proteomic Tumor Analysis, C. (2019) Proteogenomic Analysis of Human Colon Cancer Reveals New Therapeutic Opportunities. *Cell* **177**, 1035-1049 e1019
30. Jones, S., Stransky, N., McCord, C. L., Cerami, E., Lagowski, J., Kelly, D., Angiuoli, S. V., Sausen, M., Kann, L., Shukla, M., Makar, R., Wood, L. D., Diaz, L. A., Jr., Lengauer, C., and Velculescu, V. E. (2014) Genomic analyses of gynaecologic carcinosarcomas reveal frequent mutations in chromatin remodelling genes. *Nat Commun* **5**, 5006
31. Manning-Geist, B. L., Liu, Y. L., Devereaux, K. A., Paula, A. D. C., Zhou, Q. C., Ma, W., Selenica, P., Ceyhan-Birsoy, O., Moukarzel, L. A., Hoang, T., Gordhandas, S., Rubinstein, M. M., Friedman, C. F., Aghajanian, C., Abu-Rustum, N. R., Stadler, Z. K., Reis-Filho, J. S., Iasonos, A., Zamarin, D., Ellenson, L. H., Lakhman, Y., Mandelker, D. L., and Weigelt, B. (2022) Microsatellite Instability-High Endometrial Cancers with MLH1 Promoter Hypermethylation Have Distinct Molecular and Clinical Profiles. *Clin Cancer Res* **28**, 4302-4311
32. Morin, R. D., Mungall, K., Pleasance, E., Mungall, A. J., Goya, R., Huff, R. D., Scott, D. W., Ding, J., Roth, A., Chiu, R., Corbett, R. D., Chan, F. C., Mendez-Lago, M., Trinh, D. L., Bolger-Munro, M., Taylor, G., Hadj Khodabakhshi, A., Ben-Neriah, S., Pon, J., Meissner, B., Woolcock, B., Farnoud, N., Rogic, S., Lim, E. L., Johnson, N. A., Shah, S., Jones, S., Steidl, C., Holt, R., Birol, I., Moore, R., Connors, J. M., Gascoyne, R. D., and Marra, M. A. (2013) Mutational and structural analysis of diffuse large B-cell lymphoma using whole-genome sequencing. *Blood* **122**, 1256-1265
33. Robinson, D. R., Wu, Y. M., Lonigro, R. J., Vats, P., Cobain, E., Everett, J., Cao, X., Rabban, E., Kumar-Sinha, C., Raymond, V., Schuetze, S., Alva, A., Siddiqui, J., Chugh, R., Worden, F., Zalupski, M. M., Innis, J., Mody, R. J., Tomlins, S. A., Lucas, D., Baker, L. H., Ramnath, N., Schott, A. F., Hayes, D. F., Vijai, J., Offit, K., Stoffel, E. M., Roberts, J. S., Smith, D. C., Kunju, L. P., Talpaz, M., Cieslik, M., and Chinnaiyan, A. M. (2017) Integrative clinical genomics of metastatic cancer. *Nature* **548**, 297-303
34. Consortium, I. T. P.-C. A. o. W. G. (2020) Pan-cancer analysis of whole genomes. *Nature* **578**, 82-93
35. Ashley, C. W., Selenica, P., Patel, J., Wu, M., Nincevic, J., Lakhman, Y., Zhou, Q., Shah, R. H., Berger, M. F., Da Cruz Paula, A., Brown, D. N., Marra, A., Iasonos, A., Momeni-Boroujeni, A., Alektiar, K. M., Long Roche, K., Zivanovic, O., Mueller, J. J., Zamarin, D., Broach, V. A., Sonoda, Y., Leitao, M. M., Friedman, C. F., Jewell, E., Reis-Filho, J. S., Ellenson, L. H., Aghajanian, C., Abu-Rustum, N. R., Cadoo, K., and Weigelt, B. (2023) High-Sensitivity Mutation Analysis of Cell-Free DNA for Disease Monitoring in Endometrial Cancer. *Clin Cancer Res* **29**, 410-421
36. Nixon, M. J., Formisano, L., Mayer, I. A., Estrada, M. V., Gonzalez-Ericsson, P. I., Isakoff, S. J., Forero-Torres, A., Won, H., Sanders, M. E., Solit, D. B., Berger, M. F., Cantley, L. C., Winer, E. P., Arteaga, C. L., and Balko, J. M. (2019) PIK3CA and MAP3K1 alterations imply luminal A status and are associated with clinical benefit from pan-PI3K inhibitor buparlisib and letrozole in ER+ metastatic breast cancer. *NPJ Breast Cancer* **5**, 31

37. Hodis, E., Watson, I. R., Kryukov, G. V., Arold, S. T., Imielinski, M., Theurillat, J. P., Nickerson, E., Auclair, D., Li, L., Place, C., Dicara, D., Ramos, A. H., Lawrence, M. S., Cibulskis, K., Sivachenko, A., Voet, D., Saksena, G., Stransky, N., Onofrio, R. C., Winckler, W., Ardlie, K., Wagle, N., Wargo, J., Chong, K., Morton, D. L., Stemke-Hale, K., Chen, G., Noble, M., Meyerson, M., Ladbury, J. E., Davies, M. A., Gershenwald, J. E., Wagner, S. N., Hoon, D. S., Schadendorf, D., Lander, E. S., Gabriel, S. B., Getz, G., Garraway, L. A., and Chin, L. (2012) A landscape of driver mutations in melanoma. *Cell* **150**, 251-263

38. Gardner, E. E., Lok, B. H., Schneeberger, V. E., Desmeules, P., Miles, L. A., Arnold, P. K., Ni, A., Khodos, I., de Stanchina, E., Nguyen, T., Sage, J., Campbell, J. E., Ribich, S., Rekhtman, N., Dowlati, A., Massion, P. P., Rudin, C. M., and Poirier, J. T. (2017) Chemosensitive Relapse in Small Cell Lung Cancer Proceeds through an EZH2-SLFN11 Axis. *Cancer Cell* **31**, 286-299

39. Hugo, W., Zaretsky, J. M., Sun, L., Song, C., Moreno, B. H., Hu-Lieskovan, S., Berent-Maoz, B., Pang, J., Chmielowski, B., Cherry, G., Seja, E., Lomeli, S., Kong, X., Kelley, M. C., Sosman, J. A., Johnson, D. B., Ribas, A., and Lo, R. S. (2016) Genomic and Transcriptomic Features of Response to Anti-PD-1 Therapy in Metastatic Melanoma. *Cell* **165**, 35-44

40. Faltas, B. M., Prandi, D., Tagawa, S. T., Molina, A. M., Nanus, D. M., Sternberg, C., Rosenberg, J., Mosquera, J. M., Robinson, B., Elemento, O., Sboner, A., Beltran, H., Demichelis, F., and Rubin, M. A. (2016) Clonal evolution of chemotherapy-resistant urothelial carcinoma. *Nat Genet* **48**, 1490-1499

41. Van Allen, E. M., Mouw, K. W., Kim, P., Iyer, G., Wagle, N., Al-Ahmadie, H., Zhu, C., Ostrovnaya, I., Kryukov, G. V., O'Connor, K. W., Sfakianos, J., Garcia-Grossman, I., Kim, J., Guancial, E. A., Bambury, R., Bahl, S., Gupta, N., Farlow, D., Qu, A., Signoretti, S., Barletta, J. A., Reuter, V., Boehm, J., Lawrence, M., Getz, G., Kantoff, P., Bochner, B. H., Choueiri, T. K., Bajorin, D. F., Solit, D. B., Gabriel, S., D'Andrea, A., Garraway, L. A., and Rosenberg, J. E. (2014) Somatic ERCC2 mutations correlate with cisplatin sensitivity in muscle-invasive urothelial carcinoma. *Cancer Discov* **4**, 1140-1153

42. Chen, K., Yang, D., Li, X., Sun, B., Song, F., Cao, W., Brat, D. J., Gao, Z., Li, H., Liang, H., Zhao, Y., Zheng, H., Li, M., Buckner, J., Patterson, S. D., Ye, X., Reinhard, C., Bhathena, A., Joshi, D., Mischel, P. S., Croce, C. M., Wang, Y. M., Raghavakaimal, S., Li, H., Lu, X., Pan, Y., Chang, H., Ba, S., Luo, L., Cavenee, W. K., Zhang, W., and Hao, X. (2015) Mutational landscape of gastric adenocarcinoma in Chinese: implications for prognosis and therapy. *Proc Natl Acad Sci U S A* **112**, 1107-1112

43. Abida, W., Cyrta, J., Heller, G., Prandi, D., Armenia, J., Coleman, I., Cieslik, M., Benelli, M., Robinson, D., Van Allen, E. M., Sboner, A., Fedrizzi, T., Mosquera, J. M., Robinson, B. D., De Sarkar, N., Kunju, L. P., Tomlins, S., Wu, Y. M., Nava Rodrigues, D., Loda, M., Gopalan, A., Reuter, V. E., Pritchard, C. C., Mateo, J., Bianchini, D., Miranda, S., Carreira, S., Rescigno, P., Filipenko, J., Vinson, J., Montgomery, R. B., Beltran, H., Heath, E. I., Scher, H. I., Kantoff, P. W., Taplin, M. E., Schultz, N., deBono, J. S., Demichelis, F., Nelson, P. S., Rubin, M. A., Chinnaiyan, A. M., and Sawyers, C. L. (2019) Genomic correlates of clinical outcome in advanced prostate cancer. *Proc Natl Acad Sci U S A* **116**, 11428-11436

44. Xue, R., Chen, L., Zhang, C., Fujita, M., Li, R., Yan, S. M., Ong, C. K., Liao, X., Gao, Q., Sasagawa, S., Li, Y., Wang, J., Guo, H., Huang, Q. T., Zhong, Q., Tan, J., Qi, L., Gong, W., Hong, Z., Li, M., Zhao, J., Peng, T., Lu, Y., Lim, K. H. T., Boot, A., Ono, A., Chayama, K., Zhang, Z., Rozen, S. G., Teh, B. T., Wang, X. W., Nakagawa, H., Zeng, M. S., Bai, F., and Zhang, N. (2019) Genomic and Transcriptomic Profiling of Combined Hepatocellular and Intrahepatic Cholangiocarcinoma Reveals Distinct Molecular Subtypes. *Cancer Cell* **35**, 932-947 e938

45. Seshagiri, S., Stawiski, E. W., Durinck, S., Modrusan, Z., Storm, E. E., Conboy, C. B., Chaudhuri, S., Guan, Y., Janakiraman, V., Jaiswal, B. S., Guillory, J., Ha, C., Dijkgraaf, G. J., Stinson, J., Gnad, F., Huntley, M. A., Degenhardt, J. D., Haverty, P. M., Bourgon, R., Wang, W., Koeppen, H., Gentleman, R., Starr, T. K., Zhang, Z., Largaespada, D. A., Wu, T. D., and de Sauvage, F. J. (2012) Recurrent R-spondin fusions in colon cancer. *Nature* **488**, 660-664
46. Rodriguez-Calero, A., Gallon, J., Akhoundova, D., Maletti, S., Ferguson, A., Cyrta, J., Amstutz, U., Garofoli, A., Paradiso, V., Tomlins, S. A., Hewer, E., Genitsch, V., Fleischmann, A., Vassella, E., Rushing, E. J., Grobholz, R., Fischer, I., Jochum, W., Cathomas, G., Osunkoya, A. O., Bubendorf, L., Moch, H., Thalmann, G., Ng, C. K. Y., Gillessen, S., Piscuoglio, S., and Rubin, M. A. (2022) Alterations in homologous recombination repair genes in prostate cancer brain metastases. *Nat Commun* **13**, 2400
47. Krauthammer, M., Kong, Y., Ha, B. H., Evans, P., Bacchiocchi, A., McCusker, J. P., Cheng, E., Davis, M. J., Goh, G., Choi, M., Ariyan, S., Narayan, D., Dutton-Regester, K., Capatana, A., Holman, E. C., Bosenberg, M., Sznol, M., Kluger, H. M., Brash, D. E., Stern, D. F., Materin, M. A., Lo, R. S., Mane, S., Ma, S., Kidd, K. K., Hayward, N. K., Lifton, R. P., Schlessinger, J., Boggon, T. J., and Halaban, R. (2012) Exome sequencing identifies recurrent somatic RAC1 mutations in melanoma. *Nat Genet* **44**, 1006-1014
48. Dou, Y., Kawaler, E. A., Cui Zhou, D., Gritsenko, M. A., Huang, C., Blumenberg, L., Karpova, A., Petyuk, V. A., Savage, S. R., Satpathy, S., Liu, W., Wu, Y., Tsai, C. F., Wen, B., Li, Z., Cao, S., Moon, J., Shi, Z., Cornwell, M., Wyczalkowski, M. A., Chu, R. K., Vasaikar, S., Zhou, H., Gao, Q., Moore, R. J., Li, K., Sethuraman, S., Monroe, M. E., Zhao, R., Heiman, D., Krug, K., Clauser, K., Kothadia, R., Maruvka, Y., Pico, A. R., Oliphant, A. E., Hoskins, E. L., Pugh, S. L., Beecroft, S. J. I., Adams, D. W., Jarman, J. C., Kong, A., Chang, H. Y., Reva, B., Liao, Y., Rykunov, D., Colaprico, A., Chen, X. S., Czekanski, A., Jedryka, M., Matkowski, R., Wiznerowicz, M., Hiltke, T., Boja, E., Kinsinger, C. R., Mesri, M., Robles, A. I., Rodriguez, H., Mutch, D., Fuh, K., Ellis, M. J., DeLair, D., Thiagarajan, M., Mani, D. R., Getz, G., Noble, M., Nesvizhskii, A. I., Wang, P., Anderson, M. L., Levine, D. A., Smith, R. D., Payne, S. H., Ruggles, K. V., Rodland, K. D., Ding, L., Zhang, B., Liu, T., Fenyo, D., and Clinical Proteomic Tumor Analysis, C. (2020) Proteogenomic Characterization of Endometrial Carcinoma. *Cell* **180**, 729-748 e726
49. Ahn, S. M., Jang, S. J., Shim, J. H., Kim, D., Hong, S. M., Sung, C. O., Baek, D., Haq, F., Ansari, A. A., Lee, S. Y., Chun, S. M., Choi, S., Choi, H. J., Kim, J., Kim, S., Hwang, S., Lee, Y. J., Lee, J. E., Jung, W. R., Jang, H. Y., Yang, E., Sung, W. K., Lee, N. P., Mao, M., Lee, C., Zucman-Rossi, J., Yu, E., Lee, H. C., and Kong, G. (2014) Genomic portrait of resectable hepatocellular carcinomas: implications of RB1 and FGF19 aberrations for patient stratification. *Hepatology* **60**, 1972-1982
50. Barthel, F. P., Johnson, K. C., Varn, F. S., Moskalik, A. D., Tanner, G., Kocakavuk, E., Anderson, K. J., Abiola, O., Aldape, K., Alfaro, K. D., Alpar, D., Amin, S. B., Ashley, D. M., Bandopadhayay, P., Barnholtz-Sloan, J. S., Beroukhim, R., Bock, C., Brastianos, P. K., Brat, D. J., Brodbelt, A. R., Bruns, A. F., Bulsara, K. R., Chakrabarty, A., Chakravarti, A., Chuang, J. H., Claus, E. B., Cochran, E. J., Connelly, J., Costello, J. F., Finocchiaro, G., Fletcher, M. N., French, P. J., Gan, H. K., Gilbert, M. R., Gould, P. V., Grimmer, M. R., Iavarone, A., Ismail, A., Jenkinson, M. D., Khasraw, M., Kim, H., Kouwenhoven, M. C. M., LaViolette, P. S., Li, M., Lichter, P., Ligon, K. L., Lowman, A. K., Malta, T. M., Mazor, T., McDonald, K. L., Molinaro, A. M., Nam, D. H., Nayyar, N., Ng, H. K., Ngan, C. Y., Niclou, S. P., Niers, J. M., Noushmehr, H., Noorbakhsh, J., Ormond, D. R., Park, C. K., Poisson, L. M., Rabadan, R., Radlwimmer, B., Rao, G., Reifenberger, G., Sa, J. K., Schuster, M., Shaw, B. L., Short, S. C., Smitt, P. A. S., Sloan, A. E., Smits, M., Suzuki, H., Tabatabai, G., Van Meir, E. G., Watts, C., Weller, M., Wesseling, P., Westerman, B.

A., Widhalm, G., Woehrer, A., Yung, W. K. A., Zadeh, G., Huse, J. T., De Groot, J. F., Stead, L. F., Verhaak, R. G. W., and Consortium, G. (2019) Longitudinal molecular trajectories of diffuse glioma in adults. *Nature* **576**, 112-120

51. Alatise, O. I., Knapp, G. C., Sharma, A., Chatila, W. K., Arowolo, O. A., Olasehinde, O., Famurewa, O. C., Omisore, A. D., Komolafe, A. O., Olaofe, O. O., Katung, A. I., Ibikunle, D. E., Egberongbe, A. A., Olatoke, S. A., Agodirin, S. O., Adesiyun, O. A., Adeyeye, A., Kolawole, O. A., Olakanmi, A. O., Arora, K., Constable, J., Shah, R., Basunia, A., Sylvester, B., Wu, C., Weiser, M. R., Seier, K., Gonen, M., Stadler, Z. K., Kemel, Y., Vakiani, E., Berger, M. F., Chan, T. A., Solit, D. B., Shia, J., Sanchez-Vega, F., Schultz, N., Brennan, M., Smith, J. J., and Kingham, T. P. (2021) Molecular and phenotypic profiling of colorectal cancer patients in West Africa reveals biological insights. *Nat Commun* **12**, 6821
52. Satpathy, S., Krug, K., Jean Beltran, P. M., Savage, S. R., Petralia, F., Kumar-Sinha, C., Dou, Y., Reva, B., Kane, M. H., Avanessian, S. C., Vasaikar, S. V., Krek, A., Lei, J. T., Jaehnig, E. J., Omelchenko, T., Geffen, Y., Bergstrom, E. J., Stathias, V., Christianson, K. E., Heiman, D. I., Cieslik, M. P., Cao, S., Song, X., Ji, J., Liu, W., Li, K., Wen, B., Li, Y., Gumus, Z. H., Selvan, M. E., Soundararajan, R., Visal, T. H., Raso, M. G., Parra, E. R., Babur, O., Vats, P., Anand, S., Schraink, T., Cornwell, M., Rodrigues, F. M., Zhu, H., Mo, C. K., Zhang, Y., da Veiga Leprevost, F., Huang, C., Chinnaiyan, A. M., Wyczalkowski, M. A., Omenn, G. S., Newton, C. J., Schurer, S., Ruggles, K. V., Fenyo, D., Jewell, S. D., Thiagarajan, M., Mesri, M., Rodriguez, H., Mani, S. A., Udeshi, N. D., Getz, G., Suh, J., Li, Q. K., Hostetter, G., Paik, P. K., Dhanasekaran, S. M., Govindan, R., Ding, L., Robles, A. I., Clauser, K. R., Nesvizhskii, A. I., Wang, P., Carr, S. A., Zhang, B., Mani, D. R., Gillette, M. A., and Clinical Proteomic Tumor Analysis, C. (2021) A proteogenomic portrait of lung squamous cell carcinoma. *Cell* **184**, 4348-4371 e4340
53. Gingras, M. C., Covington, K. R., Chang, D. K., Donehower, L. A., Gill, A. J., Ittmann, M. M., Creighton, C. J., Johns, A. L., Shinbrot, E., Dewal, N., Fisher, W. E., Australian Pancreatic Cancer Genome, I., Pilarsky, C., Grutzmann, R., Overman, M. J., Jamieson, N. B., Van Buren, G., 2nd, Drummond, J., Walker, K., Hampton, O. A., Xi, L., Muzny, D. M., Doddapaneni, H., Lee, S. L., Bellair, M., Hu, J., Han, Y., Dinh, H. H., Dahdouli, M., Samra, J. S., Bailey, P., Waddell, N., Pearson, J. V., Harliwong, I., Wang, H., Aust, D., Oien, K. A., Hruban, R. H., Hodges, S. E., McElhany, A., Saengboonmee, C., Duthie, F. R., Grimmond, S. M., Biankin, A. V., Wheeler, D. A., and Gibbs, R. A. (2016) Ampullary Cancers Harbor ELF3 Tumor Suppressor Gene Mutations and Exhibit Frequent WNT Dysregulation. *Cell Rep* **14**, 907-919
54. Chen, B., Scurrah, C. R., McKinley, E. T., Simmons, A. J., Ramirez-Solano, M. A., Zhu, X., Markham, N. O., Heiser, C. N., Vega, P. N., Rolong, A., Kim, H., Sheng, Q., Drewes, J. L., Zhou, Y., Southard-Smith, A. N., Xu, Y., Ro, J., Jones, A. L., Revetta, F., Berry, L. D., Niitsu, H., Islam, M., Pelka, K., Hofree, M., Chen, J. H., Sarkizova, S., Ng, K., Giannakis, M., Boland, G. M., Aguirre, A. J., Anderson, A. C., Rozenblatt-Rosen, O., Regev, A., Hacohen, N., Kawasaki, K., Sato, T., Goettel, J. A., Grady, W. M., Zheng, W., Washington, M. K., Cai, Q., Sears, C. L., Goldenring, J. R., Franklin, J. L., Su, T., Huh, W. J., Vandekar, S., Roland, J. T., Liu, Q., Coffey, R. J., Shrubsole, M. J., and Lau, K. S. (2021) Differential pre-malignant programs and microenvironment chart distinct paths to malignancy in human colorectal polyps. *Cell* **184**, 6262-6280 e6226
55. Lohr, J. G., Stojanov, P., Carter, S. L., Cruz-Gordillo, P., Lawrence, M. S., Auclair, D., Sougnez, C., Knoechel, B., Gould, J., Saksena, G., Cibulskis, K., McKenna, A., Chapman, M. A., Straussman, R., Levy, J., Perkins, L. M., Keats, J. J., Schumacher, S. E., Rosenberg, M., Multiple Myeloma Research, C., Getz, G., and Golub, T. R. (2014) Widespread genetic heterogeneity in multiple myeloma: implications for targeted therapy. *Cancer Cell* **25**, 91-101

56. Tang, J., Fewings, E., Chang, D., Zeng, H., Liu, S., Jorapur, A., Belote, R. L., McNeal, A. S., Tan, T. M., Yeh, I., Arron, S. T., Judson-Torres, R. L., Bastian, B. C., and Shain, A. H. (2020) The genomic landscapes of individual melanocytes from human skin. *Nature* **586**, 600-605
57. Gerami, P., Tandukar, B., Deivendran, D., Olivares, S., Chen, L., Tang, J., Tan, T., Sharma, H., Bandari, A. K., Cruz-Pacheco, N., Chang, D., Marty, A., Olshen, A., Murad, N. F., Song, J., Lee, J., Yeh, I., and Hunter Shain, A. (2024) Molecular effects of indoor tanning. *bioRxiv*
58. Tandukar, B., Deivendran, D., Chen, L., Bahrani, N., Weier, B., Sharma, H., Cruz-Pacheco, N., Hu, M., Marks, K., Zitnay, R. G., Bandari, A. K., Nekoonam, R., Yeh, I., Judson-Torres, R., and Shain, A. H. (2025) Somatic mutations distinguish melanocyte subpopulations in human skin. *bioRxiv*
59. Tandukar, B., Deivendran, D., Chen, L., Cruz-Pacheco, N., Sharma, H., Xu, A., Bandari, A. K., Chen, D. B., George, C., Marty, A., Cho, R. J., Cheng, J., Saylor, D., Gerami, P., Arron, S. T., Bastian, B. C., and Shain, A. H. (2024) Genetic evolution of keratinocytes to cutaneous squamous cell carcinoma. *bioRxiv*
60. Le Gallo, M., Rudd, M. L., Urick, M. E., Hansen, N. F., Zhang, S., Program, N. C. S., Lozy, F., Sgroi, D. C., Vidal Bel, A., Matias-Guiu, X., Broaddus, R. R., Lu, K. H., Levine, D. A., Mutch, D. G., Goodfellow, P. J., Salvesen, H. B., Mullikin, J. C., and Bell, D. W. (2017) Somatic mutation profiles of clear cell endometrial tumors revealed by whole exome and targeted gene sequencing. *Cancer* **123**, 3261-3268
61. Kim, K., Hu, W., Audenet, F., Almassi, N., Hanrahan, A. J., Murray, K., Bagrodia, A., Wong, N., Clinton, T. N., Dason, S., Mohan, V., Jebiwott, S., Nagar, K., Gao, J., Penson, A., Hughes, C., Gordon, B., Chen, Z., Dong, Y., Watson, P. A., Alvim, R., Elzein, A., Gao, S. P., Cocco, E., Santin, A. D., Ostrovnaya, I., Hsieh, J. J., Sagi, I., Pietzak, E. J., Hakimi, A. A., Rosenberg, J. E., Iyer, G., Vargas, H. A., Scaltriti, M., Al-Ahmadie, H., Solit, D. B., and Coleman, J. A. (2020) Modeling biological and genetic diversity in upper tract urothelial carcinoma with patient derived xenografts. *Nat Commun* **11**, 1975
62. Chen, L., Zhang, C., Xue, R., Liu, M., Bai, J., Bao, J., Wang, Y., Jiang, N., Li, Z., Wang, W., Wang, R., Zheng, B., Yang, A., Hu, J., Liu, K., Shen, S., Zhang, Y., Bai, M., Wang, Y., Zhu, Y., Yang, S., Gao, Q., Gu, J., Gao, D., Wang, X. W., Nakagawa, H., Zhang, N., Wu, L., Rozen, S. G., Bai, F., and Wang, H. (2024) Deep whole-genome analysis of 494 hepatocellular carcinomas. *Nature* **627**, 586-593
63. Miao, D., Margolis, C. A., Vokes, N. I., Liu, D., Taylor-Weiner, A., Wankowicz, S. M., Adeegbe, D., Keliher, D., Schilling, B., Tracy, A., Manos, M., Chau, N. G., Hanna, G. J., Polak, P., Rodig, S. J., Signoretti, S., Sholl, L. M., Engelman, J. A., Getz, G., Janne, P. A., Haddad, R. I., Choueiri, T. K., Barbie, D. A., Haq, R., Awad, M. M., Schadendorf, D., Hodi, F. S., Bellmunt, J., Wong, K. K., Hammerman, P., and Van Allen, E. M. (2018) Genomic correlates of response to immune checkpoint blockade in microsatellite-stable solid tumors. *Nat Genet* **50**, 1271-1281
64. Roelands, J., Kuppen, P. J. K., Ahmed, E. I., Mall, R., Masoodi, T., Singh, P., Monaco, G., Raynaud, C., de Miranda, N., Ferraro, L., Carneiro-Lobo, T. C., Syed, N., Rawat, A., Awad, A., Decock, J., Mifsud, W., Miller, L. D., Sherif, S., Mohamed, M. G., Rinchai, D., Van den Eynde, M., Sayaman, R. W., Ziv, E., Bertucci, F., Petkar, M. A., Lorenz, S., Mathew, L. S., Wang, K., Murugesan, S., Chaussabel, D., Vahrmeijer, A. L., Wang, E., Ceccarelli, A., Fakhro, K. A., Zoppoli, G., Ballestrero, A., Tollenaar, R., Marincola, F. M., Galon, J., Khodor, S. A., Ceccarelli, M., Hendrickx, W., and Bedognetti, D. (2023) An integrated tumor, immune and microbiome atlas of colon cancer. *Nat Med* **29**, 1273-1286
65. Al Shihabi, A., Tebon, P. J., Nguyen, H. T. L., Chantharasamee, J., Sartini, S., Davarifar, A., Jensen, A. Y., Diaz-Infante, M., Cox, H., Gonzalez, A. E., Norris, S., Sperry, J.,

Nakashima, J., Tavanaie, N., Winata, H., Fitz-Gibbon, S. T., Yamaguchi, T. N., Jeong, J. H., Dry, S., Singh, A. S., Chmielowski, B., Crompton, J. G., Kalbasi, A. K., Eilber, F. C., Hornicek, F., Bernthal, N. M., Nelson, S. D., Boutros, P. C., Federman, N. C., Yanagawa, J., and Soragni, A. (2024) The landscape of drug sensitivity and resistance in sarcoma. *Cell Stem Cell* **31**, 1524-1542 e1524

66. Miao, D., Margolis, C. A., Gao, W., Voss, M. H., Li, W., Martini, D. J., Norton, C., Bosse, D., Wankowicz, S. M., Cullen, D., Horak, C., Wind-Rotolo, M., Tracy, A., Giannakis, M., Hodi, F. S., Drake, C. G., Ball, M. W., Allaf, M. E., Snyder, A., Hellmann, M. D., Ho, T., Motzer, R. J., Signoretti, S., Kaelin, W. G., Jr., Choueiri, T. K., and Van Allen, E. M. (2018) Genomic correlates of response to immune checkpoint therapies in clear cell renal cell carcinoma. *Science* **359**, 801-806

67. Martelotto, L. G., De Filippo, M. R., Ng, C. K., Natrajan, R., Fuhrmann, L., Cyrta, J., Piscuoglio, S., Wen, H. C., Lim, R. S., Shen, R., Schultheis, A. M., Wen, Y. H., Edelweiss, M., Mariani, O., Stenman, G., Chan, T. A., Colombo, P. E., Norton, L., Vincent-Salomon, A., Reis-Filho, J. S., and Weigelt, B. (2015) Genomic landscape of adenoid cystic carcinoma of the breast. *J Pathol* **237**, 179-189

68. Ho, A. S., Ochoa, A., Jayakumaran, G., Zehir, A., Valero Mayor, C., Tepe, J., Makarov, V., Dalin, M. G., He, J., Bailey, M., Montesion, M., Ross, J. S., Miller, V. A., Chan, L., Ganly, I., Dogan, S., Katabi, N., Tsipouras, P., Ha, P., Agrawal, N., Solit, D. B., Futreal, P. A., El Naggar, A. K., Reis-Filho, J. S., Weigelt, B., Ho, A. L., Schultz, N., Chan, T. A., and Morris, L. G. (2019) Genetic hallmarks of recurrent/metastatic adenoid cystic carcinoma. *J Clin Invest* **129**, 4276-4289

69. Zou, S., Li, J., Zhou, H., Frech, C., Jiang, X., Chu, J. S., Zhao, X., Li, Y., Li, Q., Wang, H., Hu, J., Kong, G., Wu, M., Ding, C., Chen, N., and Hu, H. (2014) Mutational landscape of intrahepatic cholangiocarcinoma. *Nat Commun* **5**, 5696

70. Van Allen, E. M., Wagle, N., Sucker, A., Treacy, D. J., Johannessen, C. M., Goetz, E. M., Place, C. S., Taylor-Weiner, A., Whittaker, S., Kryukov, G. V., Hodis, E., Rosenberg, M., McKenna, A., Cibulskis, K., Farlow, D., Zimmer, L., Hillen, U., Gutzmer, R., Goldinger, S. M., Ugurel, S., Gogas, H. J., Egberts, F., Berking, C., Trefzer, U., Loquai, C., Weide, B., Hassel, J. C., Gabriel, S. B., Carter, S. L., Getz, G., Garraway, L. A., Schadendorf, D., and Dermatologic Cooperative Oncology Group of, G. (2014) The genetic landscape of clinical resistance to RAF inhibition in metastatic melanoma. *Cancer Discov* **4**, 94-109

71. George, J., Lim, J. S., Jang, S. J., Cun, Y., Ozretic, L., Kong, G., Leenders, F., Lu, X., Fernandez-Cuesta, L., Bosco, G., Muller, C., Dahmen, I., Jahchan, N. S., Park, K. S., Yang, D., Karnezis, A. N., Vaka, D., Torres, A., Wang, M. S., Korbel, J. O., Menon, R., Chun, S. M., Kim, D., Wilkerson, M., Hayes, N., Engelmann, D., Putzer, B., Bos, M., Michels, S., Vlasic, I., Seidel, D., Pinther, B., Schaub, P., Becker, C., Altmuller, J., Yokota, J., Kohno, T., Iwakawa, R., Tsuta, K., Noguchi, M., Muley, T., Hoffmann, H., Schnabel, P. A., Petersen, I., Chen, Y., Soltermann, A., Tischler, V., Choi, C. M., Kim, Y. H., Massion, P. P., Zou, Y., Jovanovic, D., Kontic, M., Wright, G. M., Russell, P. A., Solomon, B., Koch, I., Lindner, M., Muscarella, L. A., la Torre, A., Field, J. K., Jakopovic, M., Knezevic, J., Castanos-Velez, E., Roz, L., Pastorino, U., Brustugun, O. T., Lund-Iversen, M., Thunnissen, E., Kohler, J., Schuler, M., Botling, J., Sandelin, M., Sanchez-Cespedes, M., Salvesen, H. B., Achter, V., Lang, U., Bogus, M., Schneider, P. M., Zander, T., Ansen, S., Hallek, M., Wolf, J., Vingron, M., Yatabe, Y., Travis, W. D., Nurnberg, P., Reinhardt, C., Perner, S., Heukamp, L., Buttner, R., Haas, S. A., Brambilla, E., Peifer, M., Sage, J., and Thomas, R. K. (2015) Comprehensive genomic profiles of small cell lung cancer. *Nature* **524**, 47-53

72. Lee, S. H., Hu, W., Matulay, J. T., Silva, M. V., Owczarek, T. B., Kim, K., Chua, C. W., Barlow, L. J., Kandoth, C., Williams, A. B., Bergren, S. K., Pietzak, E. J., Anderson, C.

B., Benson, M. C., Coleman, J. A., Taylor, B. S., Abate-Shen, C., McKiernan, J. M., Al-Ahmadie, H., Solit, D. B., and Shen, M. M. (2018) Tumor Evolution and Drug Response in Patient-Derived Organoid Models of Bladder Cancer. *Cell* **173**, 515-528 e517

73. Petralia, F., Tignor, N., Reva, B., Koptyra, M., Chowdhury, S., Rykunov, D., Krek, A., Ma, W., Zhu, Y., Ji, J., Calinawan, A., Whiteaker, J. R., Colaprico, A., Stathias, V., Omelchenko, T., Song, X., Raman, P., Guo, Y., Brown, M. A., Ivey, R. G., Szpyt, J., Guha Thakurta, S., Gritsenko, M. A., Weitz, K. K., Lopez, G., Kalayci, S., Gumus, Z. H., Yoo, S., da Veiga Leprevost, F., Chang, H. Y., Krug, K., Katsnelson, L., Wang, Y., Kennedy, J. J., Voytovich, U. J., Zhao, L., Gaonkar, K. S., Ennis, B. M., Zhang, B., Baubet, V., Tauhid, L., Lilly, J. V., Mason, J. L., Farrow, B., Young, N., Leary, S., Moon, J., Petyuk, V. A., Nazarian, J., Adappa, N. D., Palmer, J. N., Lober, R. M., Rivero-Hinojosa, S., Wang, L. B., Wang, J. M., Broberg, M., Chu, R. K., Moore, R. J., Monroe, M. E., Zhao, R., Smith, R. D., Zhu, J., Robles, A. I., Mesri, M., Boja, E., Hiltke, T., Rodriguez, H., Zhang, B., Schadt, E. E., Mani, D. R., Ding, L., Iavarone, A., Wiznerowicz, M., Schurer, S., Chen, X. S., Heath, A. P., Rokita, J. L., Nesvizhskii, A. I., Fenyo, D., Rodland, K. D., Liu, T., Gygi, S. P., Paulovich, A. G., Resnick, A. C., Storm, P. B., Rood, B. R., Wang, P., Children's Brain Tumor, N., and Clinical Proteomic Tumor Analysis, C. (2020) Integrated Proteogenomic Characterization across Major Histological Types of Pediatric Brain Cancer. *Cell* **183**, 1962-1985 e1931

74. Painter, C. A., Jain, E., Tomson, B. N., Dunphy, M., Stoddard, R. E., Thomas, B. S., Damon, A. L., Shah, S., Kim, D., Gomez Tejeda Zanudo, J., Hornick, J. L., Chen, Y. L., Merriam, P., Raut, C. P., Demetri, G. D., Van Tine, B. A., Lander, E. S., Golub, T. R., and Wagle, N. (2020) The Angiosarcoma Project: enabling genomic and clinical discoveries in a rare cancer through patient-partnered research. *Nat Med* **26**, 181-187

75. Gillette, M. A., Satpathy, S., Cao, S., Dhanasekaran, S. M., Vasaikar, S. V., Krug, K., Petralia, F., Li, Y., Liang, W. W., Reva, B., Krek, A., Ji, J., Song, X., Liu, W., Hong, R., Yao, L., Blumenberg, L., Savage, S. R., Wendl, M. C., Wen, B., Li, K., Tang, L. C., MacMullan, M. A., Avanessian, S. C., Kane, M. H., Newton, C. J., Cornwell, M., Kothadia, R. B., Ma, W., Yoo, S., Mannan, R., Vats, P., Kumar-Sinha, C., Kawaler, E. A., Omelchenko, T., Colaprico, A., Geffen, Y., Maruvka, Y. E., da Veiga Leprevost, F., Wiznerowicz, M., Gumus, Z. H., Veluswamy, R. R., Hostetter, G., Heiman, D. I., Wyczalkowski, M. A., Hiltke, T., Mesri, M., Kinsinger, C. R., Boja, E. S., Omenn, G. S., Chinnaiyan, A. M., Rodriguez, H., Li, Q. K., Jewell, S. D., Thiagarajan, M., Getz, G., Zhang, B., Fenyo, D., Ruggles, K. V., Cieslik, M. P., Robles, A. I., Clauser, K. R., Govindan, R., Wang, P., Nesvizhskii, A. I., Ding, L., Mani, D. R., Carr, S. A., and Clinical Proteomic Tumor Analysis, C. (2020) Proteogenomic Characterization Reveals Therapeutic Vulnerabilities in Lung Adenocarcinoma. *Cell* **182**, 200-225 e235

76. Nguyen, B., Fong, C., Luthra, A., Smith, S. A., DiNatale, R. G., Nandakumar, S., Walch, H., Chatila, W. K., Madupuri, R., Kundra, R., Bielski, C. M., Mastrogiacomo, B., Donoghue, M. T. A., Boire, A., Chandarlapaty, S., Ganesh, K., Harding, J. J., Iacobuzio-Donahue, C. A., Razavi, P., Reznik, E., Rudin, C. M., Zamarin, D., Abida, W., Abou-Alfa, G. K., Aghajanian, C., Cercek, A., Chi, P., Feldman, D., Ho, A. L., Iyer, G., Janjigian, Y. Y., Morris, M., Motzer, R. J., O'Reilly, E. M., Postow, M. A., Raj, N. P., Riely, G. J., Robson, M. E., Rosenberg, J. E., Safonov, A., Shoushtari, A. N., Tap, W., Teo, M. Y., Varghese, A. M., Voss, M., Yaeger, R., Zauderer, M. G., Abu-Rustum, N., Garcia-Aguilar, J., Bochner, B., Hakimi, A., Jarnagin, W. R., Jones, D. R., Molena, D., Morris, L., Rios-Doria, E., Russo, P., Singer, S., Strong, V. E., Chakravarty, D., Ellenson, L. H., Gopalan, A., Reis-Filho, J. S., Weigelt, B., Ladanyi, M., Gonen, M., Shah, S. P., Massague, J., Gao, J., Zehir, A., Berger, M. F., Solit, D. B., Bakhoum, S. F., Sanchez-Vega, F., and Schultz, N. (2022) Genomic characterization of metastatic patterns from prospective clinical sequencing of 25,000 patients. *Cell* **185**, 563-575 e511

77. Van Allen, E. M., Miao, D., Schilling, B., Shukla, S. A., Blank, C., Zimmer, L., Sucker, A., Hillen, U., Foppen, M. H. G., Goldinger, S. M., Utikal, J., Hassel, J. C., Weide, B., Kaehler, K. C., Loquai, C., Mohr, P., Gutzmer, R., Dummer, R., Gabriel, S., Wu, C. J., Schadendorf, D., and Garraway, L. A. (2015) Genomic correlates of response to CTLA-4 blockade in metastatic melanoma. *Science* **350**, 207-211
78. Song, Y., Li, L., Ou, Y., Gao, Z., Li, E., Li, X., Zhang, W., Wang, J., Xu, L., Zhou, Y., Ma, X., Liu, L., Zhao, Z., Huang, X., Fan, J., Dong, L., Chen, G., Ma, L., Yang, J., Chen, L., He, M., Li, M., Zhuang, X., Huang, K., Qiu, K., Yin, G., Guo, G., Feng, Q., Chen, P., Wu, Z., Wu, J., Ma, L., Zhao, J., Luo, L., Fu, M., Xu, B., Chen, B., Li, Y., Tong, T., Wang, M., Liu, Z., Lin, D., Zhang, X., Yang, H., Wang, J., and Zhan, Q. (2014) Identification of genomic alterations in oesophageal squamous cell cancer. *Nature* **509**, 91-95
79. Bailey, P., Chang, D. K., Nones, K., Johns, A. L., Patch, A. M., Gingras, M. C., Miller, D. K., Christ, A. N., Bruxner, T. J., Quinn, M. C., Nourse, C., Murtaugh, L. C., Harliwong, I., Idrisoglu, S., Manning, S., Nourbakhsh, E., Wani, S., Fink, L., Holmes, O., Chin, V., Anderson, M. J., Kazakoff, S., Leonard, C., Newell, F., Waddell, N., Wood, S., Xu, Q., Wilson, P. J., Cloonan, N., Kassahn, K. S., Taylor, D., Quek, K., Robertson, A., Pantano, L., Mincarelli, L., Sanchez, L. N., Evers, L., Wu, J., Pinese, M., Cowley, M. J., Jones, M. D., Colvin, E. K., Nagrial, A. M., Humphrey, E. S., Chantrill, L. A., Mawson, A., Humphris, J., Chou, A., Pajic, M., Scarlett, C. J., Pinho, A. V., Giry-Laterriere, M., Rooman, I., Samra, J. S., Kench, J. G., Lovell, J. A., Merrett, N. D., Toon, C. W., Epari, K., Nguyen, N. Q., Barbour, A., Zeps, N., Moran-Jones, K., Jamieson, N. B., Graham, J. S., Duthie, F., Oien, K., Hair, J., Grutzmann, R., Maitra, A., Iacobuzio-Donahue, C. A., Wolfgang, C. L., Morgan, R. A., Lawlor, R. T., Corbo, V., Bassi, C., Rusev, B., Capelli, P., Salvia, R., Tortora, G., Mukhopadhyay, D., Petersen, G. M., Australian Pancreatic Cancer Genome, I., Munzy, D. M., Fisher, W. E., Karim, S. A., Eshleman, J. R., Hruban, R. H., Pilarsky, C., Morton, J. P., Sansom, O. J., Scarpa, A., Musgrove, E. A., Bailey, U. M., Hofmann, O., Sutherland, R. L., Wheeler, D. A., Gill, A. J., Gibbs, R. A., Pearson, J. V., Waddell, N., Biankin, A. V., and Grimmond, S. M. (2016) Genomic analyses identify molecular subtypes of pancreatic cancer. *Nature* **531**, 47-52
80. Zhang, J., Bajari, R., Andric, D., Gerthoffert, F., Lepsa, A., Nahal-Bose, H., Stein, L. D., and Ferretti, V. (2019) The International Cancer Genome Consortium Data Portal. *Nat Biotechnol* **37**, 367-369
81. Zhang, T., Joubert, P., Ansari-Pour, N., Zhao, W., Hoang, P. H., Lokanga, R., Moye, A. L., Rosenbaum, J., Gonzalez-Perez, A., Martinez-Jimenez, F., Castro, A., Muscarella, L. A., Hofman, P., Consonni, D., Pesatori, A. C., Kebede, M., Li, M., Gould Rothberg, B. E., Peneva, I., Schabath, M. B., Poeta, M. L., Costantini, M., Hirsch, D., Heselmeyer-Haddad, K., Hutchinson, A., Olanich, M., Lawrence, S. M., Lenz, P., Duggan, M., Bhawsar, P. M. S., Sang, J., Kim, J., Mendoza, L., Saini, N., Klimczak, L. J., Islam, S. M. A., Otlu, B., Khandekar, A., Cole, N., Stewart, D. R., Choi, J., Brown, K. M., Caporaso, N. E., Wilson, S. H., Pommier, Y., Lan, Q., Rothman, N., Almeida, J. S., Carter, H., Ried, T., Kim, C. F., Lopez-Bigas, N., Garcia-Closas, M., Shi, J., Bosse, Y., Zhu, B., Gordenin, D. A., Alexandrov, L. B., Chanock, S. J., Wedge, D. C., and Landi, M. T. (2021) Genomic and evolutionary classification of lung cancer in never smokers. *Nat Genet* **53**, 1348-1359
82. Pleasance, E., Titmuss, E., Williamson, L., Kwan, H., Culibrk, L., Zhao, E. Y., Dixon, K., Fan, K., Bowlby, R., Jones, M. R., Shen, Y., Grewal, J. K., Ashkani, J., Wee, K., Grisdale, C. J., Thibodeau, M. L., Bozoky, Z., Pearson, H., Majounie, E., Vira, T., Shenwai, R., Mungall, K. L., Chuah, E., Davies, A., Warren, M., Reisle, C., Bonakdar, M., Taylor, G. A., Csizmok, V., Chan, S. K., Zong, Z., Bilobram, S., Muhammadzadeh, A., D'Souza, D., Corbett, R. D., MacMillan, D., Carreira, M., Choo, C., Bleile, D., Sadeghi, S., Zhang, W., Wong, T., Cheng, D., Brown, S. D., Holt, R. A., Moore, R. A.,

Mungall, A. J., Zhao, Y., Nelson, J., Fok, A., Ma, Y., Lee, M. K. C., Lavoie, J. M., Mendis, S., Karasinska, J. M., Deol, B., Fisic, A., Schaeffer, D. F., Yip, S., Schrader, K., Regier, D. A., Weymann, D., Chia, S., Gelmon, K., Tinker, A., Sun, S., Lim, H., Renouf, D. J., Laskin, J., Jones, S. J. M., and Marra, M. A. (2020) Pan-cancer analysis of advanced patient tumors reveals interactions between therapy and genomic landscapes. *Nat Cancer* **1**, 452-468

83. Guo, Y. A., Chang, M. M., Huang, W., Ooi, W. F., Xing, M., Tan, P., and Skanderup, A. J. (2018) Mutation hotspots at CTCF binding sites coupled to chromosomal instability in gastrointestinal cancers. *Nat Commun* **9**, 1520
84. Wang, K., Yuen, S. T., Xu, J., Lee, S. P., Yan, H. H., Shi, S. T., Siu, H. C., Deng, S., Chu, K. M., Law, S., Chan, K. H., Chan, A. S., Tsui, W. Y., Ho, S. L., Chan, A. K., Man, J. L., Foglizzo, V., Ng, M. K., Chan, A. S., Ching, Y. P., Cheng, G. H., Xie, T., Fernandez, J., Li, V. S., Clevers, H., Rejto, P. A., Mao, M., and Leung, S. Y. (2014) Whole-genome sequencing and comprehensive molecular profiling identify new driver mutations in gastric cancer. *Nat Genet* **46**, 573-582
85. Dulak, A. M., Stojanov, P., Peng, S., Lawrence, M. S., Fox, C., Stewart, C., Bandla, S., Imamura, Y., Schumacher, S. E., Shefler, E., McKenna, A., Carter, S. L., Cibulskis, K., Sivachenko, A., Saksena, G., Voet, D., Ramos, A. H., Auclair, D., Thompson, K., Sougnez, C., Onofrio, R. C., Guiducci, C., Beroukhim, R., Zhou, Z., Lin, L., Lin, J., Reddy, R., Chang, A., Landrenau, R., Pennathur, A., Ogino, S., Luketich, J. D., Golub, T. R., Gabriel, S. B., Lander, E. S., Beer, D. G., Godfrey, T. E., Getz, G., and Bass, A. J. (2013) Exome and whole-genome sequencing of esophageal adenocarcinoma identifies recurrent driver events and mutational complexity. *Nat Genet* **45**, 478-486
86. Li, C., Sun, Y. D., Yu, G. Y., Cui, J. R., Lou, Z., Zhang, H., Huang, Y., Bai, C. G., Deng, L. L., Liu, P., Zheng, K., Wang, Y. H., Wang, Q. Q., Li, Q. R., Wu, Q. Q., Liu, Q., Shyr, Y., Li, Y. X., Chen, L. N., Wu, J. R., Zhang, W., and Zeng, R. (2020) Integrated Omics of Metastatic Colorectal Cancer. *Cancer Cell* **38**, 734-747 e739
87. Schulze, K., Imbeaud, S., Letouze, E., Alexandrov, L. B., Calderaro, J., Rebouissou, S., Couchy, G., Meiller, C., Shinde, J., Soysouvanh, F., Calatayud, A. L., Pinyol, R., Pelletier, L., Balabaud, C., Laurent, A., Blanc, J. F., Mazzaferro, V., Calvo, F., Villanueva, A., Nault, J. C., Bioulac-Sage, P., Stratton, M. R., Llovet, J. M., and Zucman-Rossi, J. (2015) Exome sequencing of hepatocellular carcinomas identifies new mutational signatures and potential therapeutic targets. *Nat Genet* **47**, 505-511
88. Rudin, C. M., Durinck, S., Stawiski, E. W., Poirier, J. T., Modrusan, Z., Shames, D. S., Bergbower, E. A., Guan, Y., Shin, J., Guillory, J., Rivers, C. S., Foo, C. K., Bhatt, D., Stinson, J., Gnad, F., Haverty, P. M., Gentleman, R., Chaudhuri, S., Janakiraman, V., Jaiswal, B. S., Parikh, C., Yuan, W., Zhang, Z., Koeppen, H., Wu, T. D., Stern, H. M., Yauch, R. L., Huffman, K. E., Paskulin, D. D., Illei, P. B., Varella-Garcia, M., Gazdar, A. F., de Sauvage, F. J., Bourgon, R., Minna, J. D., Brock, M. V., and Seshagiri, S. (2012) Comprehensive genomic analysis identifies SOX2 as a frequently amplified gene in small-cell lung cancer. *Nat Genet* **44**, 1111-1116
89. Um, S. W., Joung, J. G., Lee, H., Kim, H., Kim, K. T., Park, J., Hayes, D. N., and Park, W. Y. (2016) Molecular Evolution Patterns in Metastatic Lymph Nodes Reflect the Differential Treatment Response of Advanced Primary Lung Cancer. *Cancer Res* **76**, 6568-6576
90. Kan, Z., Ding, Y., Kim, J., Jung, H. H., Chung, W., Lal, S., Cho, S., Fernandez-Banet, J., Lee, S. K., Kim, S. W., Lee, J. E., Choi, Y. L., Deng, S., Kim, J. Y., Ahn, J. S., Sha, Y., Mu, X. J., Nam, J. Y., Im, Y. H., Lee, S., Park, W. Y., Nam, S. J., and Park, Y. H. (2018) Multi-omics profiling of younger Asian breast cancers reveals distinctive molecular signatures. *Nat Commun* **9**, 1725

91. Chen, J., Yang, H., Teo, A. S. M., Amer, L. B., Sherbaf, F. G., Tan, C. Q., Alvarez, J. J. S., Lu, B., Lim, J. Q., Takano, A., Nahar, R., Lee, Y. Y., Phua, C. Z. J., Chua, K. P., Suteja, L., Chen, P. J., Chang, M. M., Koh, T. P. T., Ong, B. H., Anantham, D., Hsu, A. A. L., Gogna, A., Too, C. W., Aung, Z. W., Lee, Y. F., Wang, L., Lim, T. K. H., Wilm, A., Choi, P. S., Ng, P. Y., Toh, C. K., Lim, W. T., Ma, S., Lim, B., Liu, J., Tam, W. L., Skanderup, A. J., Yeong, J. P. S., Tan, E. H., Creasy, C. L., Tan, D. S. W., Hillmer, A. M., and Zhai, W. (2020) Genomic landscape of lung adenocarcinoma in East Asians. *Nat Genet* **52**, 177-186
92. Pickering, C. R., Zhou, J. H., Lee, J. J., Drummond, J. A., Peng, S. A., Saade, R. E., Tsai, K. Y., Curry, J. L., Tetzlaff, M. T., Lai, S. Y., Yu, J., Muzny, D. M., Doddapaneni, H., Shinbrot, E., Covington, K. R., Zhang, J., Seth, S., Caulin, C., Clayman, G. L., El-Naggar, A. K., Gibbs, R. A., Weber, R. S., Myers, J. N., Wheeler, D. A., and Frederick, M. J. (2014) Mutational landscape of aggressive cutaneous squamous cell carcinoma. *Clin Cancer Res* **20**, 6582-6592
93. Durinck, S., Stawiski, E. W., Pavia-Jimenez, A., Modrusan, Z., Kapur, P., Jaiswal, B. S., Zhang, N., Toffessi-Tcheuyap, V., Nguyen, T. T., Pahuja, K. B., Chen, Y. J., Saleem, S., Chaudhuri, S., Heldens, S., Jackson, M., Pena-Llopis, S., Guillory, J., Toy, K., Ha, C., Harris, C. J., Holloman, E., Hill, H. M., Stinson, J., Rivers, C. S., Janakiraman, V., Wang, W., Kinch, L. N., Grishin, N. V., Haverty, P. M., Chow, B., Gehring, J. S., Reeder, J., Pau, G., Wu, T. D., Margulis, V., Lotan, Y., Sagalowsky, A., Pedrosa, I., de Sauvage, F. J., Brugarolas, J., and Seshagiri, S. (2015) Spectrum of diverse genomic alterations define non-clear cell renal carcinoma subtypes. *Nat Genet* **47**, 13-21
94. Stransky, N., Egloff, A. M., Tward, A. D., Kostic, A. D., Cibulskis, K., Sivachenko, A., Kryukov, G. V., Lawrence, M. S., Sougnez, C., McKenna, A., Shefler, E., Ramos, A. H., Stojanov, P., Carter, S. L., Voet, D., Cortes, M. L., Auclair, D., Berger, M. F., Saksena, G., Guiducci, C., Onofrio, R. C., Parkin, M., Romkes, M., Weissfeld, J. L., Seethala, R. R., Wang, L., Rangel-Escareno, C., Fernandez-Lopez, J. C., Hidalgo-Miranda, A., Melendez-Zajgla, J., Winckler, W., Ardlie, K., Gabriel, S. B., Meyerson, M., Lander, E. S., Getz, G., Golub, T. R., Garraway, L. A., and Grandis, J. R. (2011) The mutational landscape of head and neck squamous cell carcinoma. *Science* **333**, 1157-1160
95. Rokita, J. L., Rathi, K. S., Cardenas, M. F., Upton, K. A., Jayaseelan, J., Cross, K. L., Pfeil, J., Egolf, L. E., Way, G. P., Farrel, A., Kendsersky, N. M., Patel, K., Gaonkar, K. S., Modi, A., Berko, E. R., Lopez, G., Vaksman, Z., Mayoh, C., Nance, J., McCoy, K., Haber, M., Evans, K., McCalmont, H., Bendak, K., Bohm, J. W., Marshall, G. M., Tyrrell, V., Kalletla, K., Braun, F. K., Qi, L., Du, Y., Zhang, H., Lindsay, H. B., Zhao, S., Shu, J., Baxter, P., Morton, C., Kurmashev, D., Zheng, S., Chen, Y., Bowen, J., Bryan, A. C., Leraas, K. M., Coppens, S. E., Doddapaneni, H., Momin, Z., Zhang, W., Sacks, G. I., Hart, L. S., Krytska, K., Mosse, Y. P., Gatto, G. J., Sanchez, Y., Greene, C. S., Diskin, S. J., Vaske, O. M., Haussler, D., Gastier-Foster, J. M., Kolb, E. A., Gorlick, R., Li, X. N., Reynolds, C. P., Kurmasheva, R. T., Houghton, P. J., Smith, M. A., Lock, R. B., Raman, P., Wheeler, D. A., and Maris, J. M. (2019) Genomic Profiling of Childhood Tumor Patient-Derived Xenograft Models to Enable Rational Clinical Trial Design. *Cell Rep* **29**, 1675-1689 e1679
96. Hyman, D. M., Piha-Paul, S. A., Won, H., Rodon, J., Saura, C., Shapiro, G. I., Juric, D., Quinn, D. I., Moreno, V., Doger, B., Mayer, I. A., Boni, V., Calvo, E., Loi, S., Lockhart, A. C., Erinjeri, J. P., Scaltriti, M., Ulaner, G. A., Patel, J., Tang, J., Beer, H., Selcuklu, S. D., Hanrahan, A. J., Bouvier, N., Melcer, M., Murali, R., Schram, A. M., Smyth, L. M., Jhaveri, K., Li, B. T., Drilon, A., Harding, J. J., Iyer, G., Taylor, B. S., Berger, M. F., Cutler, R. E., Jr., Xu, F., Butturini, A., Eli, L. D., Mann, G., Farrell, C., Lalani, A. S., Bryce, R. P., Arteaga, C. L., Meric-Bernstam, F., Baselga, J., and Solit, D. B. (2018)

HER kinase inhibition in patients with HER2- and HER3-mutant cancers. *Nature* **554**, 189-194

97. Miller, A. M., Shah, R. H., Pentsova, E. I., Pourmaleki, M., Briggs, S., Distefano, N., Zheng, Y., Skakodub, A., Mehta, S. A., Campos, C., Hsieh, W. Y., Selcuklu, S. D., Ling, L., Meng, F., Jing, X., Samoila, A., Bale, T. A., Tsui, D. W. Y., Grommes, C., Viale, A., Souweidane, M. M., Tabar, V., Brennan, C. W., Reiner, A. S., Rosenblum, M., Panageas, K. S., DeAngelis, L. M., Young, R. J., Berger, M. F., and Mellinghoff, I. K. (2019) Tracking tumour evolution in glioma through liquid biopsies of cerebrospinal fluid. *Nature* **565**, 654-658
98. Grasso, C. S., Wu, Y. M., Robinson, D. R., Cao, X., Dhanasekaran, S. M., Khan, A. P., Quist, M. J., Jing, X., Lonigro, R. J., Brenner, J. C., Asangani, I. A., Ateeq, B., Chun, S. Y., Siddiqui, J., Sam, L., Anstett, M., Mehra, R., Prensner, J. R., Palanisamy, N., Ryslik, G. A., Vandin, F., Raphael, B. J., Kunju, L. P., Rhodes, D. R., Pienta, K. J., Chinnaiyan, A. M., and Tomlins, S. A. (2012) The mutational landscape of lethal castration-resistant prostate cancer. *Nature* **487**, 239-243
99. Agrawal, N., Frederick, M. J., Pickering, C. R., Bettegowda, C., Chang, K., Li, R. J., Fakhry, C., Xie, T. X., Zhang, J., Wang, J., Zhang, N., El-Naggar, A. K., Jasser, S. A., Weinstein, J. N., Trevino, L., Drummond, J. A., Muzny, D. M., Wu, Y., Wood, L. D., Hruban, R. H., Westra, W. H., Koch, W. M., Califano, J. A., Gibbs, R. A., Sidransky, D., Vogelstein, B., Velculescu, V. E., Papadopoulos, N., Wheeler, D. A., Kinzler, K. W., and Myers, J. N. (2011) Exome sequencing of head and neck squamous cell carcinoma reveals inactivating mutations in NOTCH1. *Science* **333**, 1154-1157
100. Liu, D., Schilling, B., Liu, D., Sucker, A., Livingstone, E., Jerby-Arnon, L., Zimmer, L., Gutzmer, R., Satzger, I., Loquai, C., Grabbe, S., Vokes, N., Margolis, C. A., Conway, J., He, M. X., Elmarakeby, H., Dietlein, F., Miao, D., Tracy, A., Gogas, H., Goldinger, S. M., Utikal, J., Blank, C. U., Rauschenberg, R., von Bubnoff, D., Krackhardt, A., Weide, B., Haferkamp, S., Kiecker, F., Izar, B., Garraway, L., Regev, A., Flaherty, K., Paschen, A., Van Allen, E. M., and Schadendorf, D. (2019) Integrative molecular and clinical modeling of clinical outcomes to PD1 blockade in patients with metastatic melanoma. *Nat Med* **25**, 1916-1927
101. Stiegler, A. L., Vish, K. J., and Boggon, T. J. (2022) Tandem engagement of phosphotyrosines by the dual SH2 domains of p120RasGAP. *Structure* **30**, 1603-1614 e1605
102. Paul, M. E., Chen, D., Vish, K. J., Lartey, N. L., Hughes, E., Freeman, Z. T., Saunders, T. L., Stiegler, A. L., King, P. D., and Boggon, T. J. (2025) The C2 domain augments Ras GTPase-activating protein catalytic activity. *Proc Natl Acad Sci U S A* **122**, e2418433122
103. Crooks, G. E., Hon, G., Chandonia, J. M., and Brenner, S. E. (2004) WebLogo: a sequence logo generator. *Genome Res* **14**, 1188-1190
